# Design and Synthesis of C-19 Isosteviol Derivatives as Potent and Highly Selective Antiproliferative Agents

**DOI:** 10.3390/molecules24010121

**Published:** 2018-12-30

**Authors:** Tian Luan, Li-Hua Cao, Hao Deng, Qing-Kun Shen, Yu-Shun Tian, Zhe-Shan Quan

**Affiliations:** 1 Key Laboratory of Natural Resources and Functional Molecules of the Changbai Mountain, Affiliated Ministry of Education, College of Pharmacy, Yanbian University, Yanji 133002, China; sapphire0614@163.com (T.L.); 2017010676@ybu.edu.cn (H.D.); 2016001065@ybu.edu.cn (Q.-K.S.); 2 Department of Pharmacology, College of Medicine, Yanbian University, Yanji 133002, China; lhcao@ybu.edu.cn

**Keywords:** isosteviol, derivatives, antiproliferation, HCT-116

## Abstract

Six series of novel isosteviol derivatives; modified in the C-19 position; were synthesized; and their antiproliferative activity was evaluated against three human cancer cell lines (HCT-116; BEL-7402; HepG2) and the human L02 normal cell line in vitro. Most of the derivatives tested here exhibited improved antiproliferative activity with high selectivity when compared with the parent compound isosteviol and the positive control drug 5-fluorouracil. Among these derivatives; compound **5d** exhibited the most potent antiproliferative activity and commendable selectivity between cancer and normal cells. In addition; compound **5d** inhibited the colony formation of HCT-116 cells in a concentration-dependent manner. Further studies revealed that compound **5d** arrested the HCT-116 cell cycle in the S phase; and western blot analysis demonstrated the mechanism may be correlated with a change in the expression of cyclin A; cyclin B1; and cyclin E1. Furthermore; the results of a docking study that involved placing compound **5d** into the CDK2/cyclin A binding site revealed that its mode of action was possibly as a CDK2/cyclin A inhibitor.

## 1. Introduction

Cancer is one of the most dangerous, fast propagating diseases of the present century. Despite the enormous advances in cancer treatment, it remains the second most common cause of death worldwide because of ineffective chemotherapy, caused by drug resistance and the inability of many drugs to differentiate between cancerous cells and normal cells [1,2]. In this background, natural products have proven the utility as the core sources of novel composition, the percentage of natural product based drugs into the market was increasing from the past years, which makes it an attractive source of cancer drug discovery [3]. Therefore, the discovery of potent and highly selective derivatives through structural modification of natural products in the development of anticancer drugs is promising.

**Figure 1:**
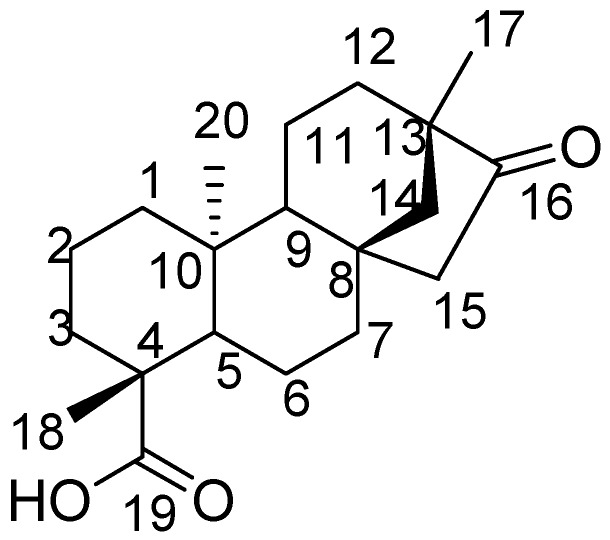
The chemical structure and atom number of isosteviol.

The diterpene, isosteviol (Figure 1), bearing the distinctive tetracyclic skeleton of kaurene, exhibits wide biological activities including blood lipid control [4], anti-hypertension [5], heart-brain cell protection [6,7], blood glucose reduction [8,9] and antibacterial [10] and antiinflammation [11,12] effects [13,14,15,16]. It can be obtained as a metabolite of stevioside isolated from the leaves of the natural *stevia* plant [17]. Furthermore, the cytotoxic activities of isosteviol derivatives have attracted much attention in recent years. In the previous study, the C-15 and C-16 functionalized isosteviol derivatives, obtained by means of group-conversion or structural modification exhibited good cytotoxic activities [17,18,19,20,21,22,23,24]. In view of the low cytotoxicity of isosteviol, it is suitable for the development of highly selective anticancer drugs by chemical modification [14,15,16]. In addition, to the best of our knowledge, the antiproliferative mechanism of isosteviol derivatives such as cell cycle, cell apoptosis and related markers have not been reported. Therefore, in the present work, we introduced different crucial fragment in the C-19 of isosteviol in order to obtain some compounds with significantly improved antiproliferative activity and highly selectivity.

**Figure 2:**
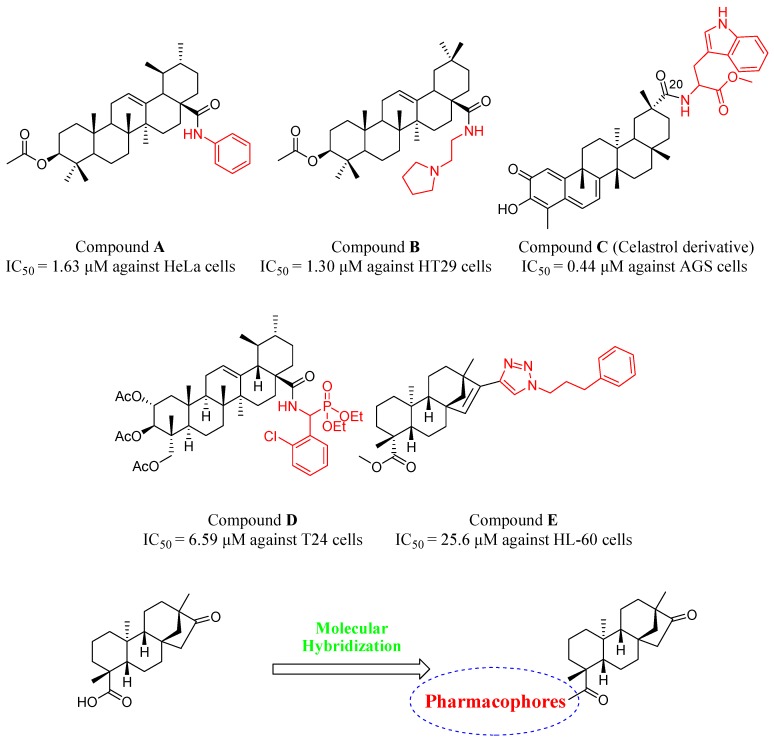
Design of target compounds based on the combination principles (The black part represents the parent compound and the red part represents the pharmacophore).

Aniline and heterocyclic fragments are common pharmacophores that display diverse biological functions, especially antiproliferative activities. Many reports have shown that introducing aniline and heterocyclic fragments in different natural products can improve their antiproliferative activity. As shown in Figure 2, compound **A** and **B** exhibited significant in vitro antiproliferative potency against the HeLa and HT24 cell lines with IC_50_ values of 1.63 μM and 1.3 μM, respectively [25,26,27,28]. Amino acids are important organic compounds that have many functions in metabolism and are defined by their unique side chain. Due to their structural diversity, amino acids provide a balance between hydrophilicity and hydrophobicity which is necessary for the cell membrane solubility and permeability [29]. Our previous study investigated the structure-activity relationship of the antiproliferative effects of celastrol analogues. Compound **C** which has a tryptophan methyl ester introduced in the 20th carboxylic acid position, inhibited cell proliferation of AGS cells with an IC_50_ value of 0.44 μM [23]. Similarly, a drug with a phosphonate introduced can display enhanced solubility and drug-like properties by regulating the distribution coefficient. Researchers have combined a variety of different natural products with these pharmacophores and found that their derivatives play an important role in antiproliferative activity [30,31]. Among them, compound **D** exhibited significantly improved antiproliferative activity when compared with the parent compound asiatic acid [30]. Interestingly, there are many drug discovery initiatives where triazole has been successfully conjugated with biologically active cores, showing potent antiproliferative activity. Khaybullin et al. reported that a series of isosteviol derived triazole D ring conjugates facilitated the development of potential antiproliferative agents and as the result, some derivatives showed promising antiproliferative activities against different types of cancer cell lines such as compound **E** [20,32].

Based on the combination principles of drugs, the aforementioned findings stimulated our interest in designing and synthesizing six series of novel isosteviol derivatives, linking these pharmacophores in the C-19 position. The antiproliferative activity of the target compounds was evaluated on human colorectal cancer (HCT-116), human hepatocellular carcinoma (BEL-7402), human liver cancer (HepG2) and human normal liver cells (L02). Furthermore, we chose the antiproliferative activity of the derivative with the strongest antiproliferative activity and investigated its possible mechanism of action. Finally, molecular docking analysis has also been performed to support the effective binding of the compound at the active site of the protein.

## 2. Results and Discussion

### 2.1. Chemistry

**Scheme 1:**
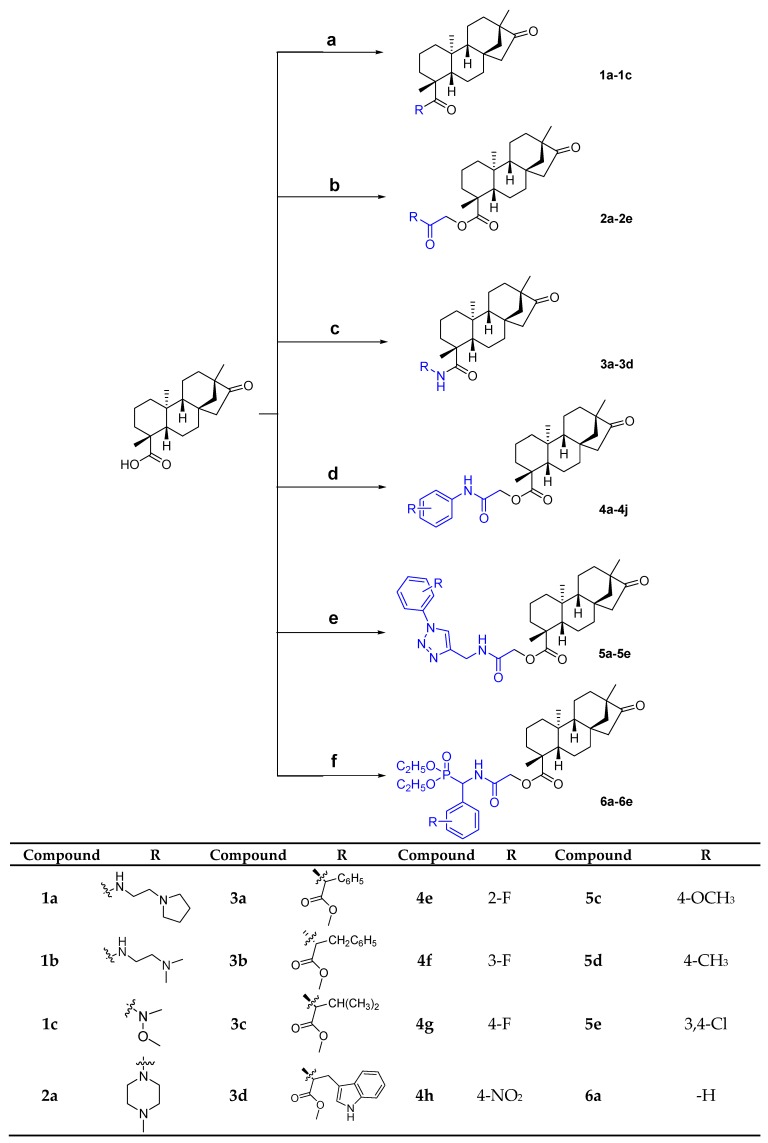

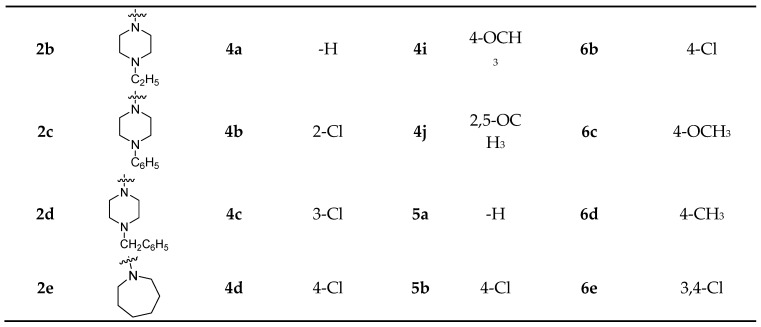
Reagents and conditions: (**a**) (i) oxalyl chloride, CHCl_3_, 60 °C; (ii) IIa-IIe, Et_3_N, CH_2_Cl_2_, 30 °C. (**b**) different heterocyclic chloroacetamides, K_2_CO_3_, CH_3_CN, 80 °C. (**c**) different amino acid esters, EDC∙HCl, HOBt, Et_3_N, CHCl_3_, 60 °C. (**d**) IVa-IVj, K_2_CO_3_, CH_3_CN, 80 °C. (**e**) Va-Ve, K_2_CO_3_, CH_3_CN, 80 °C. (**f**) VIa-VIe, K_2_CO_3_, CH_3_CN, 80 °C.

The synthetic procedure adopted to obtain the target compounds is shown in Scheme 1. The reaction occurred at the C-19 position of isosteviol. Compounds **1a**–**1c** are products of the reaction of isosteviol chloride with different amines at 30 °C and compounds **3a**–**3d** were obtained by an amide condensation reaction with different amino acid esters, catalyzed by EDC∙HCl, HOBt and Et_3_N in anhydrous CHCl_3_ at 60 °C. The yields of **1a**–**1c** were in the range of 66–71% and those of **3a**–**3d** were in the range of 70–76%. All of the other isosteviol derivatives (**2a**–**2e**, **4a**–**4j**, **5a**–**5e** and **6a**–**6e**) were generated from various chlorinated derivatives via nucleophilic substitution in good to excellent yields (78–90%). We chose methylenecarbonyl group as a linker between isosteviol and these pharmacophores. While the carbonyl group of the linker can be used to introduce into a hydrogen bond receptor, finishing with a nucleophilic substitution reaction can make full use of starting material isosteviol and significantly increase the yield of products compared to condensation reaction. Before biological evaluation, all target compounds were characterized via ^1^H-NMR, ^13^C-NMR and high-resolution mass spectrometry. 1H and 13C-NMR spectra of these compounds are available in the supplementary materials.

### 2.2. In Vitro Antiproliferative Activity

As shown in Table 1, thirty-two target compounds were evaluated for their antiproliferative activities in vitro against HCT-116, BEL-7402 and HepG2 cell lines. The activity of isosteviol was used as reference. With the exception of compounds **1c**, **2b**, **2c**, **3d**, **4b**, **4c**, **4e**, **4i** and **4j**, all target compounds exhibited stronger antiproliferative activity against three different human cancer cell lines than the lead compound isosteviol at 100 μM. However, some compounds lost their antiproliferative activity at lower concentrations and half of the target compounds had a lower IC_50_ value (<100 μM) than the lead compound isosteviol against the cancer cell lines.

As shown in Table 2, all of these compounds showed moderate to significant activity against all three cancer cell lines, improved potency up to one-two digits μM compared with isosteviol. Among the compounds **1a**–**1c**, which react with amines compounds, only compound **1b** exhibited slightly higher antiproliferative activity against the HCT-116 cell line (IC_50_ = 24.04 ± 1.20 μM) compared with the positive-control drug 5-FU. Compounds **2a**–**2e** are products of a reaction between isosteviol and heterocyclic chloroacetamides. In this series of compounds, compound **2d** exhibited higher antiproliferative activity against the HCT-116 and HepG2 cell lines (IC_50_ value of 18.18 ± 1.26 μM and 11.05 ± 0.55, respectively) and compound **2e** exhibited higher antiproliferative activity against the BEL-7402 and HepG2 cell lines (IC_50_ value of 13.22 ± 0.32 μM and 20.51 ± 0.46, respectively) when compared with 5-FU. Compounds **3a**–**3d** were generated from amino acid esters. Only compound **3b**, which has a D-phenylalanine methyl ester introduced in the C-19 position, displayed similar antiproliferative activity against the three cancer cell lines (IC_50_ = 22.78 ± 0.81 μM against HCT-116 cells, IC_50_ = 36.49 ± 0.67 μM against BEL-7402 cells and IC_50_ = 24.40 ±1.75 μM against HepG2 cells). Compounds **4a**–**4j** are products of a reaction between isosteviol and various chloroacetanilides. Among them, compound **4f** showed selective cytotoxic effects against the HCT-116 cell line, with an IC_50_ value of 17.22 ± 0.43 μM, while compound **4h** displayed better antiproliferative activity against the BEL-7402 and HepG2 cell lines (IC_50_ = 12.14 ± 0.12 μM and IC_50_ = 14.11 ± 0.27 μM, respectively). Thus, compounds with an electron-withdrawing group (–F, –NO_2_) at the phenyl moiety displayed better antiproliferative activity. Compounds **5a**–**5e** have different phenyl 1,2,3-triazole chloroacetamides compounds substituted. All of these compounds showed considerably higher antiproliferative activity against the HCT-116 and HepG2 cell lines. Perhaps the triazole acts as a hydrogen bond acceptor and binds to some key enzymes involved in cancer cell metabolism, inhibiting their expression. Compound **5a** and **5b** exhibited significant in vitro antiproliferative potency against the HepG2 and HCT-116 cell lines with IC_50_ values of 9.87 ± 0.13 μM and 7.44 ± 0.37 μM, respectively. Compound **5d**, which has a methyl group introduced at the *para* position of the benzene ring, had the strongest antiproliferative activity among all the target compounds (IC_50_ value of 5.38 ± 0.26 μM, 15.91 ± 0.41 μM and 8.92 ± 0.44 μM against HCT-116, BEL-7402 and HepG2 cell lines, respectively). It was 4.6-fold (against HCT-116 cells), 1.3-fold (against BEL-7402 cells) and 2.6-fold (against HepG2 cells) more active than 5-FU. Also, it was 18.6-fold (against HCT-116 cells), 6.3-fold (against BEL-7402 cells) and 11.2-fold (against HepG2 cells) more active than lead compound isosteviol at least. Compounds **6a**–**6e**, were generated by reactions with different phosphonic acid [[(chloroacetyl) amino] phenylmethyl]-diethyl esters. Compound **6c** showed higher antiproliferative activity against the HCT-116 cell line (IC_50_ = 15.15 ± 0.11 μM) and compound **6d** showed better antiproliferative activity against the HCT-116 and HepG2 cell lines (IC_50_ = 18.70 ± 0.17 and 13.19 ± 0.86 μM, respectively). However, compounds **6a**, **6b** and **6e** lost their antiproliferative activity. It seems that the antiproliferative ability was enhanced after the introduction of electron-donating groups to the *para* position of the benzene ring.

It is worth mentioning that all the target compounds exhibited low cytotoxic activities against human normal liver cells (L02), with remarkable selectivity (IC_50_ > 100 μM), which supported the rationale of the drug design. Above all, we chose to investigate the mechanism of action for compound **5d** because it had the strongest antiproliferative activity against HCT-116 cell line.

### 2.3. Selective Inhibition of Cancer Cell Growth by Compounds **5d**

Lack of selective cytotoxicity is the main factor that restricts the conventional chemotherapeutic agents [29,33]. Thus, to evaluate the selective antiproliferative activity of the compound **5d**, the selectivity index (SI) between cancer and normal cells was calculated and the results are summarized in Table 3. The SI was calculated by the dividing the IC_50_ values in normal cells by the IC_50_ values in cancer cells. 5-FU had similar effects and toxic doses, with SI values of 0.77 (HCT-116), 0.90 (BEL-7402) and 0.82 (HepG2), respectively, indicating that both normal cells and cancer cells would be killed. Whereas, compound **5d** exhibited a 38.17-fold, 11.24-fold and 22.01-fold higher selectivity for HCT-116, BEL-7402 and HepG2 cells, respectively. It means that **5d** has much better therapeutic activity and specificity than 5-FU.

### 2.4. Compound **5d** Inhibited HCT-116 Cells Colony Formation

**Figure 3:**
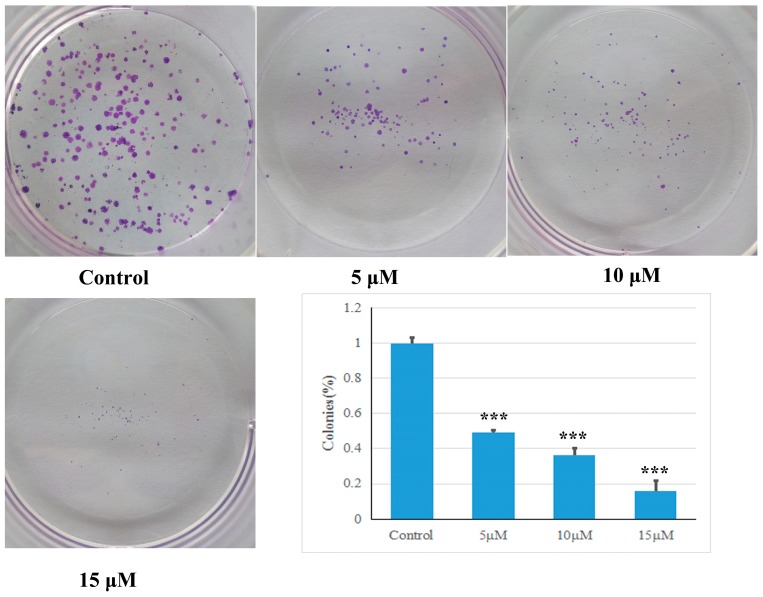
The colony formation of HCT-116 cells after treatment with different concentration (5, 10 and 15 μM) of compound **5d** for 7 days; The rate of inhibition induced by compound **5d** was expressed as the mean ± SD of three independent experiments, *** *p* < 0.001 vs control group (untreated cells); The clonogenicity assay was quantified using Image *J* software.

Colony formation represents the malignant potential, which is a basic characteristic of cancer cells. Colony formation assay is used to test cells for their ability to form colonies and undergo unlimited division [34]. The combined results of three independent experiments are depicted in Figure 3. The exposure of HCT-116 cells to compound **5d** resulted in a significant suppression of colony formation in a concentration-dependent manner. It led to fewer and smaller colonies compared to the control with an inhibition rate of 84% at 15 μM and colony formation was almost completely suppressed. These results demonstrate that compound **5d** can significantly inhibit the proliferation of HCT-116 cells.

### 2.5. Analysis for Cell Cycle and Apoptosis by Flow Cytometry

**Figure 4:**
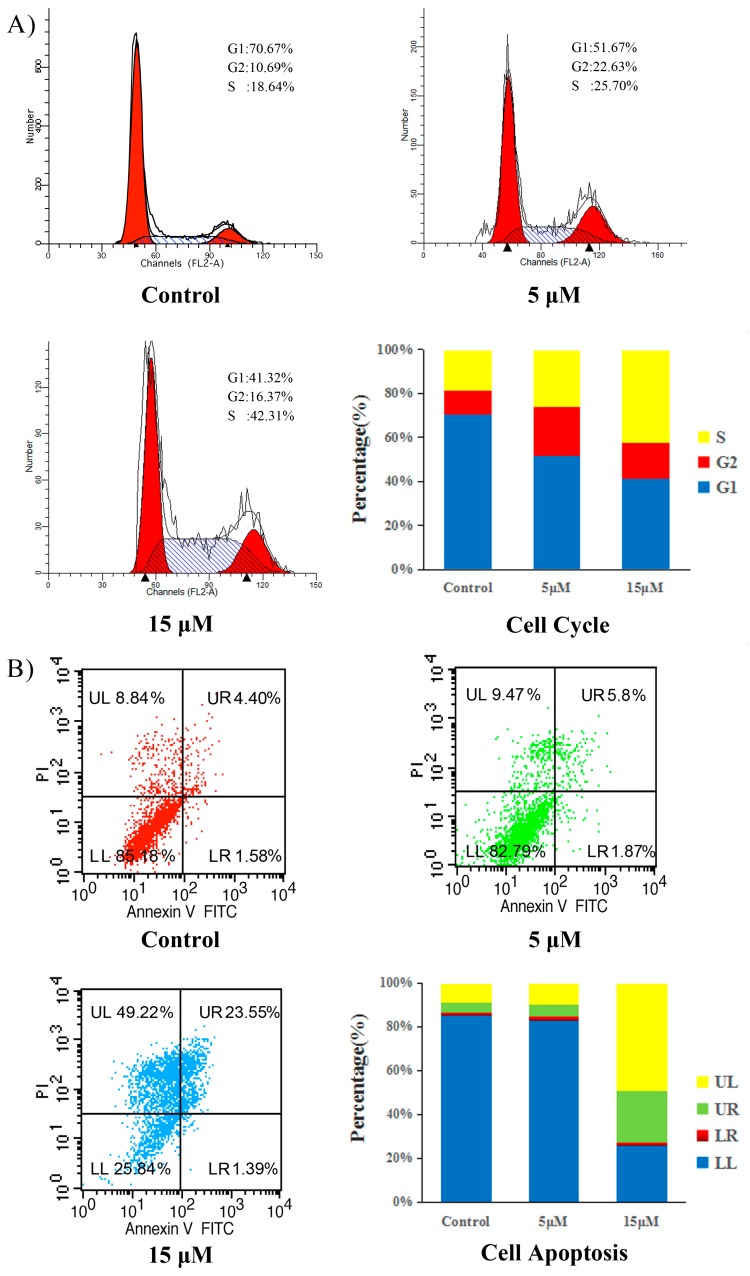
(**A**): Flow cytometry analyses of cell cycle distribution of HCT-116 cells after treatment of compound **5d** (5 and 15 μM) and no treatment (Ctrl) as a reference for 24 h; (**B**): Apoptosis induction in HCT-116 cells after treatment of compound **5d** (5 and 15 μM) and no treatment (Ctrl) as a reference for 24 h.

Cell cycle dysregulation resulting in mitogenic signaling and leading to uncontrolled proliferation is one of the hallmarks of cancer. Also, inducing apoptosis is one of the protective mechanisms used against cancer initiation and progression, which is a significant goal of anticancer therapy. Many cytotoxic compounds exert their antiproliferative effect by the arrest of the cell cycle at a particular cell cycle checkpoint, the induction of apoptosis or by a combination of these two effects. This type of mechanism is considered to be an effective anticancer strategy [35,36]. Therefore, in order to identify whether compound **5d** inhibited cell proliferation through cell cycle arrest or apoptosis, we assayed its ability to trigger cell cycle arrest and apoptosis in HCT-116 cells by flow cytometry. As shown in Figure 4A), the proportion of cells in the G1 phase decreased from 70.67% in the control to 51.67% and 41.32% when treated with 5 and 15 μM compound **5d**, respectively and the proportion of cells in the S phase dramatically increased in a concentration-dependent manner, from 18.64% (control) to 25.70% (5 μM) and 42.31% (15 μM), this implies that the cell is unable to duplicate its DNA. The results indicate that compound **5d** arrests cells in the S phase of the cell cycle. 

To determine whether antiproliferation caused by compound **5d** was due to increasing of apoptosis, annexin V-FITC/propidium iodide assay was used to investigate apoptosis. As shown in Figure 4B), at 5 μM, the percentage of total apoptotic cells (right quadrants, UR + LR) increased inconsiderably, from 5.98% (control) to 7.67%. After increasing the concentration of the drug to 15 μM, the percentage of total apoptotic cells increased to 24.94%. However, we have found that the number of dead cells (UL) also increased from 8.84% (control) to 49.22% (15 μM). It is likely that compound **5d** causes cell death by inducing apoptosis in combination with other ways. These results indicate that compound **5d** have a weaker influence on cell apoptosis.

### 2.6. Western Blot Analysis

**Figure 5:**
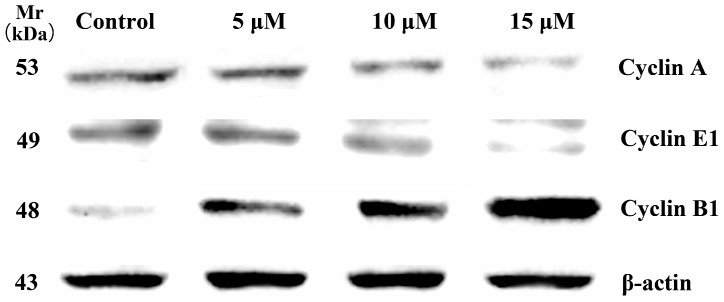
Western blot analysis: Effects of Cyclin A, Cyclin E1 and Cyclin B1 in HCT-116 cells after 24 h treatment with compound **5d** at 5, 10 and 15 μM. β-actin served as an internal control.

It is known that cell proliferation is generally regulated by controlling cell cycle progression through inhibition or promotion of activities of cyclins and their associated proteins. Cyclins play a significant role in cell cycle progression by activation of ATP binding site [37]. Cyclin E helps in the Rb/E2F transcription via formation of a complex with the CDK2 and controls G1 phase [38]. Cyclin A forms a complex with CDK2 and helps in the regulation of S phase [37]. Cyclin B is also called as maturation or mitosis promoting factor as it forms complex with CDK1 and controls the M phase of the cell cycle [39]. Analysis for cell cycle by flow cytometry confirmed that compound **5d** induced arrest in S phase of the HCT-116 cells. However, it had a weaker influence on cell apoptosis. Therefore, the further research on apoptosis-related proteins was abandoned. Since entry into the S phase in the cell cycle requires the accumulation of cell cycle activation-related cyclins, we further examined the effect of compound **5d** on cell cycle regulating proteins such as cyclin A, cyclin B1 and cyclin E1 to determine whether the decreased cell proliferation by **5d** involved these proteins. As shown in Figure 5, in a concentration-dependent manner, compound **5d** decreased cyclin A and cyclin E1 protein levels, while increasing the level of cyclin B1. It has been reported that expression of cyclin B1 is increased by exposure to anticancer agents [40,41]. Hence, these results suggest that the up-regulation of cyclin B1 and down-regulation of cyclins A and E1 are related to the decrease in cell proliferation and S phase arrest by **5d** treatment on HCT-116 cells.

### 2.7. Molecular Docking Analysis

**Figure 6:**
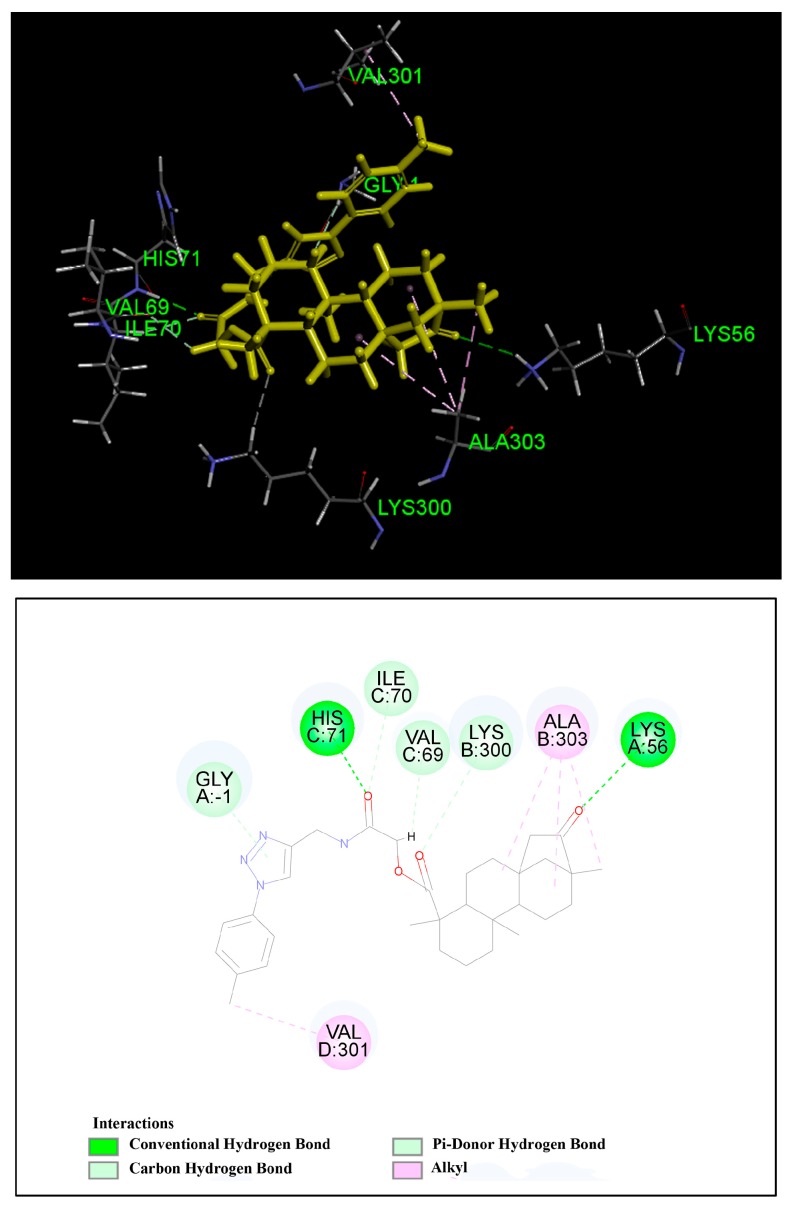
Computer modelling of compound **5d** binding to CDK2/cyclin A (3my5). Compound **5d** was colored in yellow.

Our cell cycle study revealed compound **5d** induced arrest in S phase of the HCT-116 cells and western blot analysis demonstrated the mechanism of action may be correlated with down-regulation of cyclin A, in addition, cyclin A and CDK2 form a complex that plays a key role in the regulation of the S phase. Therefore, in order to better understand the potency of compound **5d** and to gain further understanding of the probable binding model, molecular docking studies with CDK2/cyclin A (PDB ID: 3my5) were performed. The studies were performed as a crucial step towards understanding the specific position of interaction between compound **5d** and CDK2/cyclin A complex [42]. The docking results revealed that compound **5d** was held in the active pocket by a combination of various hydrogen bonds and alkyl interactions with CDK2/cyclin A (Figure 6). The carbonyl group of isosteviol interacted with the -NH_2_ group of Lys-A56 via hydrogen bond, while the carbonyl group of 1,2,3-triazole chloroacetamides interacted with the -NH- moiety of Lys-A56 via hydrogen bond, which supported the preceding drug design. Meanwhile, the 1,2,3-triazole moiety formed one pi-donor hydrogen bond with Gly-A1 residue, as well as the methylenecarbonyl group and the carbonyl group of C-19 formed three carbon hydrogen bond with Ile-C70, Val-C69 and Lys-B300 amino acids. Additionally, the methyl group at the *para* position of the benzene ring formed a favorable alkyl interaction with Val-D301 residue. It was also observed that the B and C ring of isosteviol showed alkyl interaction with amino acid residue Ala-B303. The CDOCKER interaction energy score was 41.3422. These indicate that compound **5d** has a strong binding affinity for the CDK2/cyclin A complex and may play a crucial role in inhibiting its activity.

## 3. Materials and Methods

### 3.1. General Procedures

Melting points were determined in open capillary tubes and were uncorrected. Reactions were monitored by thin-layer chromatography (TLC) on silica gel plates. ^1^H-NMR and ^13^C-NMR spectra were measured on an AV-300 (Bruker BioSpin, Fällanden, Switzerland) and AV-500 (Bruker BioSpin, Fällanden, Switzerland) and all chemical shifts were given in ppm relative to tetramethylsilane (TMS). High-resolution mass spectra were measured using a matrix-assisted laser desorption/ionization (MALDI)-time of flight (TOF)/TOF mass spectrometer (Bruker Daltonik, Bremen, Germany). The major chemicals were purchased from Aldrich Chemical Corporation (Milwaukee, WI, USA). All other chemicals were of analytical grade.

### 3.2. General Procedure for the Synthesis of Compound (**1a**–**1c**)

Isosteviol (63.6 mg, 0.20 mmol) was placed in CHCl_3_ (10 mL), oxalyl chloride (38.1 mg, 0.30 mmol) was added and the mixture was stirred at 60 °C for 2 h. Upon completion, the solvent was evaporated in vacuo to obtain the crude product, a colorless crystal, which was used in the next step without further purification. Next, the crude product and different amines (0.21 mmol) and Et_3_N (22.2 mg, 0.22 mmol) were added to CH_2_Cl_2_ (10 mL) and the resulting mixture was stirred at 30 °C for 2 h. Then, the solvent was evaporated in vacuo, water was added (10 mL), the mixture was filtered and the residue was washed with water to obtain the target compounds (**1a**–**1c**) as white powders.

*(4R,4aS,6aR,9S,11aR,11bS)-4,9,11b-trimethyl-8-oxo-N-(2-(pyrrolidin-1-yl)ethyl)tetradecahydro-6a,9-methanocyclohepta[a]naphthalene-4-carboxamide* (**1a**). White powder; yield, 71%; m.p. 136–137 °C; ^1^H-NMR (CDCl_3_, 300 MHz, ppm): δ 6.34 (brs, 1H, -CO-NH-), 3.31 (q, *J* = 6 Hz, 2H, -CO-NH-CH_2_-), 2.70–2.51 (m, 7H, N(CH_2_)_3_, 15-He), 2.10 (d, *J* = 12 Hz, 1H, 3-He), 2.00–1.95 (m, 1H), 1.89–1.86 (m, 1H), 1.83–1.75 (m, 8H), 1.71–1.64 (m, 2H), 1.60–1.59 (m, 1H), 1.55 (s, 1H), 1.50–1.49 (m, 1H), 1.45–1.44 (m, 1H), 1.41–1.38 (m, 1H), 1.35–1.23 (m, 2H), 1.19 (s, 3H, 18-CH_3_), 1.16–1.07 (m, 2H), 0.99 (s, 3H, 17-CH_3_), 0.95–0.89 (m, 1H), 0.79 (s, 3H, 20-CH_3_). ^13^C-NMR (CDCl_3_, 75 MHz, ppm): δ 222.40, 176.70, 57.49, 54.75, 54.26, 53.78, 53.61(2C), 48.71, 48.38, 43.61, 41.80, 40.26, 39.50, 38.11(2C), 37.92, 37.31, 30.21, 23.63(2C), 22.01, 20.33, 19.86, 19.04, 13.41. HRMS (*m*/*z*): calcd for C_26_H_43_N_2_O_2_^+^ [M + H]^+^: 415.3319, found: 415.3316.

*(4R,4aS,6aR,9S,11aR,11bS)-N-(2-(dimethylamino)ethyl)-4,9,11b-trimethyl-8-oxotetradecahydro-6a,9-methanocyclohepta[a]naphthalene-4-carboxamide* (**1b**). White powder; yield, 66%; m.p. 102–103 °C; ^1^H-NMR (CDCl_3_, 300 MHz, ppm): δ 6.30 (brs, 1H, -CO-NH-), 2.29 (q, *J* = 6 Hz, 2H, -CO-NH-CH_2_-), 2.67 (dd, *J* = 18, 3 Hz, 1H, 15-He), 2.41 (t, *J* = 6 Hz, 2H, -CO-NH-CH_2_-), 2.29–2.19 (m, 6H, N(CH_2_)_3_), 2.09 (d, *J* = 12 Hz, 1H, 3-He), 2.02–1.94 (m, 1H, 6-He), 1.88–1.78 (m, 3H), 1.76–1.68 (m, 3H), 1.64–1.59 (m, 2H), 1.55–1.48 (m, 2H), 1.45–1.38 (m, 3H), 1.36–1.31 (m, 1H), 1.27 (s, 3H, 18-CH_3_), 1.16–1.09 (m, 2H), 0.99 (s, 3H, 17-CH_3_), 0.95–0.90 (m, 1H), 0.79 (s, 3H, 20-CH_3_). ^13^C-NMR (CDCl_3_, 75 MHz, ppm): δ 222.43, 176.71, 57.55(2C), 54.75, 54.28, 48.71, 48.37, 45.09(2C), 43.62, 41.79, 40.25, 39.51, 38.09(2C), 37.32, 36.73, 30.18, 22.04, 20.34, 19.86, 19.11, 13.44. HRMS (*m*/*z*): calcd for C_24_H_41_N_2_O_2_^+^ [M + H]^+^: 389.3163, found: 389.3166.

*(4R,4aS,6aR,9S,11aR,11bS)-N-methoxy-N,4,9,11b-tetramethyl-8-oxotetradecahydro-6a,9-methanocyclohepta[a]naphthalene-4-carboxamide* (**1c**). White powder; yield, 68%; m.p. 144–145 °C; ^1^H-NMR (CDCl_3_, 300 MHz, ppm): δ 3.66 (s, 3H, -O-CH_3_), 3.12 (s, 3H, -N-CH_3_), 2.73 (dd, *J* = 18, 3 Hz, 1H, 15-He), 2.53 (d, *J* = 12 Hz, 1H, 3-He), 2.16–2.02 (m, 1H, 6-He), 1.90–1.78 (m, 2H), 1.74–1.58 (m, 6H), 1.54–1.34 (m, 5H), 1.28 (s, 3H, 18-CH_3_), 1.22–1.17 (m, 1H), 1.13–1.04 (m, 2H), 0.99 (s, 3H, 17-CH_3_), 0.96–0.90 (m, 1H), 0.83 (s, 3H, 20-CH_3_). ^13^C-NMR (CDCl_3_, 75 MHz, ppm): δ 222.81, 178.30, 60.94, 60.46, 55.71, 54.43, 48.72, 48.51, 46.21, 42.36, 40.91, 39.66, 38.55, 38.44, 37.40, 34.38, 27.22, 22.31, 20.43, 20.13, 19.90, 15.48. HRMS (*m*/*z*): calcd for C_22_H_36_NO_3_^+^ [M + H]^+^: 362.2690, found: 362.2987.

### 3.3. General Procedure for the Synthesis of Compound (**2a**–**2e**)

A mixture of isosteviol (63.6 mg, 0.20 mmol), K_2_CO_3_ (41.5 mg, 0.30 mmol) and chlorinated derivatives (0.21 mmol) in CH_3_CN (10 mL) was stirred at 80 °C for 4 h. After confirming the reaction progress by thin-layer chromatography, the solvent was evaporated in vacuo, the mixture was dissolved with 15 mL ethyl acetate and then washed with saline (5 mL × 3). The mixture was then purified using silica gel column chromatography and eluted with petroleum ether:ethyl acetate (5:1) to obtain the target compound **2a**–**2e**.

*2-(4-methylpiperazin-1-yl)-2-oxoethyl(4R,4aS,6aR,9S,11aR,11bS)-4,9,11b-trimethyl-8-oxotetradecahydro-6a,9-methanocyclohepta[a]naphthalene-4-carboxylate* (**2a**). White powder; yield, 87%; m.p. 112–113 °C; ^1^H-NMR (CDCl_3_, 300 MHz, ppm): δ 4.82 (d, *J* = 15 Hz, 1H, -O-CHe-CO-), 4.63 (d, *J* = 15 Hz, 1H, -O-CHa-CO-), 3.65 (s, 2H, -CO-N-CH_2_-), 3.45 (s, 2H, -CO-N-CH_2_-), 2.65 (dd, *J* = 12, 3 Hz, 1H, 15-He), 2.45–2.26 (m, 8H, 3-He, -(CH_2_)_2_-N-CH_3_), 1.97–1.84 (m, 3H), 1.78–1.65 (m, 4H), 1.60–1.55 (m, 2H), 1.50–1.40 (m, 4H), 1.32 (s, 3H, 18-CH_3_), 1.28–1.21 (m, 3H), 1.16–1.07 (m, 1H), 0.99 (s, 3H, 17-CH_3_), 0.95–0.88 (m, 1H), 0.75 (s, 3H, 20-CH_3_). ^13^C-NMR (CDCl_3_, 125 MHz, ppm): δ 222.50, 176.89, 165.02, 60.69, 57.16, 54.77, 54.70, 54.38, 54.33, 50.90, 48.73, 48.51, 45.85, 44.24, 44.08, 41.54, 39.82, 39.48, 38.11, 37.98, 37.33, 29.06, 21.65, 20.34, 19.87, 18.99, 13.57. HRMS (*m*/*z*): calcd for C_27_H_43_N_2_O_4_^+^ [M + H]^+^: 459.3217, found: 459.3221.

*2-(4-ethylpiperazin-1-yl)-2-oxoethyl(4R,4aS,6aR,9S,11aR,11bS)-4,9,11b-trimethyl-8-oxotetradecahydro-6a,9-methanocyclohepta[a]naphthalene-4-carboxylate* (**2b**).White crystal; yield, 84%; m.p. 115–117 °C; ^1^H-NMR (CDCl_3_, 500 MHz, ppm): δ 4.81 (d, *J* = 9 Hz, 1H, -O-CHe-CO-), 4.62 (d, *J* = 9 Hz, 1H, -O-CHa-CO-), 3.64 (s, 2H, -CO-N-CH_2_-), 3.42 (s, 2H, -CO-N-CH_2_-), 2.63 (d, *J* = 12 Hz, 1H, 15-He), 2.45–2.43 (m, 6H, -(CH_2_)_2_-N-CH_2_-), 2.27 (d, *J* = 9 Hz, 1H, 3-He), 1.95–1.92 (m, 1H), 1.87–1.78 (m, 3H), 1.73–1.65 (m, 3H), 1.61 (d, *J* = 9 Hz, 1H), 1.55 (d, *J* = 6 Hz, 1H), 1.51–1.34 (m, 4H), 1.31 (s, 3H, 18-CH_3_), 1.26–1.15 (m, 4H), 1.11–1.06 (m, 3H, -NCH_2_CH_3_), 0.97 (s, 3H, 17-CH_3_), 0.94–0.89 (m, 1H), 0.74 (s, 3H, 20-CH_3_). ^13^C-NMR (CDCl_3_, 75 MHz, ppm): δ 222.57, 176.85, 164.93, 60.72, 57.13, 54.74, 54.30, 52.62, 52.20, 52.13, 48.70, 48.48, 44.40, 44.05, 41.72, 41.51, 39.80, 39.46, 38.08, 37.96, 37.31, 29.04, 21.63, 20.32, 19.85, 18.97, 13.55, 11.77. HRMS (*m*/*z*): calcd for C_28_H_45_N_2_O_4_^+^ [M + H]^+^: 473.3374, found: 473.3379.

*2-oxo-2-(4-phenylpiperazin-1-yl)ethyl(4R,4aS,6aR,9S,11aR,11bS)-4,9,11b-trimethyl-8-oxotetradecahydro-6a,9-methanocyclohepta[a]naphthalene-4-carboxylate* (**2c**). White crystal; yield, 81%; m.p. 143–144 °C; ^1^H-NMR (CDCl_3_, 300 MHz, ppm): δ 7.34–7.30 (m, 2H, Ar-H), 6.96–6.92 (m, 3H, Ar-H), 4.88 (d, *J* = 15 Hz, 1H, -O-CHe-CO-), 4.70 (d, *J* = 15 Hz, 1H, -O-CHa-CO-), 3.80 (s, 2H, -CO-N-CH_2_-), 3.59 (s, 2H, -CO-N-CH_2_-), 3.21 (s, 4H, ph-N-(CH_2_)_2_-), 2.67 (dd, *J* = 18, 3 Hz, 1H, 15-He), 2.30 (d, *J* = 12 Hz, 1H, 3-He), 1.99–1.94 (m, 1H), 1.90–1.77 (m, 3H), 1.74–1.66 (m, 3H), 1.56–1.50 (m, 2H), 1.46–1.39 (m, 2H), 1.34 (s, 3H, 18-CH_3_), 1.27 (s, 2H), 1.23–1.04 (m, 4H), 1.00 (s, 3H, 17-CH_3_), 0.95–0.89 (m, 1H), 0.77 (s, 3H, 20-CH_3_). ^13^C-NMR (CDCl_3_, 75 MHz, ppm): δ 222.44, 176.85, 165.09, 150.82, 129.28(2C), 120.77, 116.80(2C), 60.73, 57.16, 54.77, 54.32, 49.66, 49.39, 48.71, 48.50, 44.56, 44.08, 41.80, 41.53, 39.81, 39.47, 38.11, 37.99, 37.32, 29.07, 21.65, 20.34, 19.87, 18.99, 13.58. HRMS (*m*/*z*): calcd for C_32_H_45_N_2_O_4_^+^ [M + H]^+^: 521.3374, found: 521.3370.

*2-(4-benzylpiperazin-1-yl)-2-oxoethyl(4R,4aS,6aR,9S,11aR,11bS)-4,9,11b-trimethyl-8-oxotetradecahydro-6a,9-methanocyclohepta[a]naphthalene-4-carboxylate* (**2d**). White powder; yield, 83%; m.p. 149–150 °C; ^1^H-NMR (CDCl_3_, 300 MHz, ppm): δ 7.38–7.31 (m, 5H, Ar-H), 4.82 (d, *J* = 15 Hz, 1H, -O-CHe-CO-), 4.63 (d, *J* = 15 Hz, 1H, -O-CHa-CO-), 3.64 (s, 2H, -CO-N-CH_2_-), 3.54 (s, 2H, -N-CH_2_-ph), 3.41 (s, 2H, -CO-N-CH_2_-), 2.66 (dd, *J* = 18, 3 Hz, 1H, 15-He), 2.46 (s, 4H, -N-(CH_2_)_2_-), 2.29 (d, *J* = 12 Hz, 1H, 3-He), 1.97–1.92 (m, 1H), 1.85 (s, 1H), 1.78–1.70 (m, 4H), 1.66–1.59 (m, 3H), 1.55 (s, 1H), 1.50–1.40 (m, 3H), 1.27 (s, 3H, 18-CH_3_), 1.22–1.16 (m, 3H), 1.13–1.07 (m, 1H), 0.99 (s, 3H, 17-CH_3_), 0.94–0.85 (m, 1H), 0.76 (s, 3H, 20-CH_3_). ^13^C-NMR (CDCl_3_, 75 MHz, ppm): δ 222.57, 176.85, 164.94, 137.47, 129.10(2C), 128.37(2C), 127.35, 62.84, 60.74, 57.15, 54.76, 54.32, 52.84, 52.53, 48.72, 48.49, 44.53, 44.06, 41.90, 41.53, 39.81, 39.47, 38.09, 37.97, 37.32, 29.06, 21.63, 20.33, 19.86, 18.99, 13.56. HRMS (*m*/*z*): calcd for C_33_H_47_N_2_O_4_^+^ [M + H]^+^: 535.3530, found: 535.3525.

*2-(azepan-1-yl)-2-oxoethyl(4R,4aS,6aR,9S,11aR,11bS)-4,9,11b-trimethyl-8-oxotetradecahydro-6a,9-methanocyclohepta[a]naphthalene-4-carboxylate* (**2e**). White powder; yield, 83%; m.p. 110–112 °C; ^1^H-NMR (CDCl_3_, 300 MHz, ppm): δ 4.84 (d, *J* = 15 Hz, 1H, -O-CHe-CO-), 4.62 (d, *J* = 15 Hz, 1H, -O-CHa-CO-), 3.55 (t, *J* = 6 Hz, 2H, -CO-N-CH_2_-), 3.39 (t, *J* = 6 Hz, 2H, -CO-N-CH_2_-), 2.67 (dd, *J* = 18, 3 Hz, 1H, 15-He), 2.31 (d, *J* = 12 Hz, 1H, 3-He), 1.99–1.82 (m, 3H), 1.79–1.71 (m, 7H), 1.66–1.59 (m, 7H), 1.55 (s, 1H), 1.50–1.38 (m, 4H), 1.34 (s, 3H, 18-CH_3_), 1.12–1.07 (m, 1H,), 0.99 (s, 3H, 17-CH_3_), 0.94–0.84 (m, 3H), 0.76 (s, 3H, 20-CH_3_). ^13^C-NMR (CDCl_3_, 75 MHz, ppm): δ 222.60, 176.92, 166.05, 60.69, 57.18, 54.76, 54.32, 48.71, 48.50, 46.46, 45.99, 44.02, 41.55, 39.85, 39.47, 38.10, 37.98, 37.33, 29.08, 28.93, 27.47, 27.22, 26.89, 21.64, 20.32, 19.87, 19.01, 13.56. HRMS (*m*/*z*): calcd for C_28_H_44_NO_4_^+^ [M + H]^+^: 458.3265, found: 458.3261.

### 3.4. General Procedure for the Synthesis of Compound (**3a**–**3d**)

A mixture of isosteviol (63.6 mg, 0.20 mmol), EDC∙HCl (42.2 mg, 0.22 mmol), HOBt (29.7 mg, 0.22 mmol), Et_3_N (102 µL, 0.30 mmol) and various amino acid esters (0.30 mmol) in CHCl_3_ (10 mL) was stirred at 60 °C for 8 h. After confirming the reaction progress by thin-layer chromatography, the solvent was evaporated in vacuo, the mixture was dissolved with 15 mL ethyl acetate and then washed with saline (5 mL × 3). The mixture was then purified using silica gel column chromatography and eluted with a gradient of petroleum ether:ethyl acetate (10:1–5:1) to obtain the target compound **3a**–**3d**.

*Methyl(S)-2-phenyl-2-((4R,4aS,6aR,9S,11aR,11bS)-4,9,11b-trimethyl-8-oxotetradecahydro-6a,9-methanocyclohepta[a]naphthalene-4-carboxamido)acetate* (**3a**). White crystal; yield, 70%; m.p. 143–145 °C; ^1^H-NMR (CDCl_3_, 300 MHz, ppm): δ 7.40–7.33 (m, 5H, Ar-H), 6.67 (d, *J* = 9 Hz, 1H, -CO-NH-), 5.56 (d, *J* = 9 Hz, 1H, -NH-CH-ph), 3.75 (s, 3H, -OCH_3_), 2.59 (dd, *J* = 18, 3 Hz, 1H, 15-He), 2.13 (d, *J* = 15 Hz, 1H, 3-He), 1.99–1.85 (m, 2H), 1.81–1.66 (m, 5H), 1.61 (s, 4H), 1.54–1.51 (m, 1H), 1.48–1.39 (m, 2H), 1.24–1.20 (m, 5H), 1.15–1.13 (m, 1H), 0.99 (s, 3H, 17-CH_3_), 0.94–0.87 (m, 1H)_,_ 0.65 (s, 3H, 20-CH_3_). ^13^C-NMR (CDCl_3_, 75 MHz, ppm): δ 222.40, 175.88, 171.76, 136.77, 128.94(2C), 128.47, 127.30(2C), 57.49, 56.36, 54.73, 54.25, 52.77, 48.70, 48.32, 43.70, 41.67, 40.14, 39.47, 38.10, 38.08, 37.28, 29.93, 22.11, 20.30, 19.85, 19.04, 13.40. HRMS (*m*/*z*): calcd for C_29_H_40_NO_4_^+^ [M + H]^+^: 466.2952, found: 466.2947.

*Methyl((4R,4aS,6aR,9S,11aR,11bS)-4,9,11b-trimethyl-8-oxotetradecahydro-6a,9-methanocyclohepta[a]naphthalene-4-carbonyl)-D-phenylalaninate* (**3b**). White crystal; yield, 76%; m.p. 147–148 °C; ^1^H-NMR (CDCl_3_, 300 MHz, ppm): δ 7.33–7.25 (m, 3H, Ar-H), 7.15–7.13 (m, 2H, Ar-H), 5.97 (d, *J* = 6 Hz, 1H, -CO-NH-), 4.84 (dd, *J* = 15, 9 Hz, 1H, -NH-CH-ph), 3.75 (s, 3H, -OCH_3_), 3.19 (dd, *J* = 15, 6 Hz, 1H, ph-CHe-), 3.06 (dd, *J* = 15, 6 Hz, 1H, ph-CHa-), 2.63 (dd, *J* = 18, 3 Hz, 1H, 15-He), 2.05 (d, *J* = 15 Hz, 1H, 3-He), 1.82 (s, 2H), 1.76–1.73 (m, 2H), 1.68–1.64 (m, 5H), 1.59–1.56 (m, 2H), 1.53–1.48 (m, 1H), 1.45–1.33 (m, 4H), 1.27 (s, 3H, 18-CH_3_), 0.98 (s, 3H, 17-CH_3_), 0.92–0.81 (m, 2H)_,_ 0.61 (s, 3H, 20-CH_3_). ^13^C-NMR (CDCl_3_, 75MHz, ppm): δ 222.44, 176.48, 172.43, 136.09, 129.14(2C), 128.65(2C), 127.17, 57.33, 54.71, 54.20, 53.00, 52.29, 48.70, 48.34, 43.74, 41.66, 40.16, 39.49, 38.17, 38.08, 37.82, 37.29, 29.84, 21.88, 20.32, 19.84, 18.89, 13.38. HRMS (*m*/*z*): calcd for C_30_H_42_NO_4_^+^ [M + H]^+^: 480.3108, found: 480.3100.

*Methyl((4R,4aS,6aR,9S,11aR,11bS)-4,9,11b-trimethyl-8-oxotetradecahydro-6a,9-methanocyclohepta[a]naphthalene-4-carbonyl)-L-valinate* (**3c**). White powder; yield, 71%; m.p. 96–98 °C; ^1^H-NMR (CDCl_3_, 300 MHz, ppm): δ 5.99 (d, *J* = 6 Hz, 1H, -CO-NH-), 4.68–4.61 (m, 1H, -NH-CH-), 3.74 (s, 3H, -OCH_3_), 2.66 (dd, *J* = 18, 3 Hz, 1H, 15-He), 2.10–1.96 (m, 2H), 1.89–1.84 (m, 2H), 1.80–1.75 (m, 2H), 1.72–1.58 (m, 7H), 1.55–1.48 (m, 3H), 1.45–1.38 (m, 2H), 1.27 (s, 2H), 1.25–1.20 (m, 5H), 1.18–1.14 (m, 1H), 0.99 (s, 3H, 17-CH_3_), 0.97-0.92 (m, 5H)_,_ 0.77 (s, 3H, 20-CH_3_). ^13^C-NMR (CDCl_3_, 75 MHz, ppm): δ 222.39, 176.41, 173.84, 57.54, 54.68, 54.25, 52.16, 50.36, 48.69, 48.34, 43.76, 41.70, 41.61, 40.17, 39.48, 38.08(2C), 37.28, 30.11, 25.06, 22.85, 22.17, 21.91, 20.31, 19.84, 19.00, 13.53. HRMS (*m*/*z*): calcd for C_27_H_44_NO_4_^+^ [M + H]^+^: 446.3265, found: 446.3260.

*Methyl((4R,4aS,6aR,9S,11aR,11bS)-4,9,11b-trimethyl-8-oxotetradecahydro-6a,9-methanocyclohepta[a]naphthalene-4-carbonyl)-L-tryptophanate* (**3d**). White crystal; yield, 74%; m.p. 165–166 °C; ^1^H-NMR (CDCl_3_, 300 MHz, ppm): δ 8.36 (s, 1H, -NH-), 7.59 (d, *J* = 6 Hz, 1H, Ar-H), 7.40 (d, *J* = 9 Hz, 1H, Ar-H), 7.26–7.12 (m, 2H, Ar-H), 7.05 (s, 1H, -NH-CH=), 6.03 (d, *J* = 6 Hz, 1H, -CO-NH-), 4.85 (q, *J* = 6 Hz, 1H, -NH-CH-), 3.75 (s, 3H, -OCH_3_), 3.38–3.20 (m, 2H, Ar-CH_2_-), 2.40 (dd, *J* = 18, 3 Hz, 1H, 15-He), 1.93 (d, *J* = 15 Hz, 1H, 3-He), 1.75–1.67 (m, 5H), 1.63–1.56 (m, 3H), 1.51–1.43 (m, 2H), 1.37–1.31 (m, 5H), 1.28 (s, 3H, 18-CH_3_), 1.10–1.08 (m, 1H), 0.97 (s, 3H, 17-CH_3_), 0.92–0.77 (m, 2H), 0.52 (s, 3H, 20-CH_3_). ^13^C-NMR (CDCl_3_, 75 MHz, ppm): δ 222.60, 176.60, 172.94, 136.12, 127.60, 122.48(2C), 119.84, 118.50, 111.43, 110.41, 57.35, 54.62, 54.18, 53.15, 52.29, 48.69, 48.26, 43.53, 41.61, 40.07, 39.32, 37.96, 37.92, 37.24, 29.85, 27.23, 21.80, 20.24, 19.83, 18.84, 13.08. HRMS (*m*/*z*): calcd for C_32_H_43_N_2_O_4_^+^ [M + H]^+^: 519.3217, found: 519.3221.

### 3.5. General Procedure for the Synthesis of Compound (**4a**–**4j**)

A mixture of isosteviol (63.6 mg, 0.20 mmol), K_2_CO_3_ (41.5 mg, 0.30 mmol) and different chloroacetanilides (0.21 mmol) in CH_3_CN (10 mL) was stirred at 80 °C for 2 h. After confirming the reaction progress by thin-layer chromatography, the solvent was evaporated in vacuo, the mixture was dissolved with 15 mL ethyl acetate and then washed with saline (5 mL × 3). The mixture was then purified using silica gel column chromatography and eluted with a gradient of petroleum ether:ethyl acetate (4:1–5:1) to obtain the target compound **4a**–**4j**.

*2-oxo-2-(phenylamino)ethyl(4R,4aS,6aR,9S,11aR,11bS)-4,9,11b-trimethyl-8-oxotetradecahydro-6a,9-methanocyclohepta[a]naphthalene-4-carboxylate* (**4a**). White powder; yield, 86%; m.p. 154–156 °C; ^1^H-NMR (CDCl_3_, 300 MHz, ppm): δ 7.92 (s, 1H, -CO-NH-), 7.51 (d, *J* = 6 Hz, 2H, Ar-H), 7.35 (t, *J* = 6 Hz, 2H, Ar-H), 7.15 (t, *J* = 6 Hz, 1H, Ar-H), 4.75 (d, *J* = 15 Hz, 1H, -O-CHe-CO-), 4.58 (d, *J* = 15 Hz, 1H, -O-CHa-CO-), 2.62 (dd, *J* = 18, 3 Hz, 1H, 15-He), 2.28 (d, *J* = 12 Hz, 1H, 3-He), 2.02 (d, *J* = 15 Hz, 1H, 6-He), 1.84 (s, 1H), 1.79–1.70 (m, 5H), 1.64–1.54 (m, 4H), 1.51–1.40 (m, 3H), 1.34 (s, 3H, 18-CH_3_), 1.23–1.20 (m, 3H), 1.17–1.11 (m, 1H), 0.99 (s, 3H, 17-CH_3_), 0.74 (s, 3H, 20-CH_3_). ^13^C-NMR (CDCl_3_, 75 MHz, ppm): δ 221.95, 175.76, 165.03, 136.83, 129.22(2C), 124.94, 119.74(2C), 63.36, 57.00, 54.71, 54.19, 48.68, 48.39, 44.03, 41.39, 39.61, 39.43, 38.06, 37.99, 37.20, 28.99, 22.01, 20.34, 19.83, 18.93, 13.50. HRMS (*m*/*z*): calcd for C_28_H_38_NO_4_^+^ [M + H]^+^: 452.2795, found: 452.2790.

*2-((2-chlorophenyl)amino)-2-oxoethyl(4R,4aS,6aR,9S,11aR,11bS)-4,9,11b-trimethyl-8-oxotetradecahydro-6a,9-methanocyclohepta[a]naphthalene-4-carboxylate* (**4b**). White powder; yield, 83%; m.p. 107–108 °C; ^1^H-NMR (CDCl_3_, 500 MHz, ppm): δ 8.46–8.43 (m, 1H, -CO-NH-), 7.39 (d, *J* = 10 Hz, 1H, Ar-H), 7.33–7.29 (m, 2H, Ar-H), 7.09 (t, *J* = 10 Hz, 1H, Ar-H), 4.79 (d, *J* = 15 Hz, 1H, -O-CHe-CO-), 4.62 (d, *J* = 15 Hz, 1H, -O-CHa-CO-), 2.61 (dd, *J* = 20, 5 Hz, 1H, 15-He), 2.31 (d, *J* = 15 Hz, 1H, 3-He), 2.02 (d, *J* = 15 Hz, 1H, 6-He), 1.91–1.85 (m, 1H), 1.82–1.68 (m, 4H), 1.63–1.60 (m, 3H), 1.55–1.49 (m, 4H), 1.44–1.40 (m,1H), 1.35 (s, 3H, 18-CH_3_), 1.27–1.22 (m, 3H), 1.20–1.13 (m, 1H), 0.98 (s, 3H, 17-CH_3_), 0.73 (s, 3H, 20-CH_3_). ^13^C-NMR (CDCl_3_, 75 MHz, ppm): δ 222.03, 175.62, 165.27, 133.74, 129.12, 127.95, 125.25, 122.65, 121.67, 63.41, 57.10, 54.73, 54.23, 48.67, 48.39, 44.06, 41.31, 39.59, 39.40, 38.02, 37.98, 37.22, 29.01, 21.97, 20.33, 19.83, 19.08, 13.43. HRMS (*m*/*z*): calcd for C_28_H_37_ClNO_4_^+^ [M + H]^+^: 486.2406, found: 486.2401.

*2-((3-chlorophenyl)amino)-2-oxoethyl(4R,4aS,6aR,9S,11aR,11bS)-4,9,11b-trimethyl-8-oxotetradecahydro-6a,9-methanocyclohepta[a]naphthalene-4-carboxylate* (**4c**). White powder; yield, 80%; m.p. 120–122 °C; ^1^H-NMR (CDCl_3_, 500 MHz, ppm): δ 7.80 (s, 1H, -CO-NH-), 7.60 (s, 1H, Ar-H), 7.36 (d, *J* = 5 Hz, 1H, Ar-H), 7.31–7.28 (m, 1H, Ar-H), 7.14 (t, *J* = 5 Hz, 1H, Ar-H), 4.74 (d, *J* = 15 Hz, 1H, -O-CHe-CO-), 4.57 (d, *J* = 15 Hz, 1H, -O-CHa-CO-), 2.61 (dd, *J* = 20, 5 Hz, 1H, 15-He), 2.27 (d, *J* = 15 Hz, 1H, 3-He), 2.00 (d, *J* = 10 Hz, 1H, 6-He), 1.87–1.71 (m, 5H), 1.63–1.53 (m, 5H), 1.45–1.38 (m, 2H), 1.33 (s, 3H, 18-CH_3_), 1.26–1.21 (m, 3H), 1.20–1.13 (m, 1H), 0.98 (s, 3H, 17-CH_3_), 0.95–0.84 (m, 1H), 0.73 (s, 3H, 20-CH_3_). ^13^C-NMR (CDCl_3_, 75 MHz, ppm): δ 222.02, 175.84, 165.17, 137.98, 134.86, 130.21, 124.98, 119.83, 117.67, 63.28, 56.99, 54.70, 54.18, 48.69, 48.40, 44.04, 41.36, 39.59, 39.43, 38.06, 37.97, 37.20, 28.99, 21.99, 20.33, 19.81, 18.92, 13.50. HRMS (*m*/*z*): calcd for C_28_H_37_ClNO_4_^+^ [M + H]^+^: 486.2406, found: 486.2403.

*2-((4-chlorophenyl)amino)-2-oxoethyl(4R,4aS,6aR,9S,11aR,11bS)-4,9,11b-trimethyl-8-oxotetradecahydro-6a,9-methanocyclohepta[a]naphthalene-4-carboxylate* (**4d**). White powder; yield, 84%; m.p. 140–142 °C; ^1^H-NMR (CDCl_3_, 500 MHz, ppm): δ 7.80 (s, 1H, -CO-NH-), 7.46 (d, *J* = 5 Hz, 2H, Ar-H), 7.32-7.30 (m, 2H, Ar-H), 4.73 (d, *J* = 15 Hz, 1H, -O-CHe-CO-), 4.57 (d, *J* = 15 Hz, 1H, -O-CHa-CO-), 2.61 (dd, *J* = 20, 5 Hz, 1H, 18-He), 2.27 (d, *J* = 15 Hz, 1H, 3-He), 2.00 (d, *J* = 15 Hz, 1H, 6-He), 1.84–1.69 (m, 6H), 1.63–1.62 (m, 2H), 1.56–1.52 (m, 2H), 1.49–1.38 (m, 2H), 1.32 (s, 3H, 18-CH_3_), 1.26–1.23 (m, 3H), 1.20–1.13 (m, 1H), 0.98–0.88 (m, 4H), 0.73 (s, 3H, 20-CH_3_). ^13^C-NMR (CDCl_3_, 75 MHz, ppm): δ 221.95, 175.84, 165.08, 135.42, 129.95, 129.25(2C), 120.94(2C), 63.33, 56.99, 54.69, 54.18, 48.69, 48.39, 44.03, 41.37, 39.58, 39.42, 38.06, 37.96, 37.19, 28.99, 21.97, 20.33, 19.82, 18.92, 13.51. HRMS (*m*/*z*): calcd for C_28_H_37_ClNO_4_^+^ [M + H]^+^: 486.2406, found: 486.2401.

*2-((2-fluorophenyl)amino)-2-oxoethyl(4R,4aS,6aR,9S,11aR,11bS)-4,9,11b-trimethyl-8-oxotetradecahydro-6a,9-methanocyclohepta[a]naphthalene-4-carboxylate* (**4e**). White crystal; yield, 81%; m.p. 168–169 °C; ^1^H-NMR (CDCl_3_, 500 MHz, ppm): δ 8.42–8.39 (m, 1H, Ar-H), 8.18 (s, 1H, -CO-NH-), 7.18–7.10 (m, 3H, Ar-H), 4.78 (d, *J* = 15 Hz, 1H, -O-CHe-CO-), 4.60 (d, *J* = 15 Hz, 1H, -O-CHa-CO-), 2.61 (d, *J* = 20 Hz, 1H, 15-He), 2.28 (d, *J* = 15 Hz, 1H, 3-He), 2.03 (d, *J* = 10 Hz, 1H, 6-He), 1.89–1.71 (m, 5H), 1.63–1.59 (m, 2H), 1.54–1.51 (m, 2H), 1.44–1.38 (m, 2H), 1.33 (s, 3H, 18-CH_3_), 1.26–1.23 (m, 4H), 1.18–1.13 (m, 1H), 0.99 (s, 3H, 17-CH_3_), 0.94–0.84 (m, 1H), 0.74 (s, 3H, 20-CH_3_). ^13^C-NMR (CDCl_3_, 75 MHz, ppm): δ 222.03, 175.64, 165.16, 125.58, 124.92, 124.84, 121.43, 114.95, 114.70, 63.28, 56.95, 54.74, 54.21, 48.68, 48.36, 44.05, 41.35, 39.64, 39.43, 38.05(2C), 37.21, 28.04, 22.00, 20.34, 19.83, 18.84, 13.40. HRMS (*m*/*z*): calcd for C_28_H_37_FNO_4_^+^ [M + H]^+^: 470.2701, found: 470.2704.

*2-((3-fluorophenyl)amino)-2-oxoethyl(4R,4aS,6aR,9S,11aR,11bS)-4,9,11b-trimethyl-8-oxotetradecahydro-6a,9-methanocyclohepta[a]naphthalene-4-carboxylate* (**4f**). White powder; yield, 80%; m.p. 144–145 °C; ^1^H-NMR (CDCl_3_, 500 MHz, ppm): δ 7.84 (s, 1H, -CO-NH-), 7.47 (d, *J* = 10 Hz, 1H, Ar-H), 7.31–7.28 (m, 1H, Ar-H), 7.13 (d, *J* = 5 Hz, 1H, Ar-H), 6.88–6.84 (m, 1H, Ar-H), 4.74 (d, *J* = 15 Hz, 1H, -O-CHe-CO-), 4.58 (d, *J* = 15 Hz, 1H, -O-CHa-CO-), 2.61 (dd, *J* = 20, 5 Hz, 1H, 18-He), 2.27 (d, *J* = 15 Hz, 1H, 3-He), 2.01 (d, *J* = 15 Hz, 1H, 6-He), 1.87–1.69 (m, 5H), 1.63–1.61 (m, 1H), 1.54-1.52 (m, 5H), 1.43 (dd, *J* = 9, 3 Hz, 1H), 1.40–1.35 (m, 1H), 1.33 (s, 2H), 1.27–1.20 (m, 3H), 1.19–1.13 (m, 1H), 0.98 (s, 3H, 17-CH_3_), 0.95–0.86 (m, 1H), 0.73 (s, 3H, 20-CH_3_). ^13^C-NMR (CDCl_3_, 75 MHz, ppm): δ 221.98, 175.81, 165.14, 164.65, 138.32, 130.31, 114.84, 111.67, 107.31, 63.30, 56.98, 54.69, 54.17, 48.69, 48.39, 44.03, 41.36, 39.58, 39.42, 38.06, 37.96, 37.19, 28.98, 21.99, 20.32, 19.81, 18.92, 13.50. HRMS (*m*/*z*): calcd for C_28_H_37_FNO_4_^+^ [M + H]^+^: 470.2701, found: 470.2699.

*2-((4-fluorophenyl)amino)-2-oxoethyl(4R,4aS,6aR,9S,11aR,11bS)-4,9,11b-trimethyl-8-oxotetradecahydro-6a,9-methanocyclohepta[a]naphthalene-4-carboxylate* (**4g**). White powder; yield, 90%; m.p. 140–141 °C; ^1^H-NMR (CDCl_3_, 500 MHz, ppm): δ 7.76 (s, 1H, -CO-NH-), 7.48–7.45 (m, 2H, Ar-H), 7.05 (t, *J* = 10 Hz, 2H, Ar-H), 4.74 (d, *J* = 15 Hz, 1H, -O-CHe-CO-), 4.58 (d, *J* = 15 Hz, 1H, -O-CHa-CO-), 2.61 (d, *J* = 20 Hz, 1H, 18-He), 2.27 (d, *J* = 15 Hz, 1H, 3-He), 2.00 (d, *J* = 15 Hz, 1H, 6-He), 1.87–1.70 (m, 5H), 1.63–1.61 (m, 2H), 1.55–1.52 (m, 3H), 1.44–1.36 (m, 2H), 1.33 (s, 3H), 1.26–1.23 (m, 3H), 1.19–1.13 (m, 1H), 0.98 (s, 3H), 0.95–0.86 (m, 1H), 0.73 (s, 3H, 20-CH_3_). ^13^C-NMR (CDCl_3_, 75 MHz, ppm): δ 222.01, 175.86, 165.06, 161.29, 132.87, 121.63, 121.53, 116.06, 115.75, 63.29, 56.99, 54.69, 54.18, 48.69, 48.40, 44.02, 41.37, 39.59, 39.42, 38.06, 37.96, 37.19, 28.99, 21.96, 20.32, 19.82, 18.92, 13.51. HRMS (*m*/*z*): calcd for C_28_H_37_FNO_4_^+^ [M + H]^+^: 470.2701, found: 470.2707.

*2-((4-nitrophenyl)amino)-2-oxoethyl(4R,4aS,6aR,9S,11aR,11bS)-4,9,11b-trimethyl-8-oxotetradecahydro-6a,9-methanocyclohepta[a]naphthalene-4-carboxylate* (**4h**). Pale yellow solid; yield, 78%; m.p. 189–190 °C; ^1^H-NMR (CDCl_3_, 500 MHz, ppm): δ 8.24 (d, *J* = 10 Hz, 2H, Ar-H), 8.08 (s, 1H, -CO-NH-), 7.69 (d, *J* = 10 Hz, 2H, Ar-H), 4.77 (d, *J* = 15 Hz, 1H, -O-CHe-CO-), 4.62 (d, *J* = 15 Hz, 1H, -O-CHa-CO-), 2.60 (d, *J* = 20 Hz, 1H, 18-He), 2.27 (d, *J* = 10 Hz, 1H, 3-He), 2.00 (d, *J* = 15 Hz, 1H, 6-He), 1.83–1.76 (m, 3H), 1.74–1.70 (m, 2H), 1.63–1.59 (m, 2H), 1.45–1.37 (m, 3H), 1.34 (s, 3H), 1.30 (s, 1H), 1.23-1.10 (m, 4H), 0.98 (s, 3H), 0.95–0.84 (m, 2H), 0.73 (s, 3H, 20-CH_3_). ^13^C-NMR (CDCl_3_, 125 MHz, ppm): δ 222.00, 175.99, 165.48, 144.11, 142.50, 125.27(2C), 119.17(2C), 63.42, 56.99, 54.69, 54.18, 48.70, 48.41, 44.10, 41.36, 39.55, 39.44, 38.08, 37.97, 37.19, 29.01, 21.98, 20.34, 19.81, 18.94, 13.55. HRMS (*m*/*z*): calcd for C_28_H_37_N_2_O_6_^+^ [M + H]^+^: 497.2646, found: 497.2640.

*2-((4-methoxyphenyl)amino)-2-oxoethyl(4R,4aS,6aR,9S,11aR,11bS)-4,9,11b-trimethyl-8-oxotetradecahydro-6a,9-methanocyclohepta[a]naphthalene-4-carboxylate* (**4i**). White powder; yield, 81%; m.p. 86–88 °C; ^1^H-NMR (CDCl_3_, 500 MHz, ppm): δ 7.70 (s, 1H, -CO-NH-), 7.40 (d, *J* = 15 Hz, 2H, Ar-H), 6.88 (d, *J* = 10 Hz, 2H, Ar-H), 4.73 (d, *J* = 15 Hz, 1H, -O-CHe-CO-), 4.57 (d, *J* = 15 Hz, 1H, -O-CHa-CO-), 3.80 (s, 3H, ph-O-CH_3_), 2.62 (d, *J* = 15 Hz, 1H, 18-He), 2.27 (d, *J* = 15 Hz, 1H, 3-He), 2.00 (d, *J* = 10 Hz, 1H, 6-He), 1.88–1.70 (m, 5H), 1.63–1.58 (m, 3H), 1.56–1.52 (m, 2H), 1.44–1.39 (m, 2H), 1.32 (s, 3H, 18-CH_3_), 1.25–1.23 (m, 3H), 1.18–1.12 (m, 1H), 0.98 (s, 3H), 0.94–0.88 (m, 1H), 0.74 (s, 3H, 20-CH_3_). ^13^C-NMR (CDCl_3_, 75 MHz, ppm): δ 222.01, 175.81, 164.88, 156.85, 129.90, 121.60(2C), 114.35(2C), 63.35, 57.01, 55.52, 54.71, 54.20, 48.70, 48.39, 44.02, 41.39, 39.61, 39.42, 38.05, 37.98, 37.20, 28.99, 21.98, 20.33, 19.81, 18.93, 13.50. HRMS (*m*/*z*): calcd for C_29_H_40_NO_5_^+^ [M + H]^+^: 482.2901, found: 482.2905.

*2-((2,5-dimethoxyphenyl)amino)-2-oxoethyl(4R,4aS,6aR,9S,11aR,11bS)-4,9,11b-trimethyl-8-oxotetradecahydro-6a,9-methanocyclohepta[a]naphthalene-4-carboxylate* (**4j**). White powder; yield, 79%; m.p. 166–168 °C; ^1^H-NMR (CDCl_3_, 500 MHz, ppm): δ 8.55 (s, 1H, -CO-NH-), 8.14 (d, *J* = 3 Hz, 1H, Ar-H), 6.81 (d, *J* = 5 Hz, 1H, Ar-H), 6.61 (dd, *J* = 10, 5 Hz, 1H, Ar-H), 4.80 (d, *J* = 15 Hz, 1H, -O-CHe-CO-), 4.54 (d, *J* = 15 Hz, 1H, -O-CHa-CO-), 3.83 (s, 3H, ph-O-CH_3_), 3.79 (s, 3H, ph-O-CH_3_), 2.61 (dd, *J* = 15, 5 Hz, 1H, 18-He), 2.31 (d, *J* = 15 Hz, 1H, 3-He), 2.01 (d, *J* = 15 Hz, 1H, 6-He), 1.88 (qt, *J* = 9, 3Hz, 1H) 1.82–1.74 (m, 3H), 1.71–1.67 (m, 2H), 1.63–1.60 (m, 1H), 1.55–1.47 (m, 3H), 1.44–1.37 (m, 2H), 1.35 (s, 3H, 18-CH_3_), 1.27–1.22 (m, 3H), 1.18–1.10 (m, 1H), 0.98–0.93 (m, 4H), 0.73 (s, 3H, 20-CH_3_). ^13^C-NMR (CDCl_3_, 75 MHz, ppm): δ 222.00, 175.50, 164.97, 153.94, 142.05, 127.36, 110.68, 109.07, 106.08, 63.14, 57.09, 56.04, 55.83, 54.74, 54.24, 48.66, 48.39, 43.99, 41.39, 39.63, 39.41, 38.02, 37.88, 37.22, 28.83, 21.76, 20.32, 19.83, 18.96, 13.38. HRMS (*m*/*z*): calcd for C_30_H_42_NO_6_^+^ [M + H]^+^: 512.3007, found: 512.3001.

### 3.6. General Procedure for the Synthesis of Compound (**5a**–**5e**)

A mixture of isosteviol (63.6 mg, 0.20 mmol), K_2_CO_3_ (41.5 mg, 0.30 mmol) and different phenyl 1,2,3-triazole chloroacetamides (0.21 mmol) in CH_3_CN (10 mL) was stirred at 80 °C for 2 h. After confirming the reaction progress by thin-layer chromatography, the solvent was evaporated in vacuo, the mixture was dissolved with 15 mL ethyl acetate and then washed with saline (5 mL × 3). The mixture was then purified using silica gel column chromatography and eluted with petroleum ether:ethyl acetate (1:1) to obtain the target compound **5a**–**5e**.

*2-oxo-2-(((1-phenyl-1H-1,2,3-triazol-4-yl)methyl)amino)ethyl(4R,4aS,6aR,9S,11aR,11bS)-4,9,11b-trimethyl-8-oxotetradecahydro-6a,9-methanocyclohepta[a]naphthalene-4-carboxylate* (**5a**). White solid; yield, 81%; m.p. 83–84 °C; ^1^H-NMR (CDCl_3_, 300 MHz, ppm): δ 8.01 (s, 1H, triazole-H), 7.74-7.71 (m, 2H, Ar-H), 7.56–7.42 (m, 3H, Ar-H), 6.91 (brs, 1H, -NH-CO-), 4.74–4.46 (m, 4H, -O-CH_2_-, -CH_2_-NH-), 2.56 (dd, *J* = 18, 3 Hz, 1H, 15-He), 2.22 (d, *J* = 12 Hz, 1H, 3-He), 1.95 (d, *J* = 12 Hz, 1H, 6-He), 1.85–1.66 (m, 6H), 1.62–1.47 (m, 4H), 1.42–1.37 (m, 2H), 1.27 (s, 3H, 18-CH_3_), 1.20–1.13 (m, 3H), 1.10–1.04 (m, 1H), 0.96 (s, 3H, 17-CH_3_), 0.93–0.88 (m, 1H), 0.67 (s, 3H, 20-CH_3_). ^13^C-NMR (CDCl_3_, 75 MHz, ppm): δ 222.09, 176.00, 167.37, 144.64, 136.87, 129.81(2C), 128.93, 120.52(2C), 120.44, 62.98, 56.98, 54.66, 54.17, 48.64, 48.31, 43.95, 41.28, 39.61, 39.39, 37.99, 37.94, 37.20, 34.69, 28.91, 21.90, 20.29, 19.82, 18.87, 13.42. HRMS (*m*/*z*): calcd for C_31_H_41_N_4_O_4_^+^ [M + H]^+^: 533.3122, found: 533.3119.

*2-(((1-(4-chlorophenyl)-1H-1,2,3-triazol-4-yl)methyl)amino)-2-oxoethyl(4R,4aS,6aR,9S,11aR,11bS)-4,9,11b-trimethyl-8-oxotetradecahydro-6a,9-methanocyclohepta[a]naphthalene-4-carboxylate* (**5b**). White solid; yield, 82%; m.p. 80–82 °C; ^1^H-NMR (CDCl_3_, 300 MHz, ppm): δ 8.00 (s, 1H, triazole-H), 7.71–7.68 (m, 2H, Ar-H), 7.54–7.51 (m, 2H, Ar-H), 6.81 (brs, 1H, -NH-CO-), 4.74–4.47 (m, 4H, -O-CH_2_-, -CH_2_-NH-), 2.57 (dd, *J* = 18, 3 Hz, 1H, 15-He), 2.23 (d, *J* = 12 Hz, 1H, 3-He), 1.96 (d, *J* = 12 Hz, 1H, 6-He), 1.82–1.68 (m, 6H), 1.60–1.39 (m, 6H), 1.28 (s, 3H, 18-CH_3_), 1.22–1.15 (m, 3H), 1.12–1.06 (m, 1H), 0.99 (s, 3H, 17-CH_3_), 0.95–0.87 (m, 1H), 0.68 (s, 3H, 20-CH_3_). ^13^C-NMR (CDCl_3_, 75 MHz, ppm): δ 222.09, 176.02, 167.44, 144.90, 135.35, 134.76, 130.01(2C), 121.69(2C), 120.44, 62.99, 56.98, 54.67, 54.18, 48.66, 48.33, 43.96, 41.30, 39.61, 39.40, 38.00, 37.94, 37.21, 34.64, 28.91, 21.91, 20.30, 19.82, 18.87, 13.42. HRMS (*m*/*z*): calcd for C_31_H_40_ClN_4_O_4_^+^ [M + H]^+^: 567.2733, found: 567.2729.

*2-(((1-(4-methoxyphenyl)-1H-1,2,3-triazol-4-yl)methyl)amino)-2-oxoethyl(4R,4aS,6aR,9S,11aR,11bS)-4,9,11b-trimethyl-8-oxotetradecahydro-6a,9-methanocyclohepta[a]naphthalene-4-carboxylate* (**5c**). White solid; yield, 83%; m.p. 71–72 °C; ^1^H-NMR (CDCl_3_, 300 MHz, ppm): δ 7.92 (s, 1H, triazole-H), 7.62 (d, *J* = 6 Hz, 2H, Ar-H), 7.04 (d, *J* = 6 Hz, 2H, Ar-H), 6.87 (brs, 1H, -NH-CO-), 4.74–4.47 (m, 4H, -O-CH_2_-, -CH_2_-NH-), 3.88 (s, 3H, -O-CH_3_), 2.57 (dd, *J* = 18, 3 Hz, 1H, 15-He), 2.23 (d, *J* = 12 Hz, 1H, 3-He), 1.96 (d, *J* = 12 Hz, 1H, 6-He), 1.80–1.68 (m, 6H), 1.62–1.48 (m, 4H), 1.45–1.38 (m, 3H), 1.23–1.19 (m, 3H), 1.16–1.05 (m, 2H), 0.98 (s, 3H, 17-CH_3_), 0.94–0.87 (m, 2H), 0.68 (s, 3H, 20-CH_3_). ^13^C-NMR (CDCl_3_, 75 MHz, ppm): δ 222.11, 175.97, 167.33, 159.95, 144.37, 130.30, 122.18(2C), 120.55, 114.84(2C), 63.00, 56.98, 55.64, 54.67, 54.17, 48.65, 48.30, 43.95, 41.28, 39.61, 39.39, 38.00, 37.94, 37.21, 34.70, 28.91, 21.92, 20.29, 19.82, 18.87, 13.41. HRMS (*m*/*z*): calcd for C_32_H_43_N_4_O_5_^+^ [M + H]^+^: 563.3228, found: 563.3225.

*2-oxo-2-(((1-(p-tolyl)-1H-1,2,3-triazol-4-yl)methyl)amino)ethyl(4R,4aS,6aR,9S,11aR,11bS)-4,9,11b-trimethyl-8-oxotetradecahydro-6a,9-methanocyclohepta[a]naphthalene-4-carboxylate* (**5d**). White solid; yield, 83%; m.p. 66–68 °C; ^1^H-NMR (CDCl_3_, 300 MHz, ppm): δ 7.96 (s, 1H, triazole-H), 7.60 (d, *J* = 9 Hz, 2H, Ar-H), 7.33 (d, *J* = 9 Hz, 2H, Ar-H), 6.84 (brs, 1H, -NH-CO-), 4.74–4.47 (m, 4H, -O-CH_2_-, -CH_2_-NH-), 2.56 (dd, *J* = 21, 3 Hz, 1H, 15-He), 2.43 (s, 3H, Ar-CH_3_), 2.23 (d, *J* = 15 Hz, 1H, 3-He), 1.95 (d, *J* = 12 Hz, 1H, 6-He), 1.80–1.66 (m, 6H), 1.62–1.48 (m, 4H), 1.43–1.38 (m, 2H), 1.28 (s, 3H, 18-CH_3_), 1.21–1.15 (m, 3H), 1.10–1.05 (m, 1H), 0.97 (s, 3H, 17-CH_3_), 0.93–0.88 (m, 1H), 0.68 (s, 3H, 20-CH_3_). ^13^C-NMR (CDCl_3_, 75 MHz, ppm): δ 222.15, 175.97, 167.34, 144.42, 139.10, 134.58, 130.31(2C), 120.44(2C), 120.41, 62.99, 56.97, 54.66, 54.16, 48.65, 48.30, 43.94, 41.27, 39.60, 39.38, 37.99, 37.94, 37.20, 34.71, 28.92, 21.91, 21.11, 20.29, 19.83, 18.87, 13.41. HRMS (*m*/*z*): calcd for C_32_H_43_N_4_O_4_^+^ [M + H]^+^: 547.3279, found: 547.3276.

*2-(((1-(3,4-dichlorophenyl)-1H-1,2,3-triazol-4-yl)methyl)amino)-2-oxoethyl(4R,4aS,6aR,9S,11aR,11bS)-4,9,11b-trimethyl-8-oxotetradecahydro-6a,9-methanocyclohepta[a]naphthalene-4-carboxylate* (**5e**). White solid; yield, 79%; m.p. 96–97 °C; ^1^H-NMR (CDCl_3_, 300 MHz, ppm): δ 8.02 (s, 1H, triazole-H), 7.92–7.91 (m, 1H, Ar-H), 7.65–7.59 (m, 2H, Ar-H), 6.81 (brs, 1H, -NH-CO-), 4.73–4.47 (m, 4H, -O-CH_2_-, -CH_2_-NH-), 2.56 (dd, *J* = 18, 3 Hz, 1H, 15-He), 2.23 (d, *J* = 15 Hz, 1H, 3-He), 1.98–1.93 (m, 1H, 6-He), 1.82-1.67 (m, 6H), 1.63–1.47 (m, 4H), 1.45–1.35 (m, 2H), 1.28 (s, 3H, 18-CH_3_), 1.22–1.15 (m, 3H), 1.12–1.06 (m, 1H), 0.98 (s, 3H, 17-CH_3_), 0.95–0.89 (m, 1H), 0.69 (s, 3H, 20-CH_3_). ^13^C-NMR (CDCl_3_, 75 MHz, ppm): δ 222.08, 176.06, 167.50, 145.20, 135.87, 134.08, 133.07, 131.55, 122.26, 120.43, 119.39, 63.00, 56.98, 54.66, 54.19, 48.66, 48.34, 43.96, 41.31, 39.60, 39.40, 38.01, 37.94, 37.21, 34.64, 28.92, 21.89, 20.31, 19.82, 18.88, 13.44. HRMS (*m*/*z*): calcd for C_31_H_39_Cl_2_N_4_O_4_^+^ [M + H]^+^: 601.2343, found: 601.2340.

### 3.7. General Procedure for the Synthesis of Compound (**6a**–**6e**)

A mixture of isosteviol (63.6 mg, 0.20 mmol), K_2_CO_3_ (41.5 mg, 0.30 mmol) and different phosphonic acid [[(chloroacetyl) amino] phenylmethyl]-diethyl esters (0.21 mmol) in CH_3_CN (10 mL) was stirred at 80 °C for 3 h. After confirming the reaction progress by thin-layer chromatography, the solvent was evaporated in vacuo, the mixture was dissolved with 15 mL ethyl acetate and then washed with saline (5 mL ×3). The mixture was then purified using silica gel column chromatography and eluted with a gradient of petroleum ether:ethyl acetate (3:1-1:1) to obtain the target compound **6a**–**6e**.

*2-(((diethoxyphosphoryl)(phenyl)methyl)amino)-2-oxoethyl(4R,4aS,6aR,9S,11aR,11bS)-4,9,11b-trimethyl-8-oxotetradecahydro-6a,9-methanocyclohepta[a]naphthalene-4-carboxylate* (**6a**). White powder; yield, 81%; m.p. 156–157 °C; ^1^H-NMR (CDCl_3_, 300 MHz, ppm): δ 7.41–7.35 (m, 5H, Ar-H), 7.20–7.12 (m, 1H, -NH-CO-), 5.48 (dd, *J* = 18, 9 Hz, 1H, -P-CH-NH-), 4.76–4.64 (m, 1H, -O-CHe-CO-), 4.53–4.40 (m, 1H, -O-CHa-CO-), 4.19–4.07 (m, 2H, -O-CHe-), 4.00–3.87 (m, 1H, -O-CHa-), 3.77–3.62 (m, 1H, -O-CHa-), 2.65–2.56 (m, 1H, 15-He), 2.27 (d, *J* = 15 Hz, 1H, 3-He), 2.09–1.99 (m, 1H, 6-He), 1.89–1.69 (m, 6H), 1.65–1.50 (m, 4H), 1.45–1.41 (m, 2H), 1.35–1.29 (m, 6H), 1.27–1.19 (m, 3H), 1.15–1.07 (m, 4H), 1.00–0.99 (m, 3H), 0.95–0.87 (m, 1H), 0.70 (s, 3H, 20-CH_3_). ^13^C-NMR (CDCl_3_, 75 MHz, ppm): δ 221.94, 175.72, 166.43, 134.77, 128.78(2C), 128.38, 127.91(2C), 63.60, 63.12, 62.74, 56.91, 54.73, 54.21, 50.82, 48.68, 48.40, 43.97, 41.41, 39.64, 39.41, 38.02(2C), 37.23, 28.86, 21.92, 20.32, 19.82, 18.93, 16.42, 16.10, 13.38. HRMS (*m*/*z*): calcd for C_33_H_49_NO_7_P^+^ [M + H]^+^: 602.3241, found: 602.3238.

*2-(((4-chlorophenyl)(diethoxyphosphoryl)methyl)amino)-2-oxoethyl(4R,4aS,6aR,9S,11aR,11bS)-4,9,11b-trimethyl-8-oxotetradecahydro-6a,9-methanocyclohepta[a]naphthalene-4-carboxylate* (**6b**). White powder; yield, 83%; m.p. 149–150 °C; ^1^H-NMR (CDCl_3_, 300 MHz, ppm): δ 7.46–7.33 (m, 4H, Ar-H), 7.27–7.16 (m, 1H, -NH-CO-), 5.44 (ddd, *J* = 21, 9, 3 Hz, 1H, -P-CH-NH-), 4.68 (t, *J* = 15 Hz, 1H, -O-CHe-CO-), 4.46 (t, *J* = 15 Hz, 1H, -O-CHa-CO-), 4.18–4.09 (m, 2H, -O-CHe-), 4.03–3.90 (m, 1H, -O-CHa-), 3.84–3.70 (m, 1H, -O-CHa-), 2.60 (d, *J* = 18 Hz, 1H, 15-He), 2.25 (d, *J* = 12 Hz, 1H, 3-He), 2.05–1.98 (m, 1H, 6-He), 1.89–1.65 (m, 7H), 1.60–1.53 (m, 3H), 1.45–1.40 (m, 2H), 1.35–1.29 (m, 6H), 1.26–1.22 (m, 4H), 1.16–1.11 (m, 3H), 1.00–0.99 (m, 3H), 0.95–0.91 (m, 1H), 0.70–0.69 (m, 3H, 20-CH_3_). ^13^C-NMR (CDCl_3_, 75 MHz, ppm): δ 221.99, 175.77, 166.59, 134.39, 133.40, 129.30, 129.19, 128.99(2C), 63.72, 63.30, 62.78, 56.96, 54.72, 54.21, 50.31, 48.68, 48.41, 43.97, 41.36, 39.64, 39.41, 38.02, 37.98, 37.22, 28.89, 21.99, 20.32, 19.82, 18.92, 16.45, 16.16, 13.38. HRMS (*m*/*z*): calcd for C_33_H_48_ClNO_7_P^+^ [M + H]^+^: 636.2851, found: 636.2846.

*2-(((diethoxyphosphoryl)(4-methoxyphenyl)methyl)amino)-2-oxoethyl(4R,4aS,6aR,9S,11aR,11bS)-4,9,11b-trimethyl-8-oxotetradecahydro-6a,9-methanocyclohepta[a]naphthalene-4-carboxylate* (**6c**). White powder; yield, 81%; m.p. 134–135 °C; ^1^H-NMR (CDCl_3_, 300 MHz, ppm): δ 7.37–7.34 (m, 2H, Ar-H), 7.17–7.15 (m, 1H, -NH-CO-), 6.91–6.87 (m, 2H, Ar-H), 5.48 (dd, *J* = 21, 9 Hz, 1H, -P-CH-NH-), 4.72–4.62 (m, 1H, -O-CHe-CO-), 4.51–4.39 (m, 1H, -O-CHe-CO-), 4.18–4.07 (m, 2H, -O-CHe-), 4.00–3.90 (m, 1H, -O-CHa-), 3.80 (s, 3H, ph-OCH_3_), 3.76–3.66 (m, 1H, -O-CHa-), 2.60 (d, *J* = 18 Hz, 1H, 15-He), 2.25 (d, *J* = 12 Hz, 1H, 3-He), 2.05–1.98 (m, 1H, 6-He), 1.88–1.73 (m, 5H), 1.68–1.53 (m, 5H), 1.44–1.40 (m, 2H), 1.35–1.29 (m, 6H), 1.26–1.17 (m, 4H), 1.13–1.08 (m, 3H), 0.99–0.98 (m, 3H), 0.96–0.90 (m, 1H), 0.69 (s, 3H, 20-CH_3_). ^13^C-NMR (CDCl_3_, 75 MHz, ppm): δ 221.86, 175.71, 166.29, 159.63, 129.24(2C), 126.75, 114.19(2C), 63.57, 62.98, 62.75, 56.92, 55.31, 54.73, 54.21, 50.14, 48.67, 48.40, 43.96, 41.41, 39.64, 39.42, 38.02(2C), 37.21, 28.86, 21.95, 20.32, 19.82, 18.93, 16.39, 16.24, 13.31. HRMS (*m*/*z*): calcd for C_34_H_51_NO_8_P^+^ [M + H]^+^: 632.3347, found: 632.3344.

*2-(((diethoxyphosphoryl)(p-tolyl)methyl)amino)-2-oxoethyl(4R,4aS,6aR,9S,11aR,11bS)-4,9,11b-trimethyl-8-oxotetradecahydro-6a,9-methanocyclohepta[a]naphthalene-4-carboxylate* (**6d**). White powder; yield, 84%; m.p. 119–120 °C; ^1^H-NMR (CDCl_3_, 300 MHz, ppm): δ 7.32–7.29 (m, 2H, Ar-H), 7.18–7.11 (m, 3H, Ar-H, -NH-CO-), 5.44 (dd, *J* = 21, 9 Hz, 1H, -P-CH-NH-), 4.74–4.62 (m, 1H, -O-CHe-CO-), 4.51–4.38 (m, 1H, -O-CHe-CO-), 4.18–4.06 (m, 2H, -O-CHe-), 4.00–3.87 (m, 1H, -O-CHa-), 3.78–3.64 (m, 1H, -O-CHa-), 2.60 (dq, *J* = 18, 3 Hz, 1H, 15-He), 2.34 (s, 3H, ph-CH_3_), 2.26 (d, *J* = 15 Hz, 1H, 3-He), 2.07–1.99 (m, 1H, 6-He), 1.89–1.78 (m, 3H), 1.73–1.69 (m, 3H), 1.64–1.54 (m, 4H), 1.49–1.40 (m, 3H), 1.35–1.29 (m, 6H), 1.26–1.22 (m, 3H), 1.13–1.08 (m, 3H), 1.00–0.99 (m, 3H), 0.95–0.91 (m, 1H), 0.69 (s, 3H, 20-CH_3_). ^13^C-NMR (CDCl_3_, 75 MHz, ppm): δ 221.98, 175.68, 166.28, 138.26, 131.68, 129.48(2C), 127.86(2C), 63.48, 63.08, 62.79, 57.00, 54.74, 54.24, 50.53, 48.68, 48.41, 43.97, 41.36, 39.65, 39.42, 38.02(2C), 37.24, 28.96, 22.16, 21.14, 20.32, 19.82, 18.95, 16.42, 16.13, 13.30. HRMS (*m*/*z*): calcd for C_34_H_51_NO_7_P^+^ [M + H]^+^: 616.3398, found: 616.3393.

*2-(((3,4-dichlorophenyl)(diethoxyphosphoryl)methyl)amino)-2-oxoethyl(4R,4aS,6aR,9S,11aR,11bS)-4,9,11b-trimethyl-8-oxotetradecahydro-6a,9-methanocyclohepta[a]naphthalene-4-carboxylate* (**6e**). White powder; yield, 80%; m.p. 168–169 °C; ^1^H-NMR (CDCl_3_, 300 MHz, ppm): δ 7.50–7.44 (m, 2H, Ar-H), 7.26–7.24 (m, 1H, Ar-H), 7.08–7.03 (m, 1H, -NH-CO-), 5.40 (ddd, *J* = 21, 9, 3 Hz, 1H, -P-CH-NH-), 4.78–4.65 (m, 1H, -O-CHe-CO-), 4.54–4.41 (m, 1H, -O-CHa-CO-), 4.18–4.10 (m, 2H, -O-CHe-), 4.07–3.99 (m, 1H, -O-CHa-), 3.92–3.81 (m, 1H, -O-CHa-), 2.66–2.58 (m, 1H, 15-He), 2.27 (d, *J* = 15 Hz, 1H, 3-He), 2.06–1.97 (m, 1H, 6-He), 1.89–1.66 (m, 6H), 1.61 (s, 2H), 1.58–1.50 (m, 2H), 1.46–1.41 (m, 2H), 1.36–1.30 (m, 6H), 1.27–1.11 (m, 7H), 1.01–1.00 (m, 3H), 0.98–0.92 (m, 1H), 0.72–0.71 (m, 3H, 20-CH_3_). ^13^C-NMR (CDCl_3_, 75 MHz, ppm): δ 221.98, 175.83, 166.68, 135.15, 132.96, 132.62, 130.75, 129.67, 127.25, 63.81, 63.50, 62.81, 56.95, 54.78, 54.27, 50.01, 48.67, 48.39, 44.05, 41.40, 39.70, 39.48, 38.02, 37.91, 37.29, 28.92, 21.99, 20.41, 19.91, 18.90, 16.42, 16.24, 13.39. HRMS (*m*/*z*): calcd for C_33_H_47_Cl_2_NO_7_P^+^ [M + H]^+^: 670.2462, found: 670.2458.

### 3.8. General Procedure for the Synthesis of Intermediates (IIa-IIe, IVa-IVj, Va-Ve, VIa-VIe)

**Scheme 2:**
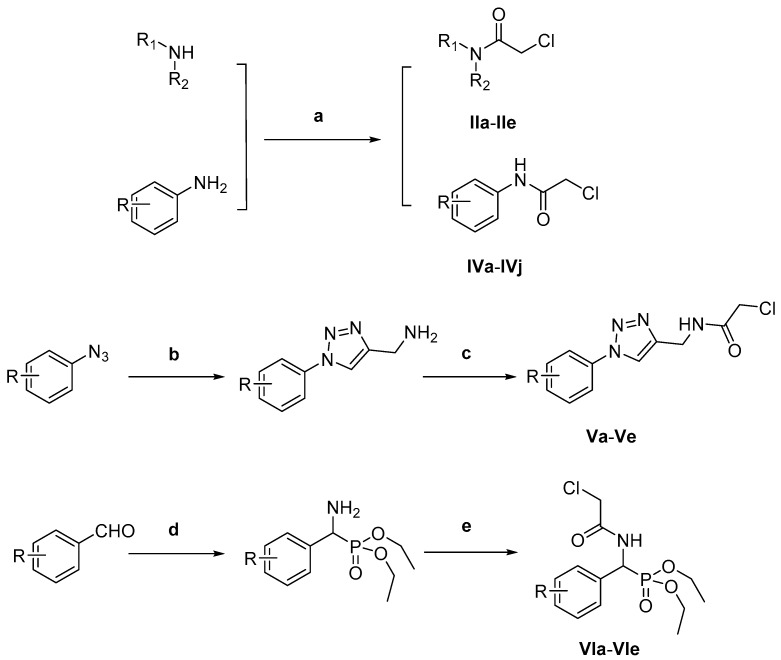
Reagents and conditions: (**a**) chloroacetyl chloride, Et_3_N, CH_2_Cl_2_, 30 °C; (**b**) (i) propynylamine, CuSO_4_^.^5H_2_O, sodium ascorbate, t-BuOH/H_2_O (1:1), 30 °C; (**c**) chloroacetyl chloride, Et_3_N, CH_2_Cl_2_, 30 °C; (**d**) diethyl phosphite, CH_3_COONH_4_, 90 °C; (**e**) chloroacetyl chloride, Et_3_N, CH_2_Cl_2_, 30 °C.

Chloroacetyl chloride (1.23 g, 11 mmol), Et_3_N (1.11 g, 11 mmol) and different amines (10 mmol) were added to CH_2_Cl_2_ (20 mL) and the resulting mixture was stirred at 30 °C for 2–8 h. Then, the solvent was evaporated in vacuo, water was added (15 mL), the mixture was filtered and the residue was washed with water to obtain the different intermediates (Scheme 2).

### 3.9. Biological Evaluation Materials

3-[4,5-Dimethylthiazol-2-yl]-2,5-diphenyl-tetrazolium bromide (MTT) was purchased from Sigma Chemical Co. (St. Louis, MO, USA). The propidium iodide (PI) and Annexin V-FITC apoptosis detection kit was purchased from BD Pharmingen (San Diego, CA, USA).

### 3.10. In Vitro Antiproliferative Activity

The antiproliferative activity of the target compounds against a normal L02 cell line and the three different human cancer cell lines viz. colorectal (HCT-116), liver (BEL-7402) and liver (HepG2) were evaluated using a standard MTT-based colorimetric assay. All cell lines were obtained from the Key Laboratory of Natural Resources and Functional Molecules of the Changbai Mountain (Yanbian University, Jilin, China) and maintained in Dulbecco’s modified Eagle’s medium (DMEM) and RPMI Media 1640 (RPMI1640), supplemented with 10% fetal bovine serum (FBS) at 37 °C in a humidified atmosphere containing 5% CO_2_.

Cells were plated in 96-well plates at appropriate densities to ensure exponential growth throughout the experimental period (9 × 10^3^ cells per well) and then allowed to adhere for 24 h. Cells were then treated for 48 h with four serial concentrations (1, 10, 50 and 100 µM) of each compound. 5-fluorouracil (5-FU) was used as a positive control. After 48 h of incubation, 10 µL of MTT solution was added to each well to a final concentration of 2 mg/mL. Plates were then incubated for a further 4 h. After incubation, the MTT solution was removed and 150 µL of DMSO was added to each well for coloration. The plates were shaken vigorously for 10 min at room temperature to ensure complete solubilization. The optical density (OD) was read on a microplate reader (ELx800, BioTek, Highland Park, Winooski, VT, USA) at a wavelength of 492 nm and the data were subsequently analyzed. The percentage of cell growth inhibition was calculated from the following equation:

Inhibitory rate (%) = [(1 − (OD_treated_-OD_blank_)/(OD_control_-OD_blank_)] × 100. (1)

### 3.11. Colony Formation Assay

Exponentially growing determined HCT-116 cells (6 × 10^2^ per well) were plated in 6-well plates with the Roswell Park Memorial Institute (RPMI) 1640 culture medium (Gibco, USA) containing with 10% fetal bovine serum (FBS) (Gibco, Waltham, MA, USA). After an overnight incubation, the culture medium was removed and replaced fresh medium. Then, the cells were treated with various concentrations of compound **5d** (5, 10 and 15 μM) dissolved in DMSO. Some cells were treated with DMSO only as a negative control. The cells were then incubated for another 7 days. Finally, the cells were fixed with 4% paraformaldehyde for 30 min and stained with 0.1% crystal violet for 15 min at room temperature, after which, the staining was washed with PBS until the colonies were totally cleared.

### 3.12. Analysis for Cell Cycle and Apoptosis by Flow Cytometry

HCT-116 cells were plated in 6-well plates (5.0 × 10^5^ cells per well) and incubated at 37 °C for 24 h. Exponentially growing cells were then incubated with compound **5d** at 5 and 15 µM. After 24 h, untreated cells (control) or cells treated with compound **5d** were centrifuged at 1000 rpm for 10 min and then fixed in 70% ethanol at −20 °C for at least 24 h. The cells were subsequently resuspended in phosphate-buffered saline (PBS) containing 0.1 mg/mL of RNase A and 5 µg/mL propidium iodide (PI). The cellular DNA content for the cell cycle distribution analysis was measured by flow cytometry using a FACSCalibur flow cytometer with Cell Quest software (Becton-Dickinson, Franklin Lakes, NJ, USA), plotting at least 30,000 events per sample. The percentage of cells in the G1, S and G2 phases of the cell cycle were determined using the ModFit LT version 4.0 software package (Verity Software, Topsham, ME, USA).

Apoptosis was detected using an Apoptosis Detection Kit (Invitrogen, Eugene, OR, USA). In brief, cells were cultured in 6-well plates (5.0 × 10^5^ cells per well) and incubated at 37 °C for 24 h. Cells with exponential growth were then incubated with compound **5d** at 5 and 15 µM. Following 24 h of incubation, the cells were collected, washed twice with PBS and once with 1 × binding buffer and then stained with 5 µM of annexin V-FITC and 2.5 µM of PI 5mg/mL in 1 × binding buffer for 30 min at room temperature in the dark. Apoptotic cells were enumerated using a FACSCalibur flow cytometer with Cell Quest software (Becton–Dickinson, Franklin Lakes, NJ, USA).

### 3.13. Western Blotting

HCT-116 cells were cultured with different concentrations of compound **5d** for 24 h. Then, the floating cells was collected and washed two times with ice cold PBS. The pellet was resuspended in lysis buffer. After the cells were lysed on ice for 20 min, lysates were centrifuged at 12,000 rpm at 4 °C for 15 min. The protein concentration in the supernatant was determined using BCA protein assay reagents. Equal amounts of protein (50 μg) were resolved using sodium dodecyl sulphate-polyacrylamide gel electrophoresis (SDS-PAGE) (8−12% acrylamide gels) and transferred to a PVDF Hybond-P membrane. Membranes were blocked with PBS containing 5% non-fat milk for 1 h at room temperature. Membranes were then incubated with primary antibodies against Cyclin A (ENT1167, Elabscience, Wuhan, China), Cyclin E1 (ENT1176, Elabscience, Wuhan, China), Cyclin B1 (ENT1169, Elabscience, Wuhan, China) and β-actin (ENM0028, Elabscience, Wuhan, China), with gentle rotation overnight at 4 °C. Membranes were next incubated with fluorescent secondary antibodies for 2 h. Proteins were detected by electrochemiluminescence (Bio-Rad, Hercules, CA, USA).

### 3.14. Molecular Docking Study

The molecular docking study was performed using Discovery Studio (DS) 2017. The protein and ligand were prepared, water molecules were deleted and a DS Server added hydrogen. The docking result was treated with DS Client. In this study, the crystal structure of the CDK2/cyclin A complex (3my5) was chosen for docking. The xyz coordinates (−25.8518, 4.7751, −23.0309, radius 34.6 Å) of CDK2/cyclin A complex residues were defined as the binding site sphere. The protocol, CDOCKER was used to perform the docking. The output poses of the ligands generated were analyzed based on the LibDockScore function.

## 4. Conclusions

In the present study, thirty-two novel isosteviol derivatives were designed, synthesized and evaluated for their antiproliferative effects against three cancer cell lines and the normal human L02 cell line. Half of these prepared compounds displayed some cytotoxic activity against the three cancer cell lines, with low cytotoxicity against the normal L02 line. In particular, compound **5d** exhibited the most potent inhibitory activity against the three cancer cell lines. In addition, compound **5d** can inhibit the colony formation of HCT-116 cells in a concentration-dependent manner. Cell cycle analysis revealed that compound **5d** inhibited cell growth via the induction of S phase arrest in HCT-116 cells. The possible mechanism of action may be correlated with down-regulation of cyclin A and cyclin E1 expression with the up-regulation of cyclin B1 expression. Our docking study also revealed the amino acids His-C71 and Lys-A56 are playing a crucial role in the binding of compound **5d** within the active site of the CDK2/cyclin A complex. Hence, compound **5d** may be a valuable candidate for further studies aimed at the development of effective and harmless anticancer drugs.

## Figures and Tables

**Table 1:** The antiproliferative activity of compounds (% Growth Inhibition at 100 μM).

Compound	% Growth Inhibition at 100 μM		
	HCT-116	BEL-7402	HepG2
**1a**	65.22 ± 2.52	87.10 ± 2.37	66.02 ± 1.33
**1b**	76.61 ± 1.78	88.51 ± 2.02	56.96 ± 2.35
**1c**	NA	31.34 ± 1.22	NA
**2a**	62.06 ± 1.02	65.04 ± 2.62	40.61 ± 0.70
**2b**	60.37 ± 1.69	64.36 ± 1.66	NA
**2c**	15.71 ± 0.61	40.46 ± 0.87	NA
**2d**	75.37 ± 1.06	70.86 ± 1.05	70.63 ± 0.89
**2e**	85.61 ± 0.96	64.87 ± 1.01	50.36 ± 0.57
**3a**	86.26 ± 0.59	80.01 ± 0.95	75.25 ± 0.56
**3b**	70.75 ±0.98	61.76 ± 0.96	81.15 ± 2.66
**3c**	54.71 ± 0.59	66.62 ± 1.90	23.87 ± 0.85
**3d**	19.66 ± 1.11	35.78 ± 2.37	NA
**4a**	60.44 ± 3.11	50.78 ± 2.31	31.18 ± 1.19
**4b**	NA	NA	NA
**4c**	62.34 ± 3.55	35.68 ± 2.56	NA
**4d**	56.85 ± 2.35	52.92 ± 2.11	31.91 ± 1.31
**4e**	27.87 ± 0.44	38.66 ± 1.52	NA
**4f**	68.74 ± 2.67	54.58 ± 3.11	49.12 ± 0.44
**4g**	65.64 ± 3.01	62.96 ± 2.13	39.11 ± 1.13
**4h**	55.35 ± 2.55	71.38 ± 2.36	76.46 ± 1.00
**4i**	53.14 ± 2.56	45.62 ± 1.47	NA
**4j**	NA	31.30 ± 1.76	NA
**5a**	81.87 ± 2.55	90.02 ± 1.92	87.03 ± 1.06
**5b**	85.60 ± 1.34	90.49 ± 1.04	82.10 ± 1.82
**5c**	84.64 ± 1.55	75.56 ± 1.76	82.85 ± 2.02
**5d**	84.61 ± 0.96	86.46 ± 0.87	82.77 ± 0.86
**5e**	71.14 ± 0.65	67.96 ± 1.17	66.10 ± 0.97
**6a**	50.05 ± 2.27	33.66 ± 0.91	28.02 ± 0.99
**6b**	50.34 ± 2.31	50.36 ± 1.63	40.22 ± 2.56
**6c**	74.07 ± 2.45	50.87 ± 2.68	41.40 ± 1.86
**6d**	76.63 ± 2.33	51.46 ± 0.68	80.64 ± 0.76
**6e**	43.45 ± 0.56	49.88 ± 1.26	39.47 ± 1.37
**Isosteviol**	34.09 ± 1.89	28.04 ± 1.79	11.79 ± 0.88

NA: antiproliferative activity < 10%. % Growth Inhibition are presented as the mean ± SD (standard error of the mean) from three separated experiment.

**Table 2:** IC_50_ values (μM) of several active compounds.

Compound	IC_50_ Values (μM) ^a^			
	HCT-116	BEL-7402	HepG2	L02
**1b**	24.04 ± 1.20	>100	>100	>100
**2b**	30.71 ± 1.98	>100	>100	>100
**2d**	18.18 ± 1.26	52.98 ± 1.97	11.05 ± 0.55	>100
**2e**	>100	13.22 ± 0.32	20.51 ± 0.46	>100
**3a**	40.41 ± 0.97	>100	>100	>100
**3b**	22.78 ± 0.81	36.49 ± 0.67	24.40 ±1.75	>100
**4a**	56.56 ± 1.56	>100	>100	>100
**4f**	17.22 ± 0.43	>100	>100	>100
**4h**	>100	12.14 ± 0.12	14.11 ± 0.27	>100
**5a**	14.63 ± 0.51	27.10 ± 0.87	9.87 ± 0.13	>100
**5b**	7.44 ± 0.37	22.50 ± 0.58	11.67 ± 0.23	>100
**5c**	14.40 ± 1.31	>100	12.27 ± 0.77	>100
**5d**	5.38 ± 0.26	15.91 ± 0.41	8.92 ± 0.44	>100
**5e**	14.32 ± 0.15	>100	12.82 ± 0.09	>100
**6c**	15.15 ± 0.11	92.61 ± 1.96	>100	>100
**6d**	18.70 ± 0.17	60.93 ± 1.37	13.19 ± 0.86	>100
**Isosteviol**	>100	>100	>100	>100
**5-FU**	24.80 ± 2.08	21.30 ± 2.43	23.45 ± 0.37	19.12 ± 1.01

^a^ IC_50_: concentration that inhibits 50% of cell growth. The values are presented as the mean ± SD (standard error of the mean) from three separated experiments.

**Table 3:** In vitro antiproliferative activities of compound **5d** against normal cell line (L02).

Compound	(IC_50_, μM)	Selectivity Index ^a^		
	L02	HCT-116	BEL-7402	HepG2
**5d**	161.04 ± 1.96	29.93	10.12	18.05
**5-FU**	19.12 ± 1.01	0.77	0.90	0.82

^a^ SI: selective index (IC_50_ on normal cells/IC_50_ on cancer cells).

## Supplementary Materials

The supplementary materials are available online, 1H and 13C-NMR spectra of these compounds are available in the supplementary materials.
