# Supplementary material for: Design and Synthesis of C-19 Isosteviol Derivatives as Potent and Highly Selective Antiproliferative Agents
Source: Molecules. 2018 Dec 30;24(1):121. doi: 10.3390/molecules24010121 (PMC6337650; doi:10.3390/molecules24010121)
Supplement: Supplementary file 1 [file molecules-24-00121-s001.pdf]

Supplementary File

Design and Synthesis of C-19 Isosteviol Derivatives as Potent and Highly Selective Antiproliferative Agents

Tian Luan[a#], Li-Hua Cao[b#], Hao Deng[a], Qing-Kun Shen[a], Yu-Shun Tian[a]* & Zhe-Shan Quan[a]*

[a]*Key Laboratory of Natural Resources and Functional Molecules of the Changbai Mountain, Affiliated Ministry of Education, College of Pharmacy, Yanbian University, Yanji, Jilin, 133002, China*

[b]*Department of Pharmacology, College of Medicine, Yanbian University, Yanji, Jilin, 133002, China*

***Corresponding author: Tel: + 86 433 243-6020; Fax: + 86 433 243-6020.**

E-mail: zsquan@ybu.edu.cn (Z. S. Quan).

***Corresponding author: E-mail: ystian@ybu.edu.cn (Y. S. Tian).**

[#]These authors contributed equally to this work.

Table of contents:

^{1}H NMR and ^{13}C NMR spectra of compound **1a** page 3
^{1}H NMR and ^{13}C NMR spectra of compound **1b** page 4
^{1}H NMR and ^{13}C NMR spectra of compound **1c** page 5
^{1}H NMR and ^{13}C NMR spectra of compound **2a** page 6
^{1}H NMR and ^{13}C NMR spectra of compound **2b** page 7
^{1}H NMR and ^{13}C NMR spectra of compound **2c** page 8
^{1}H NMR and ^{13}C NMR spectra of compound **2d** page 9
^{1}H NMR and ^{13}C NMR spectra of compound **2e** page 10
^{1}H NMR and ^{13}C NMR spectra of compound **3a** page 11
^{1}H NMR and ^{13}C NMR spectra of compound **3b** page 12
^{1}H NMR and ^{13}C NMR spectra of compound **3c** page 13
^{1}H NMR and ^{13}C NMR spectra of compound **3d** page 14

^{1}H NMR and ^{13}C NMR spectra of compound **4a** page 15
^{1}H NMR and ^{13}C NMR spectra of compound **4b** page 16
^{1}H NMR and ^{13}C NMR spectra of compound **4c** page 17
^{1}H NMR and ^{13}C NMR spectra of compound **4d** page 18
^{1}H NMR and ^{13}C NMR spectra of compound **4e** page 19
^{1}H NMR and ^{13}C NMR spectra of compound **4f** page 20
^{1}H NMR and ^{13}C NMR spectra of compound **4g** page 21
^{1}H NMR and ^{13}C NMR spectra of compound **4h** page 22
^{1}H NMR and ^{13}C NMR spectra of compound **4i** page 23
^{1}H NMR and ^{13}C NMR spectra of compound **4j** page 24
^{1}H NMR and ^{13}C NMR spectra of compound **5a** page 25
^{1}H NMR and ^{13}C NMR spectra of compound **5b** page 26
^{1}H NMR and ^{13}C NMR spectra of compound **5c** page 27
^{1}H NMR and ^{13}C NMR spectra of compound **5d** page 28
^{1}H NMR and ^{13}C NMR spectra of compound **5e** page 29
^{1}H NMR and ^{13}C NMR spectra of compound **6a** page 30
^{1}H NMR and ^{13}C NMR spectra of compound **6b** page 31
^{1}H NMR and ^{13}C NMR spectra of compound **6c** page 32
^{1}H NMR and ^{13}C NMR spectra of compound **6d** page 33
^{1}H NMR and ^{13}C NMR spectra of compound **6e** page 34
HRMS spectra of compound **2d** page 35
HRMS spectra of compound **2e** page 36
HRMS spectra of compound **3b** page 37
HRMS spectra of compound **5b** page 38
HRMS spectra of compound **5c** page 39
HRMS spectra of compound **5d** page 40
HRMS spectra of compound **6c** page 41
HRMS spectra of compound **6d** page 42
^{1}H-NMR spectra of intermediate **Va** page 43

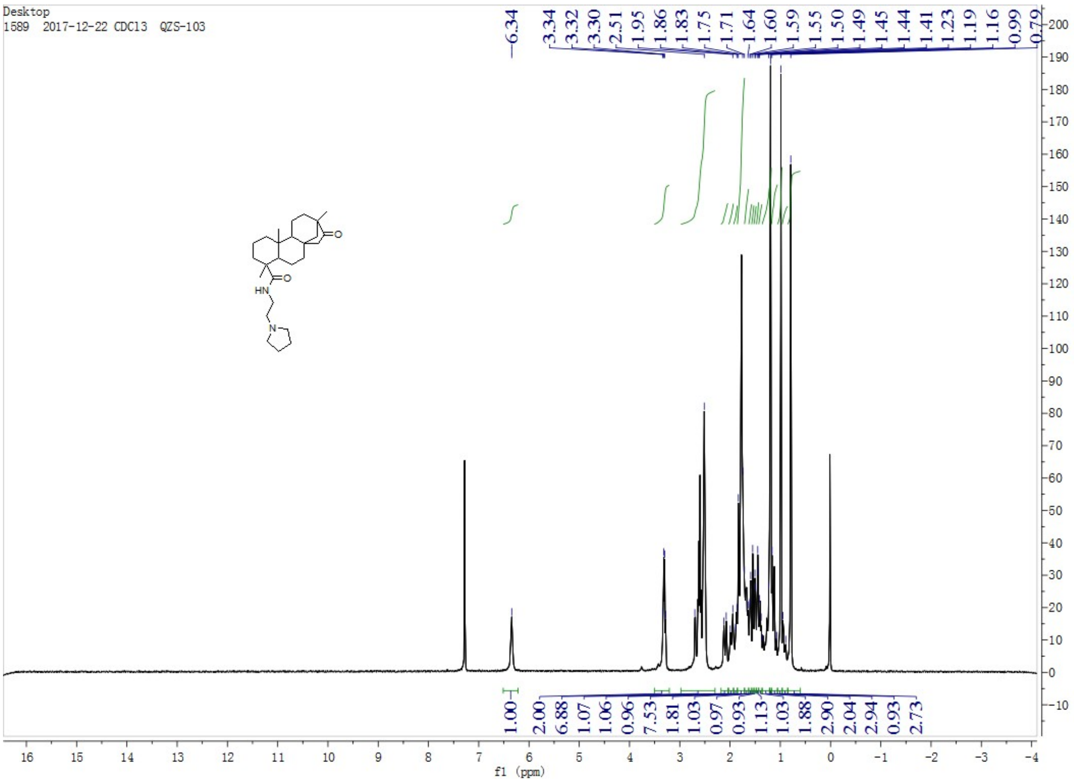

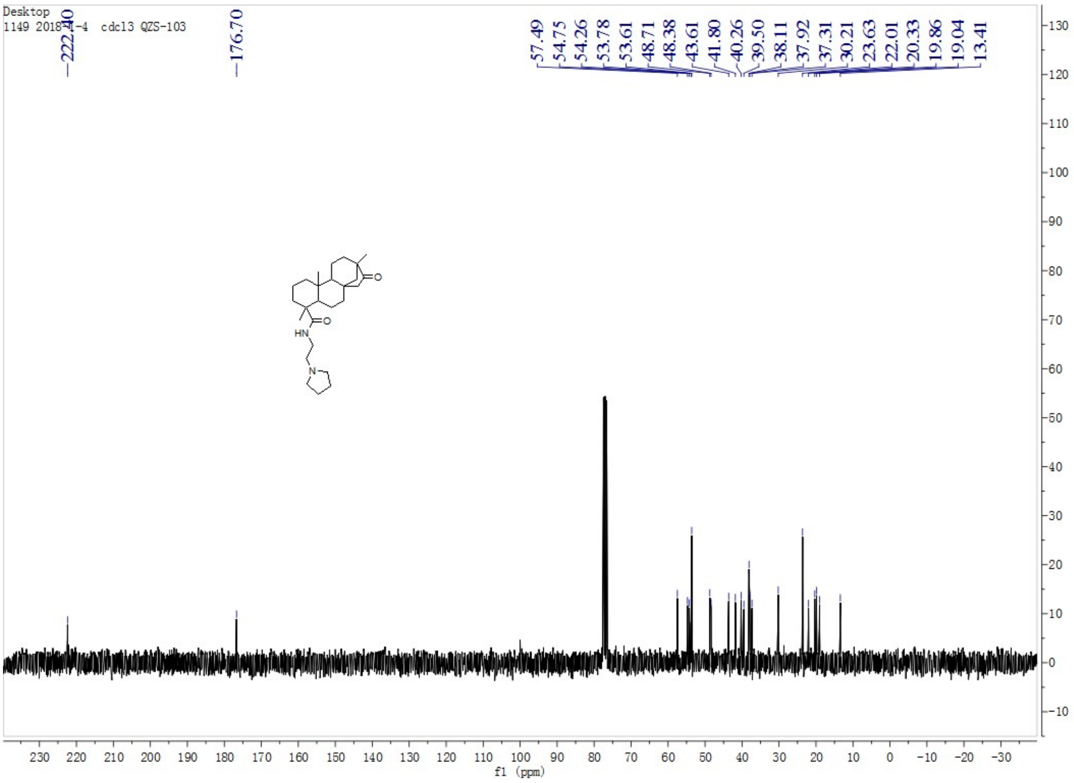

^{1}H NMR and ^{13}C NMR spectra of compound **1a**

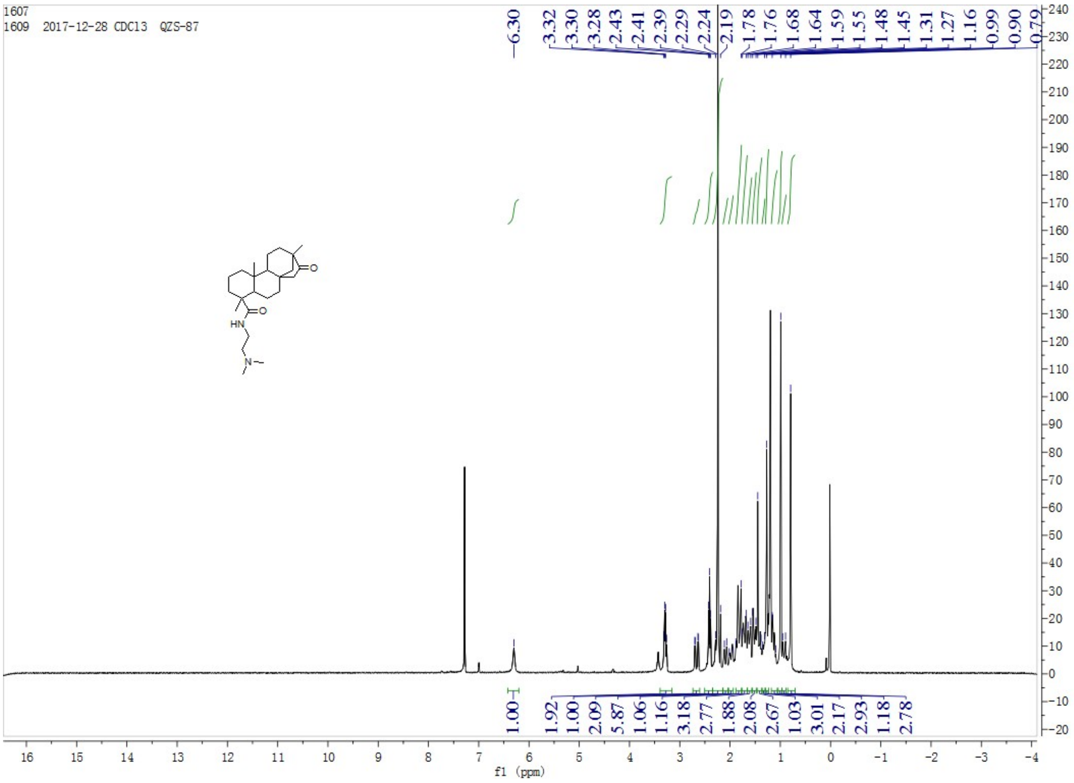

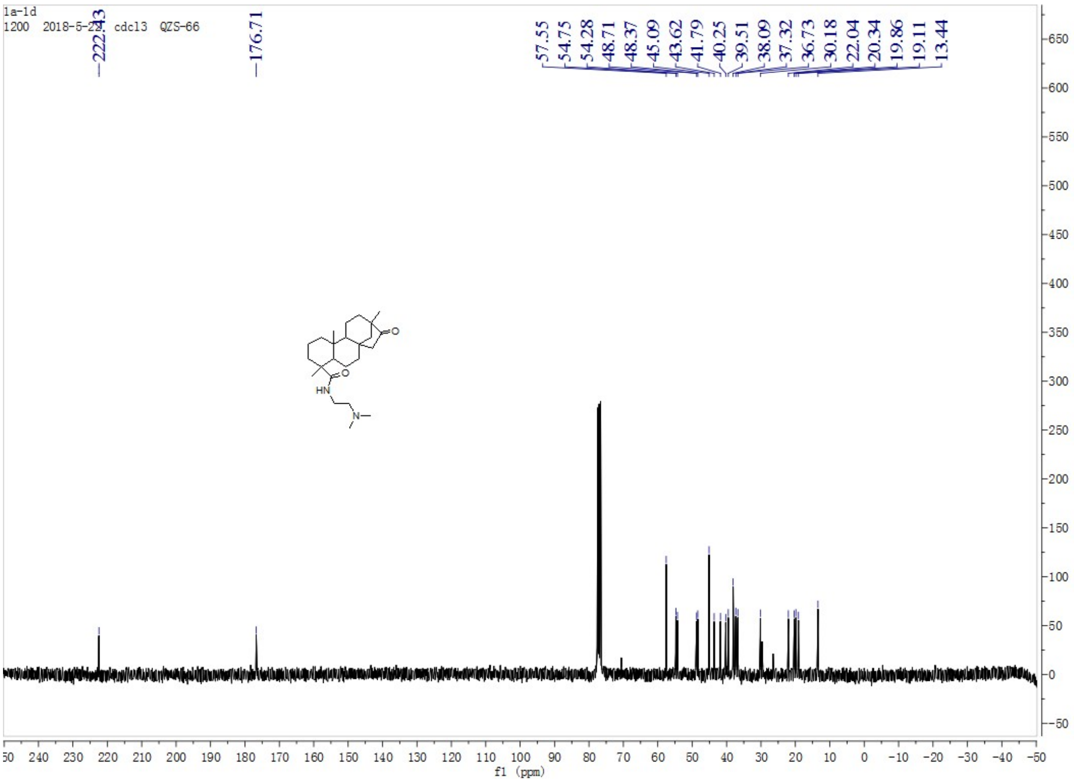

^{1}H NMR and ^{13}C NMR spectra of compound **2a**

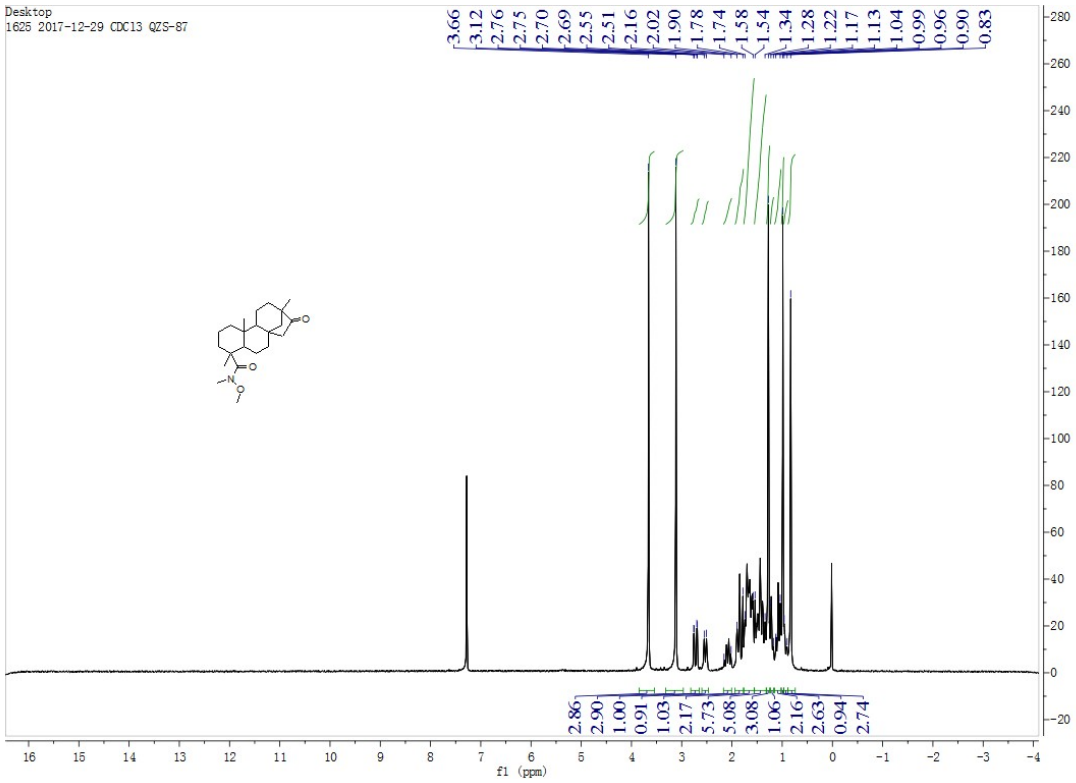

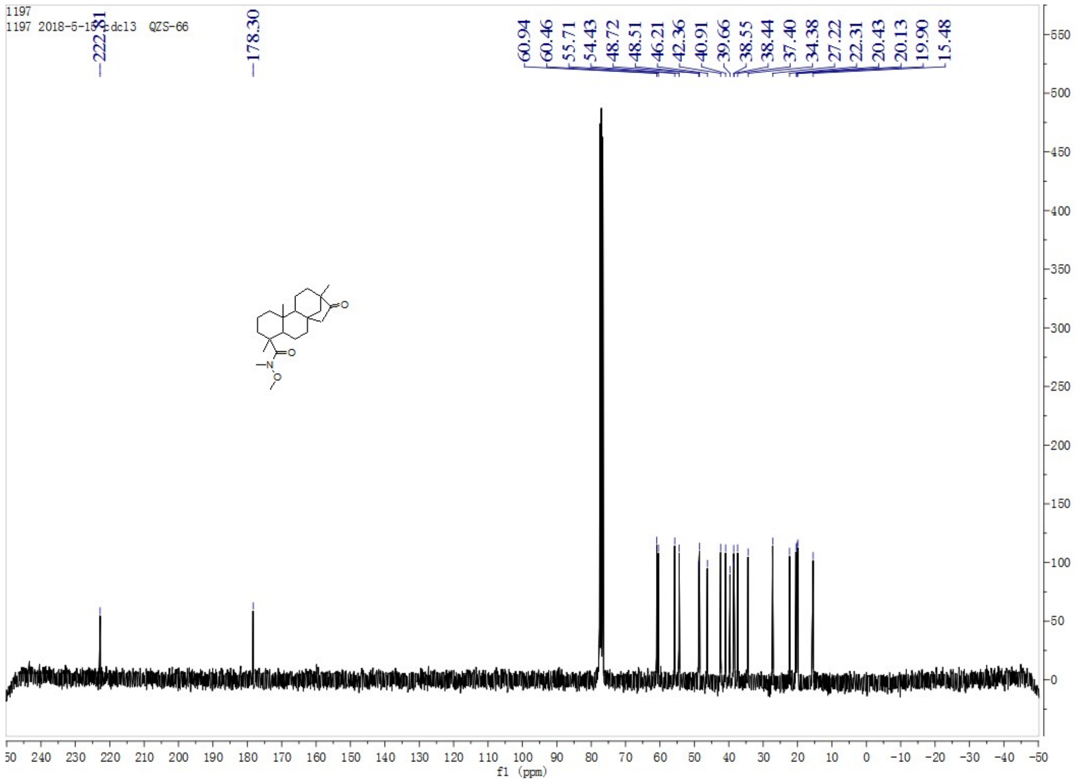

^{1}H NMR and ^{13}C NMR spectra of compound **1c**

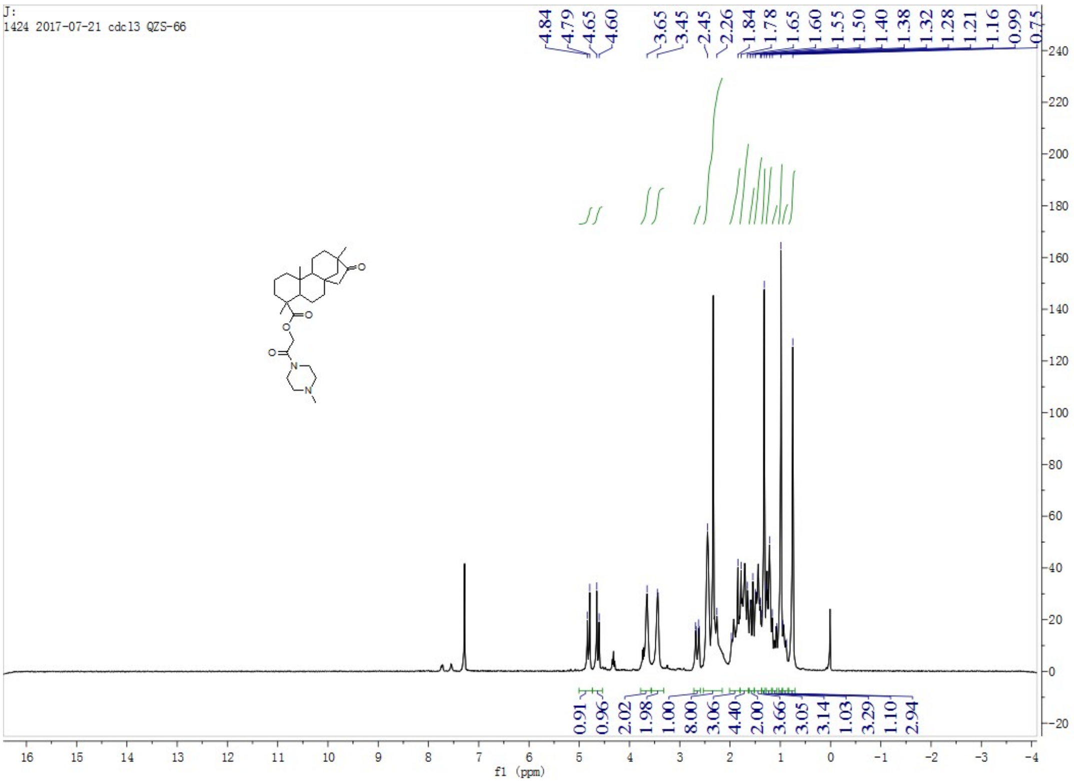

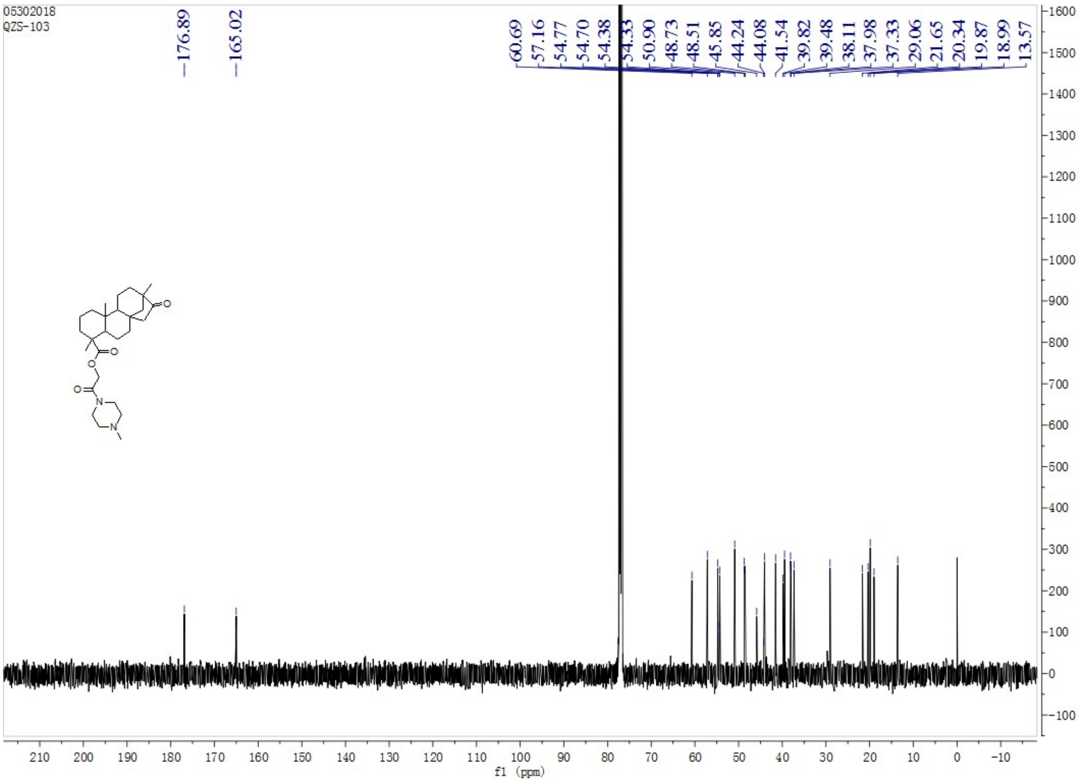

^{1}H NMR and ^{13}C NMR spectra of compound **2a**

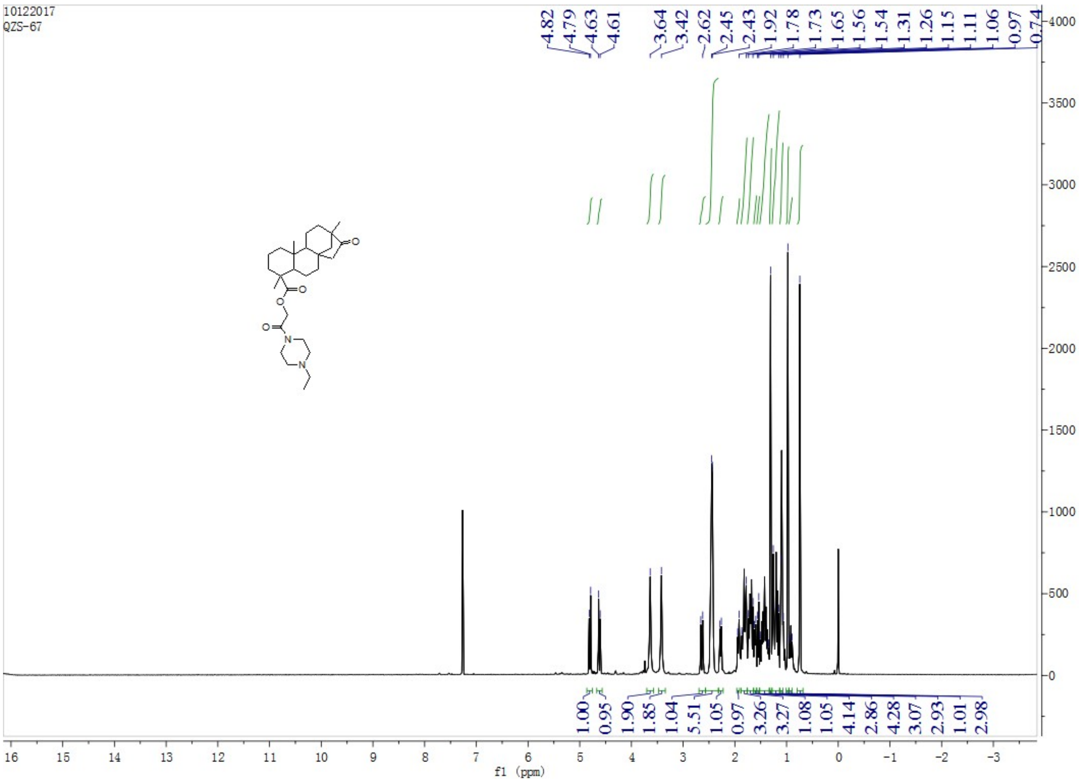

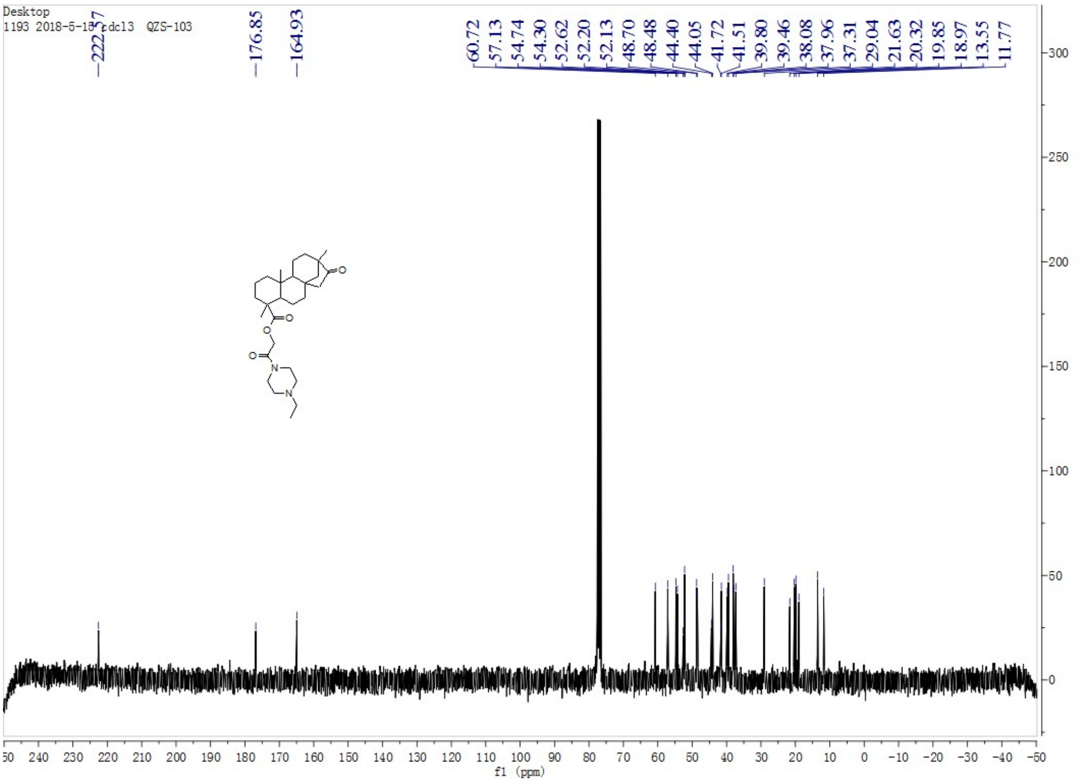

^{1}H NMR and ^{13}C NMR spectra of compound **2b**

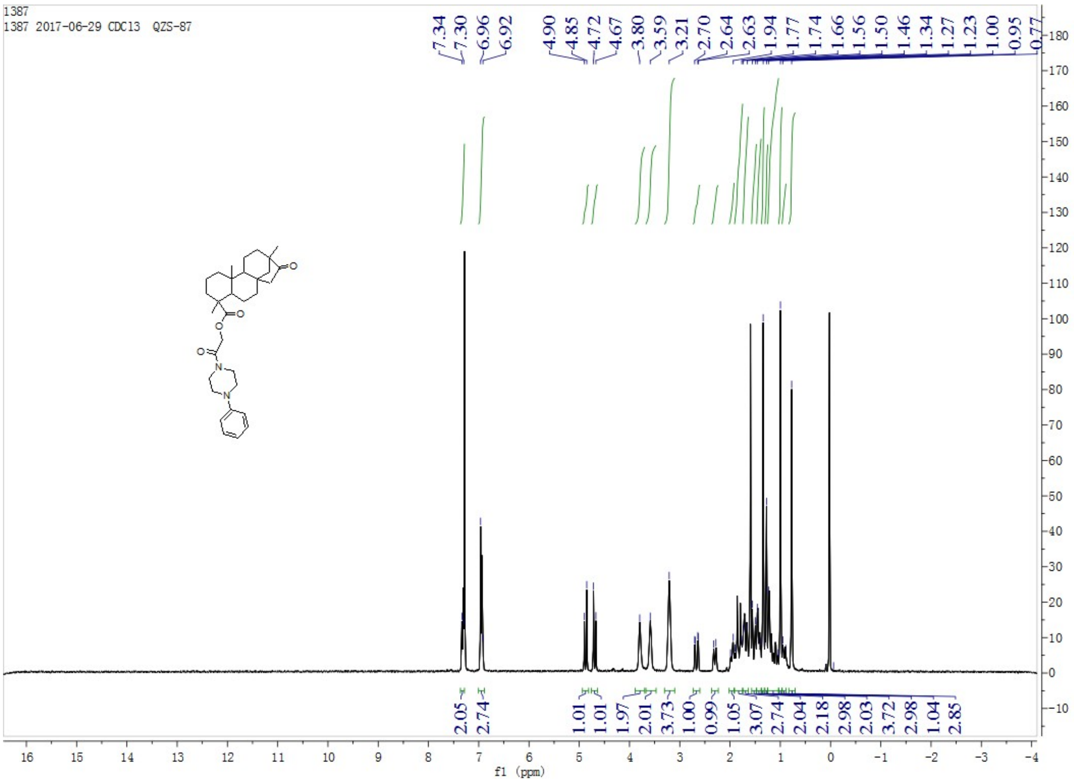

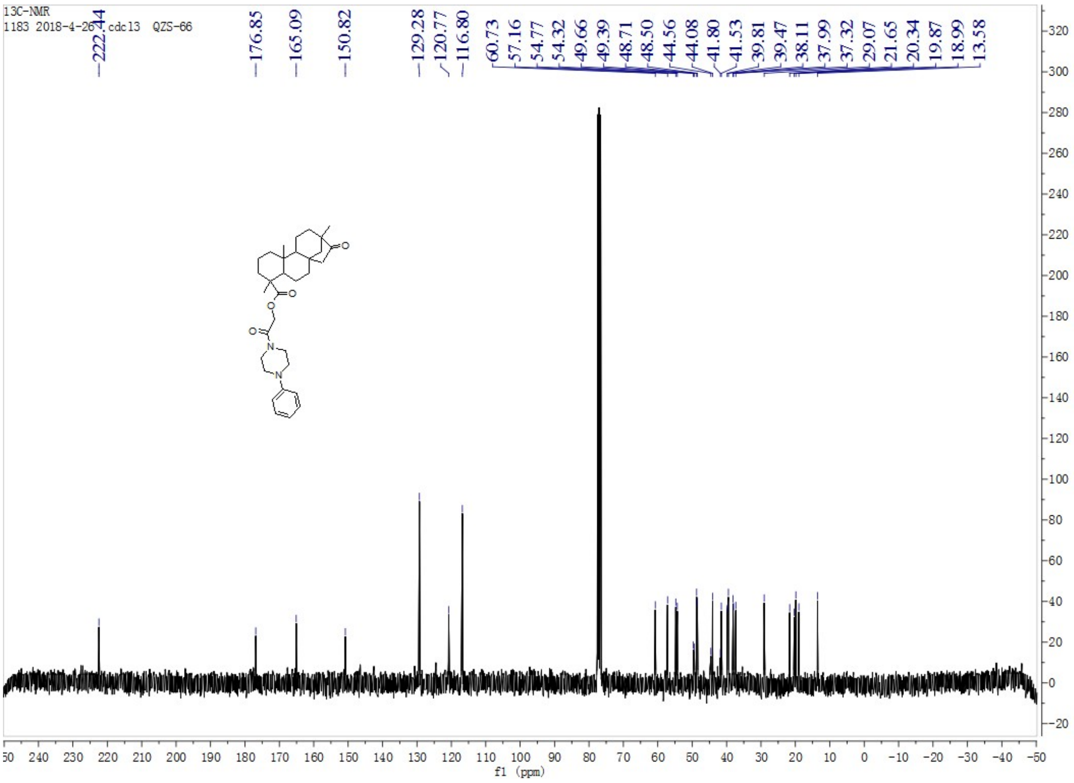

^{1}H NMR and ^{13}C NMR spectra of compound **2c**

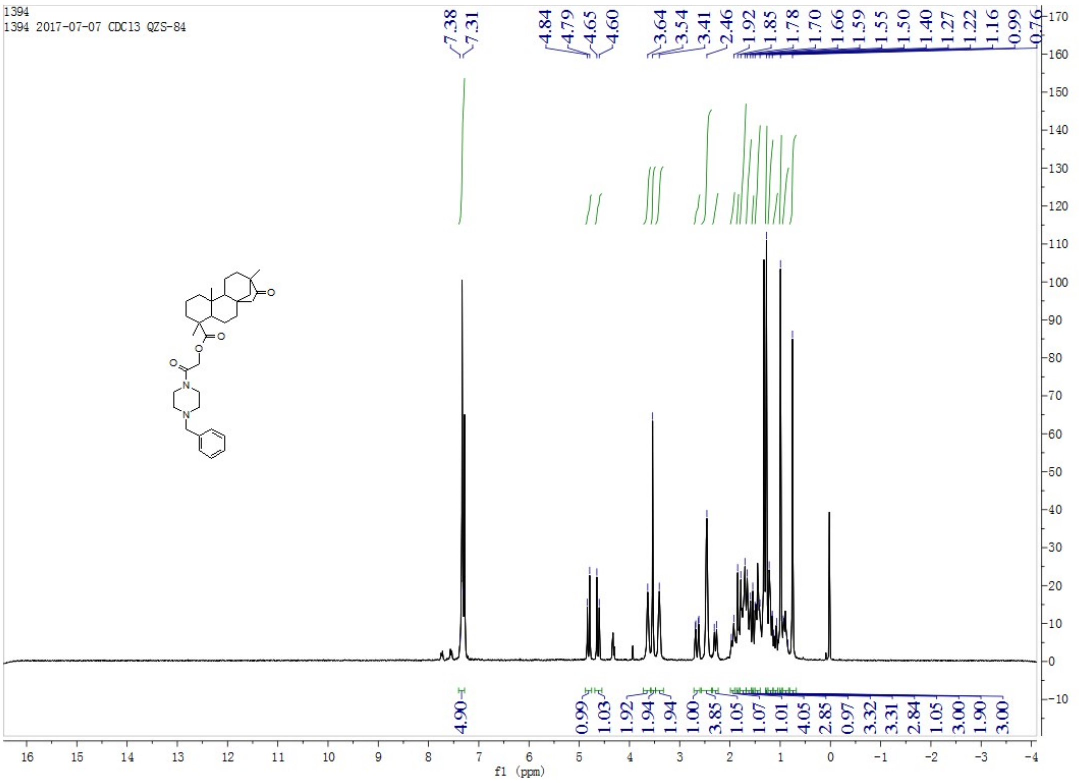

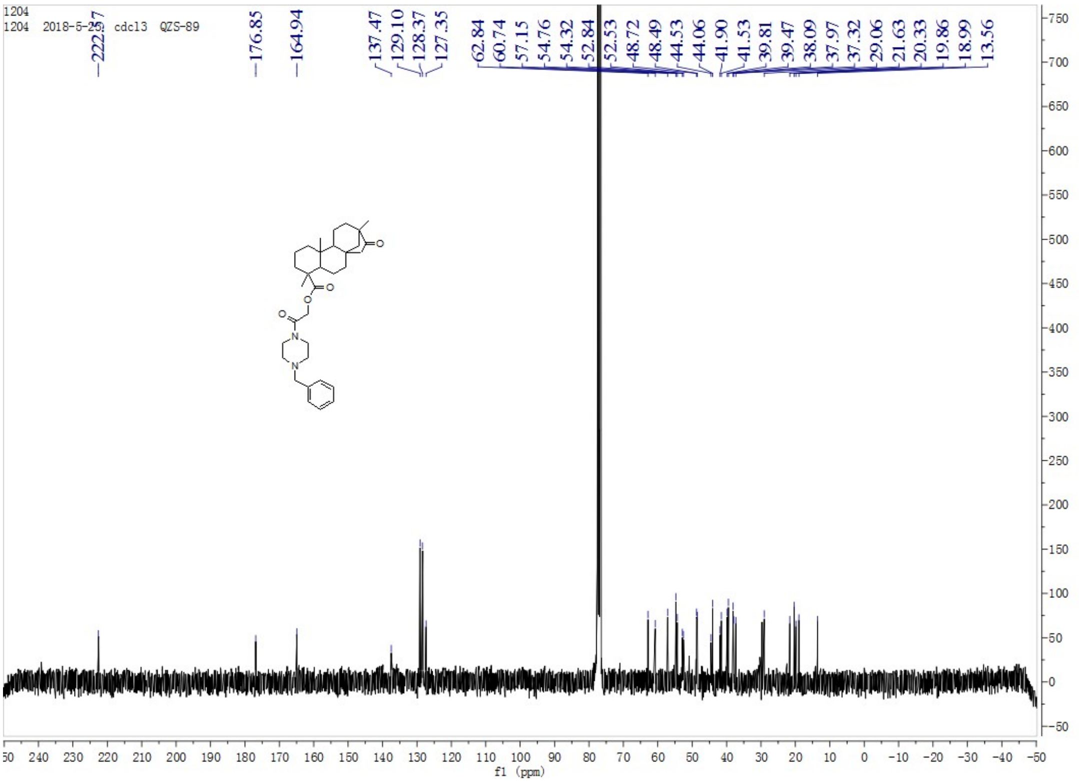

^{1}H NMR and ^{13}C NMR spectra of compound **2d**

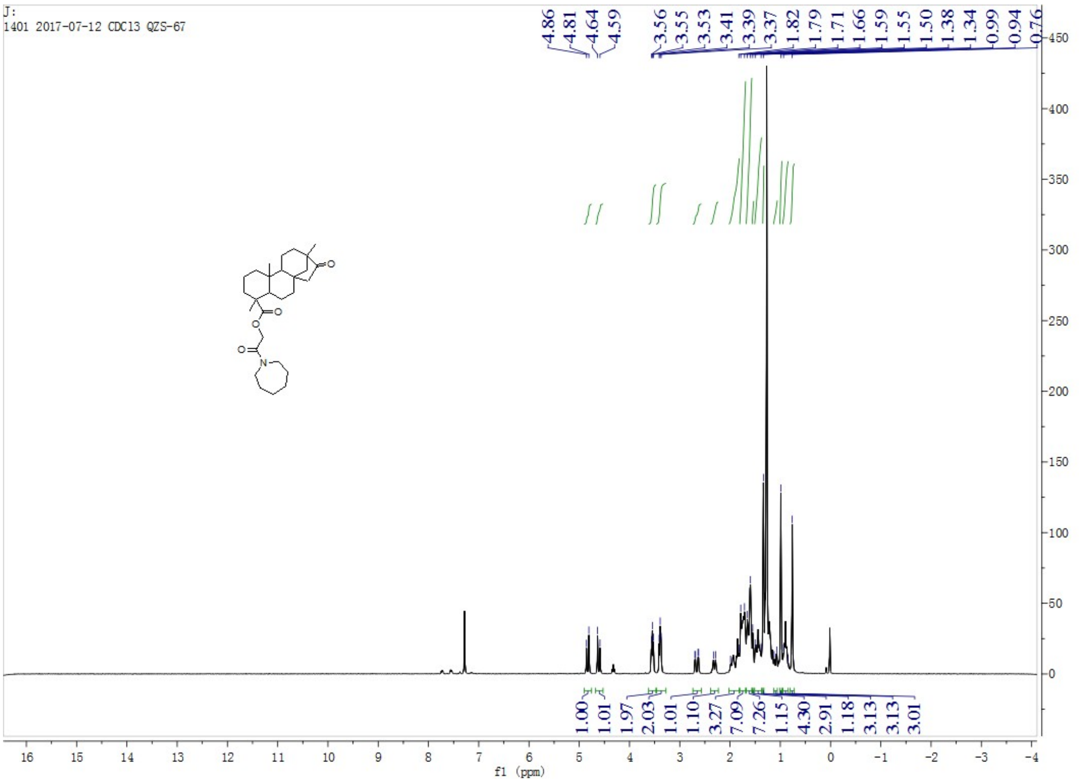

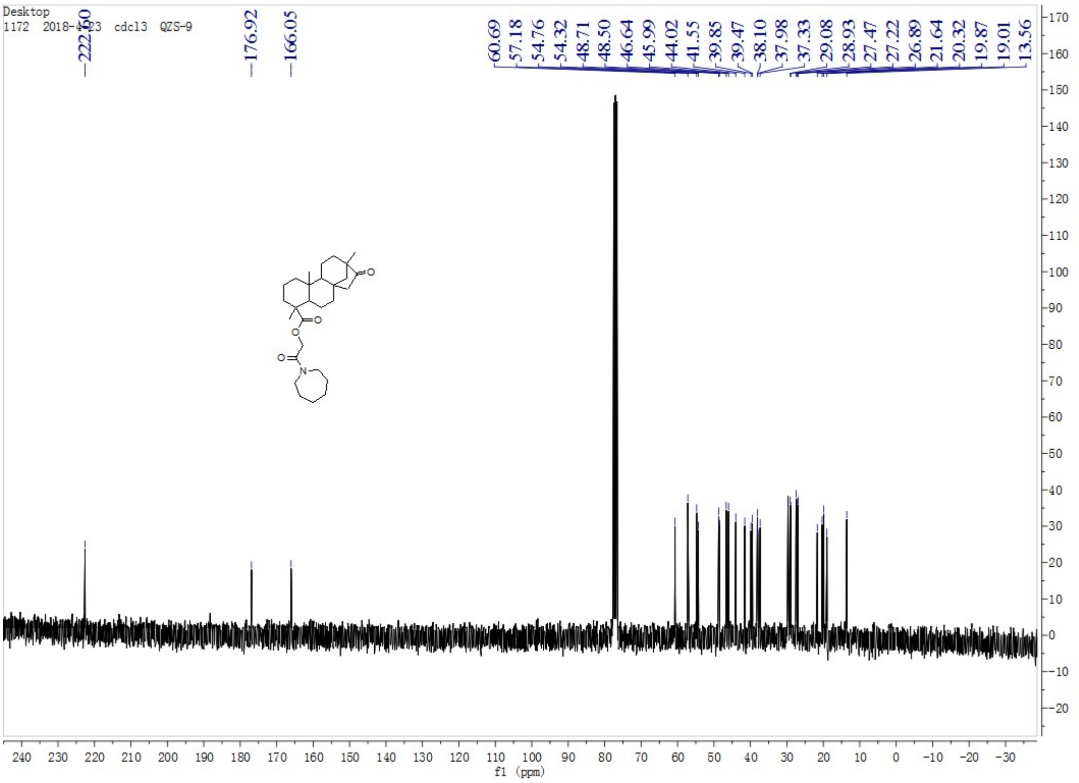

^{1}H NMR and ^{13}C NMR spectra of compound **2e**

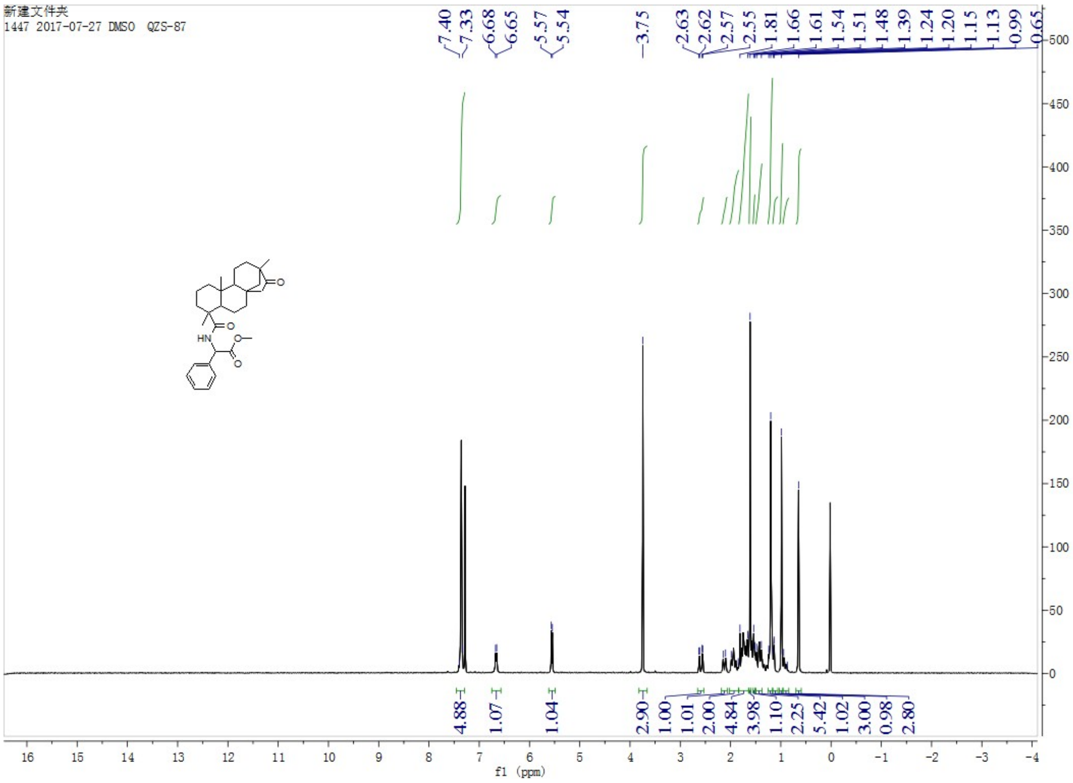

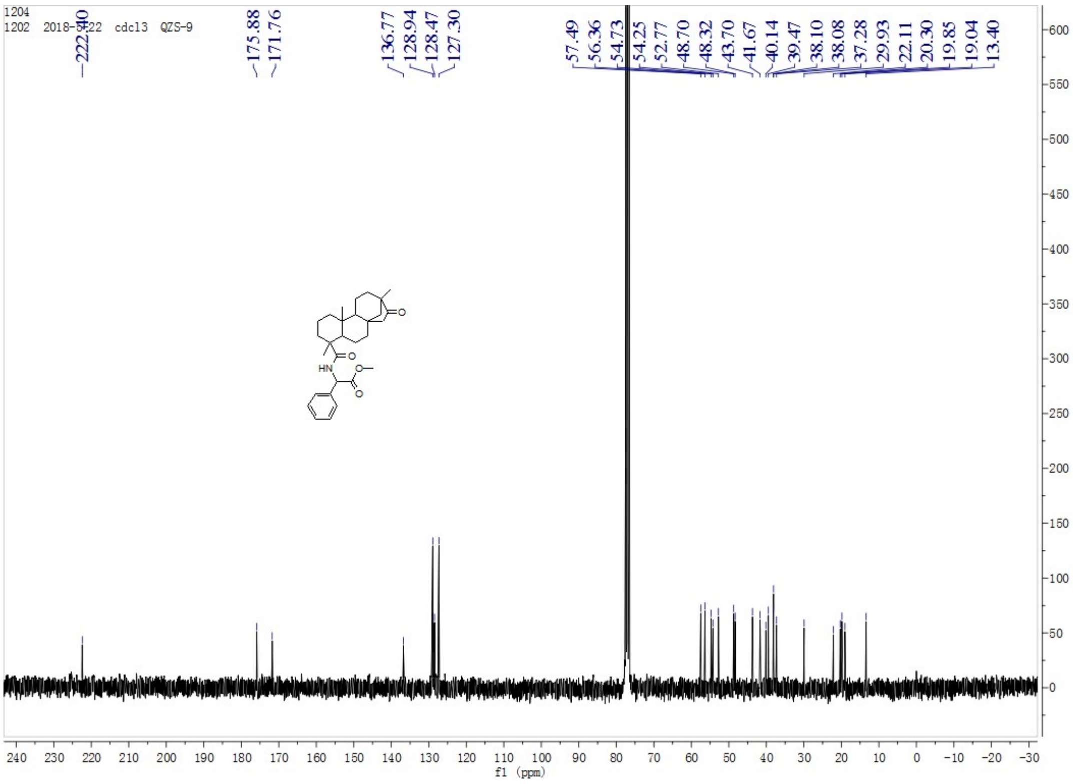

^{1}H NMR and ^{13}C NMR spectra of compound **3a**

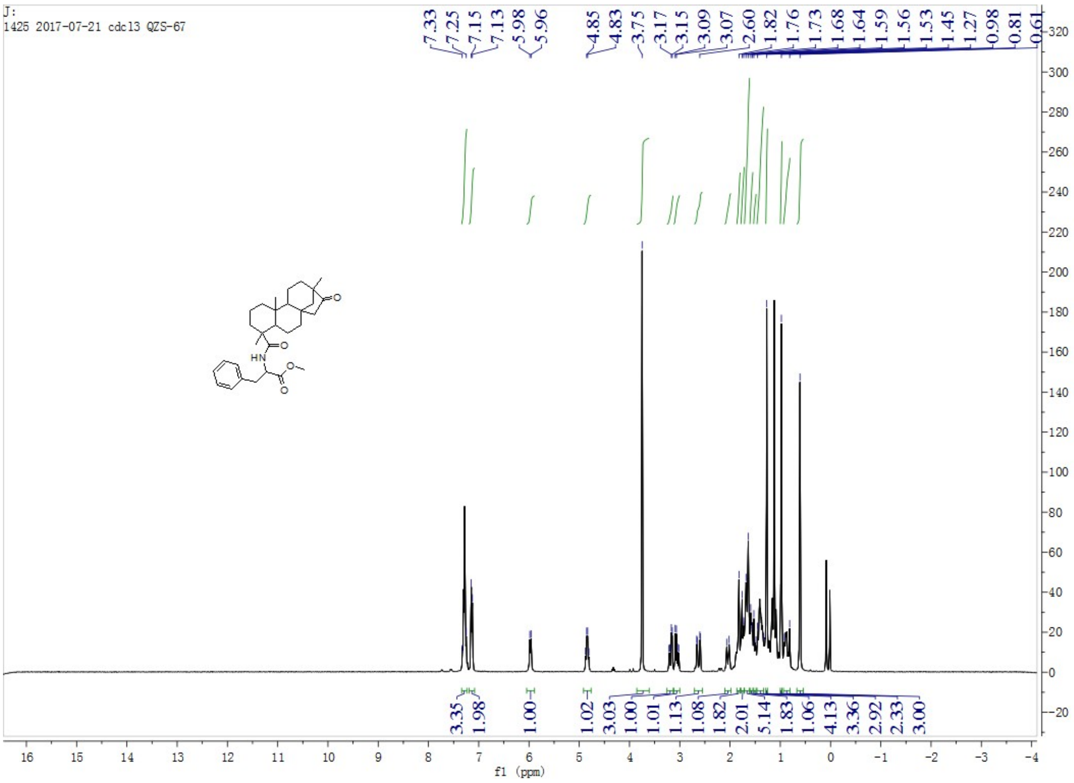

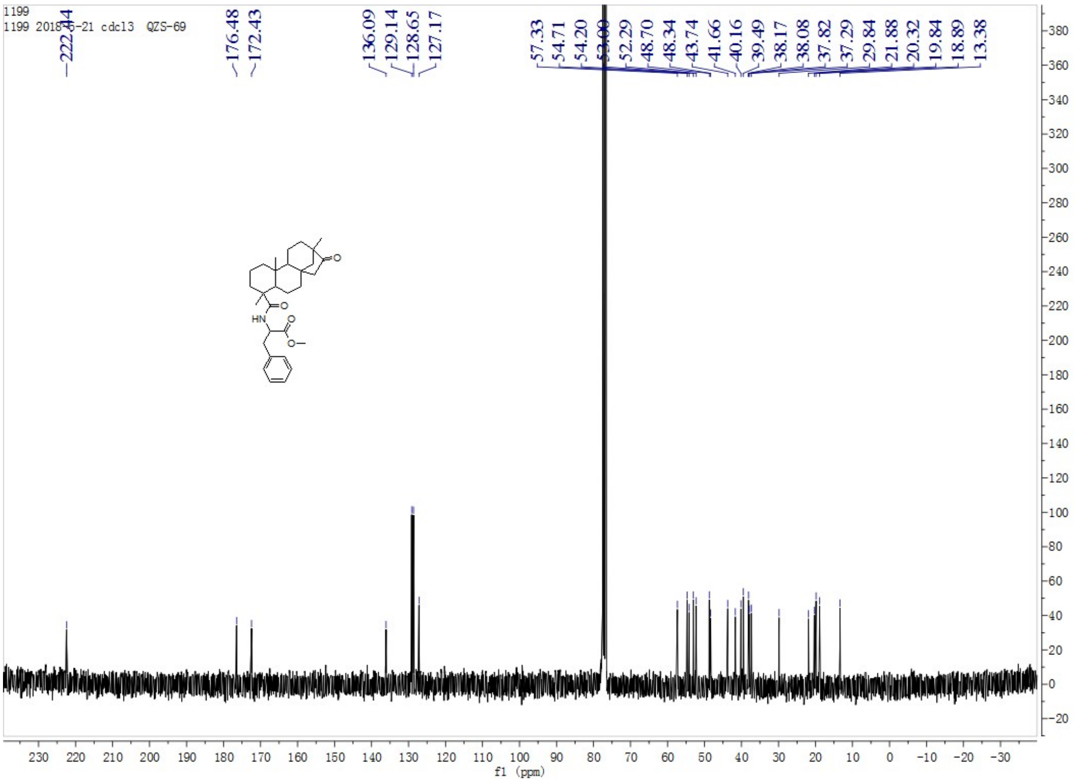

^{1}H NMR and ^{13}C NMR spectra of compound **3b**

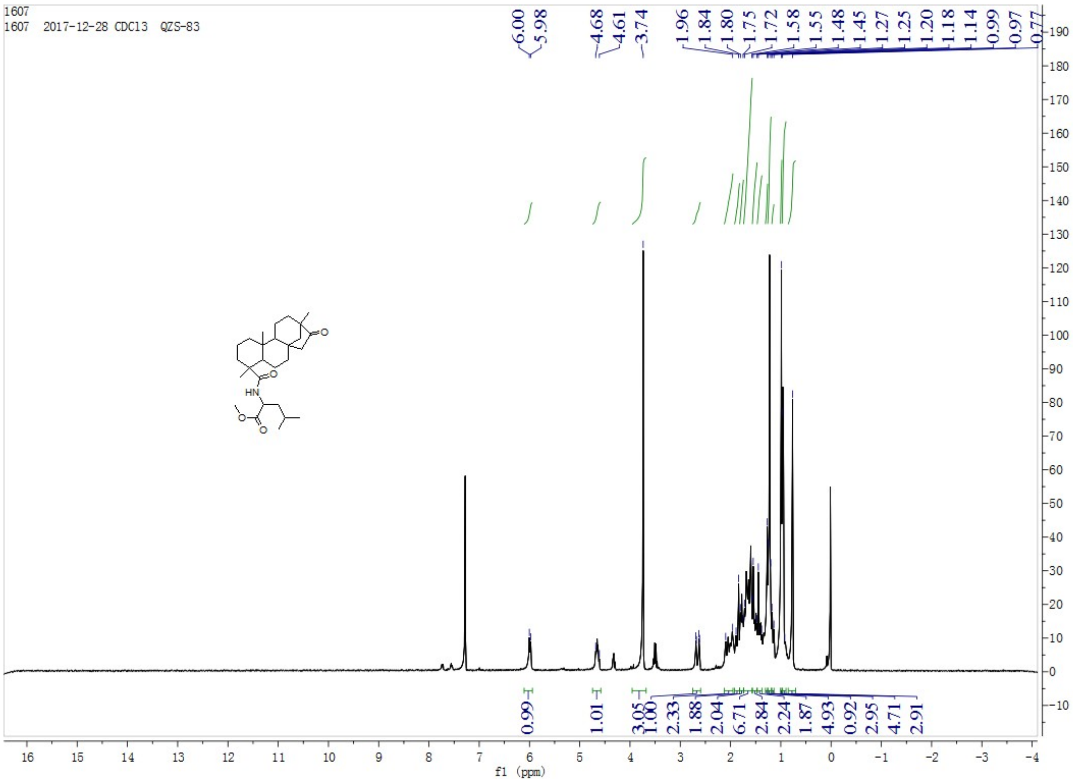

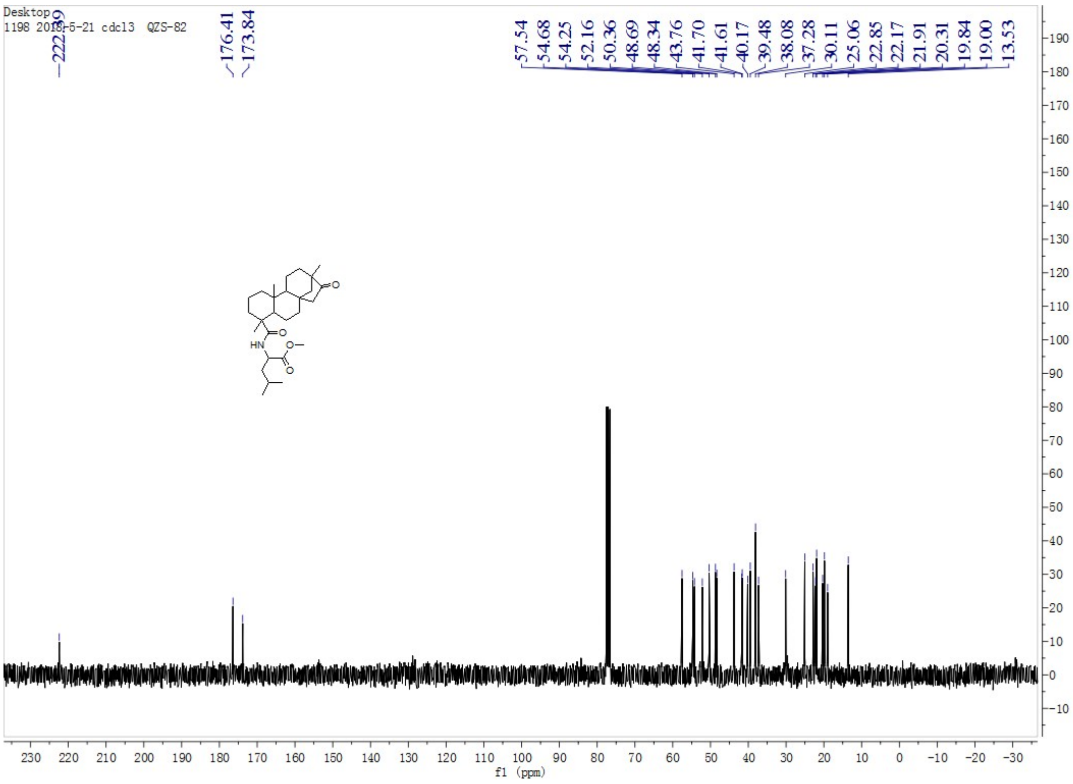

^{1}H NMR and ^{13}C NMR spectra of compound **3c**

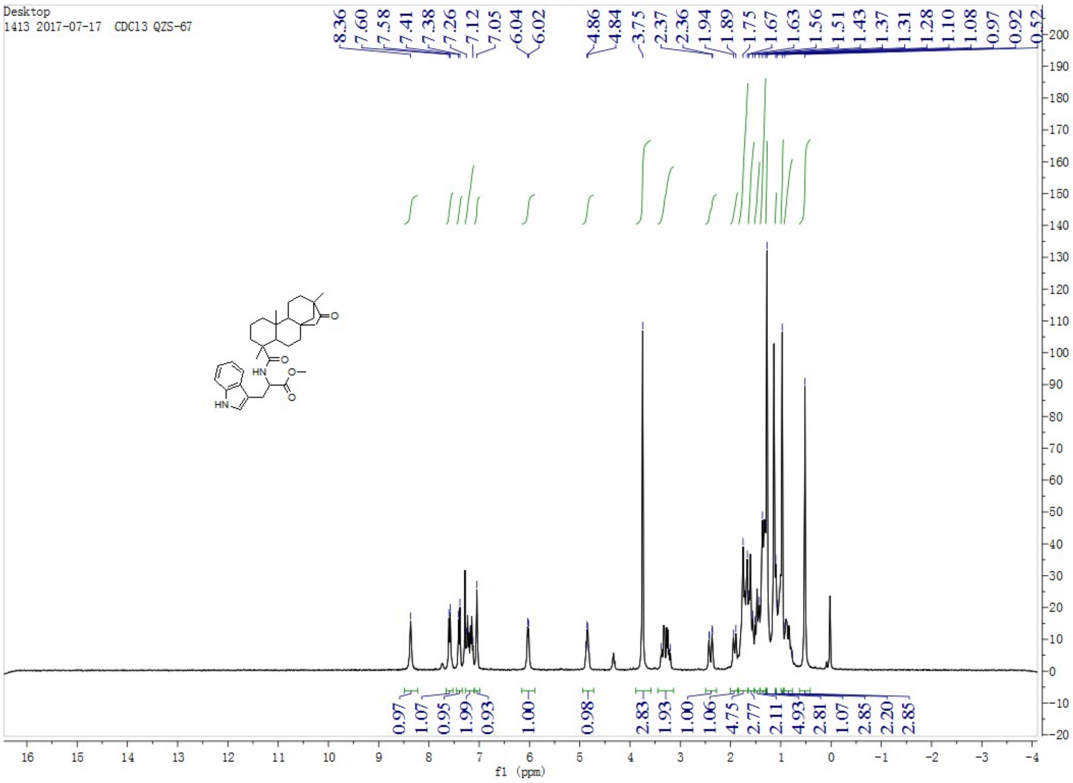

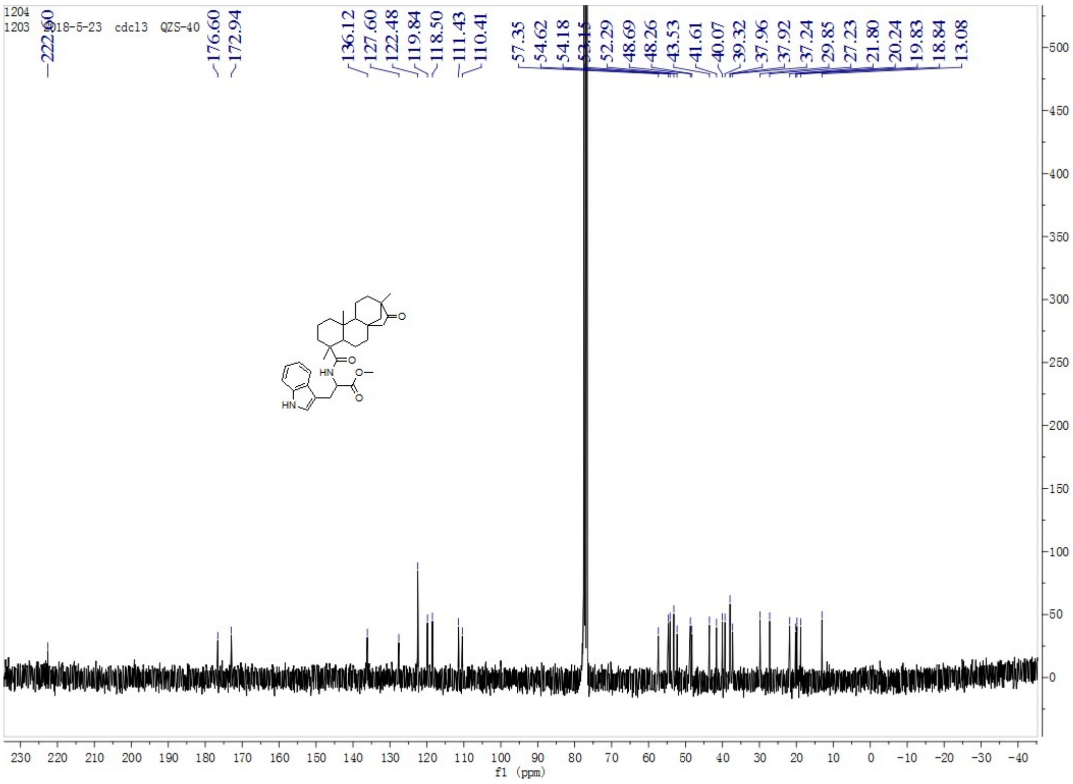

^{1}H NMR and ^{13}C NMR spectra of compound **3d**

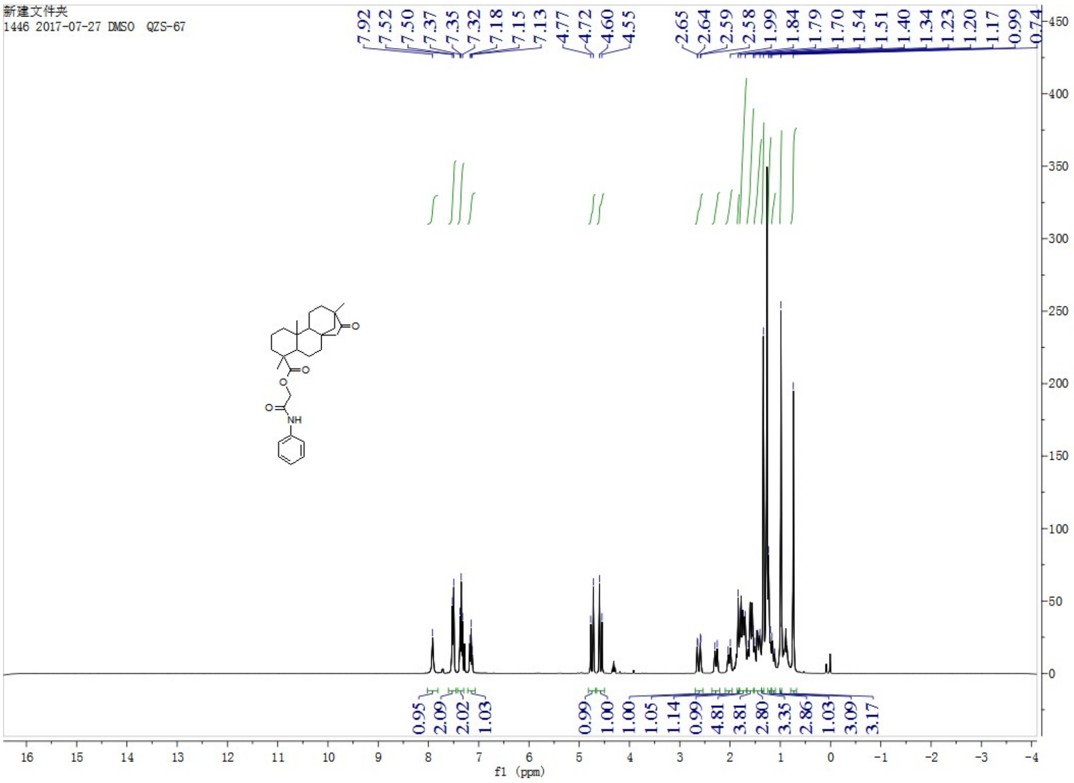

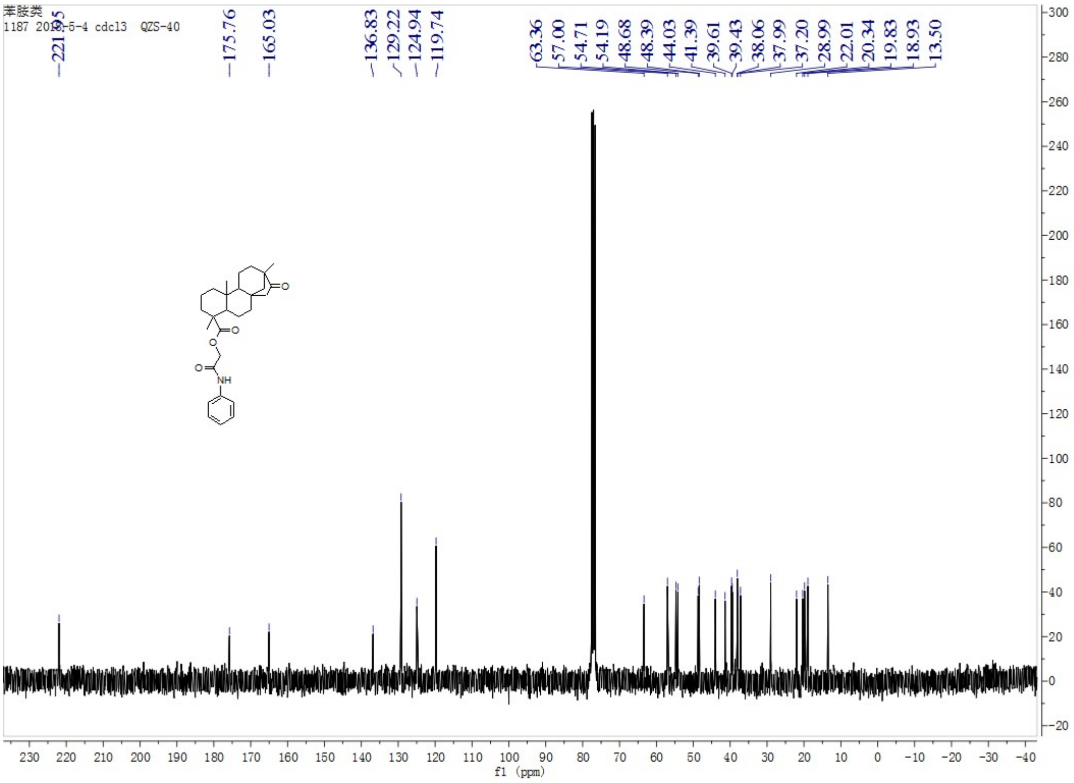

^{1}H NMR and ^{13}C NMR spectra of compound **4a**

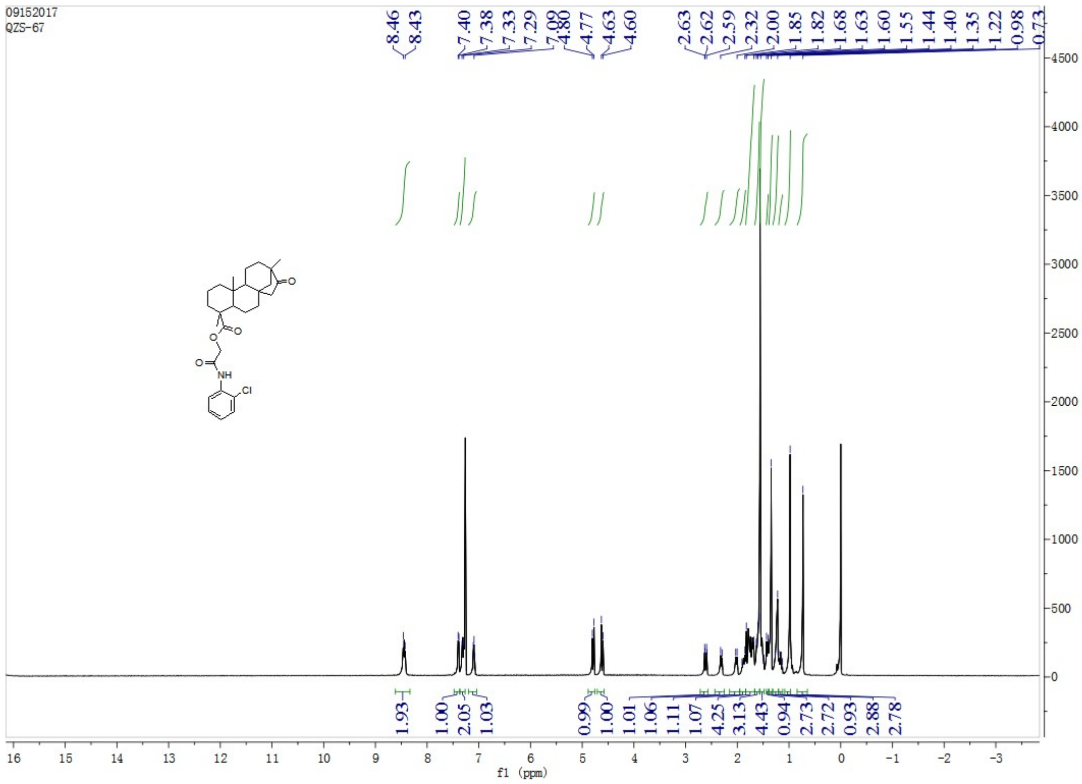

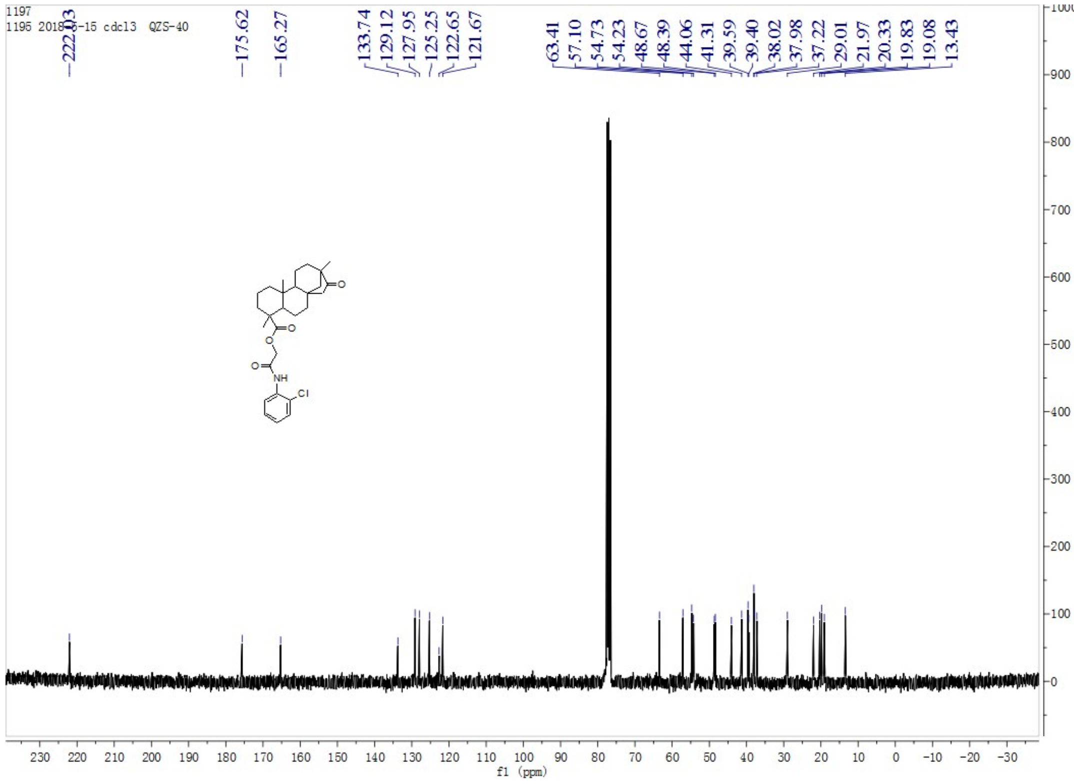

^{1}H NMR and ^{13}C NMR spectra of compound **4b**

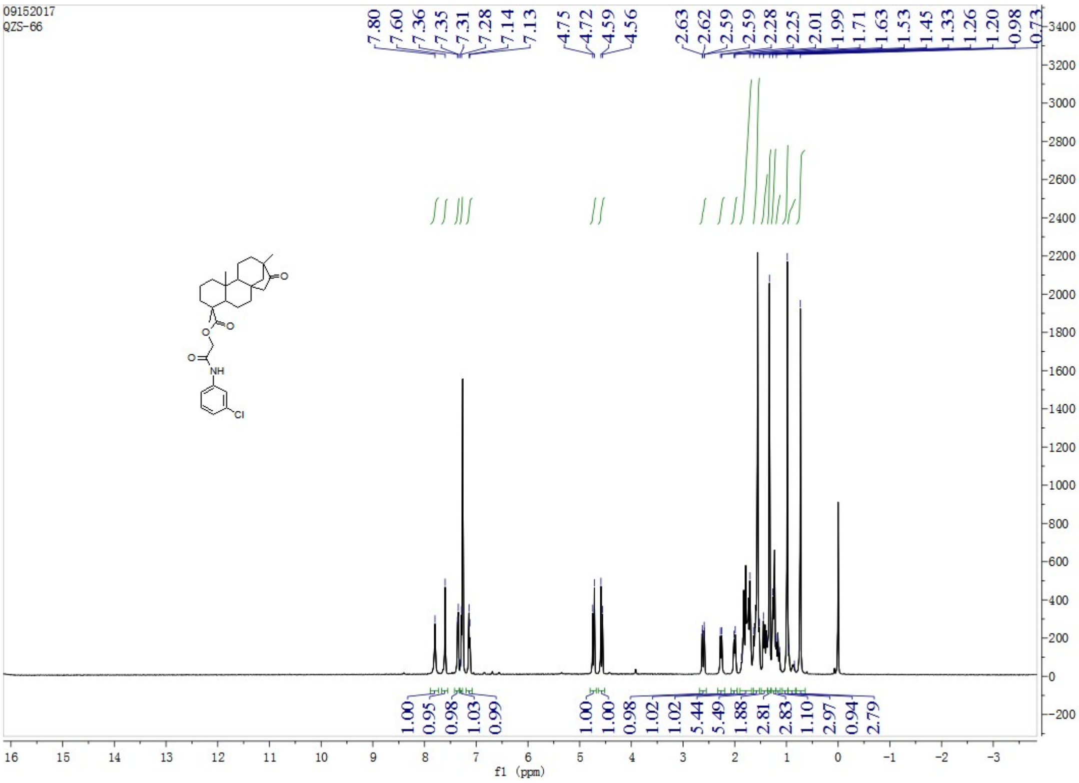

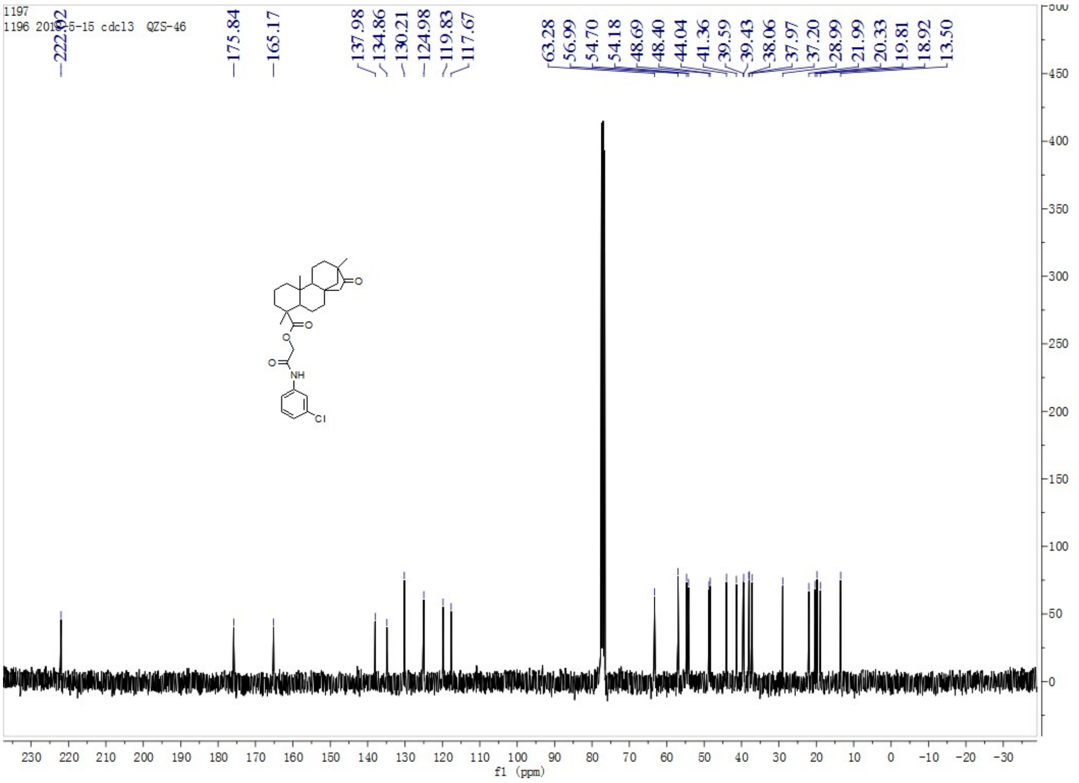

^{1}H NMR and ^{13}C NMR spectra of compound **4c**

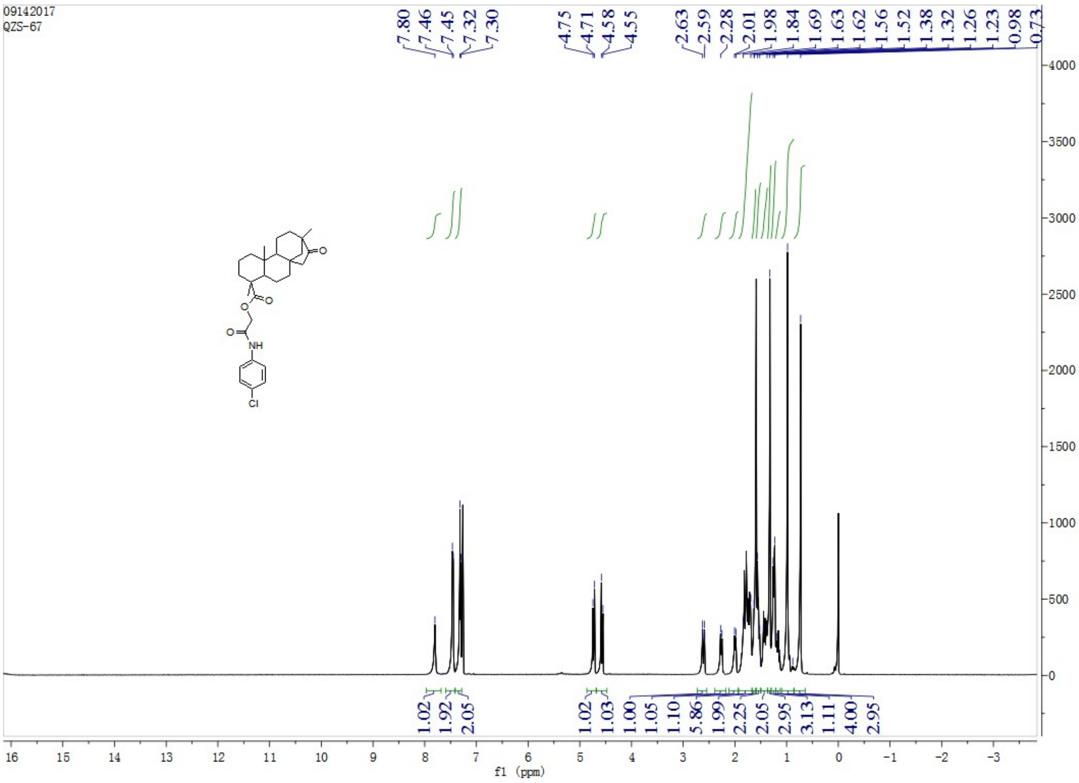

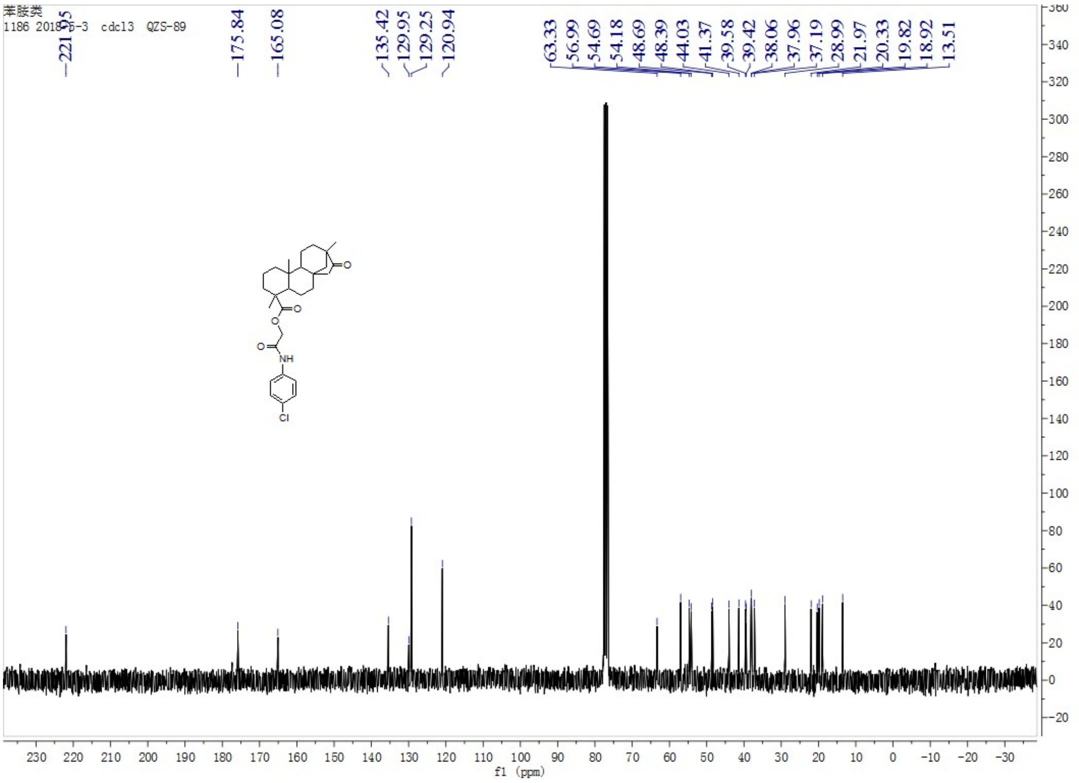

^{1}H NMR and ^{13}C NMR spectra of compound **4d**

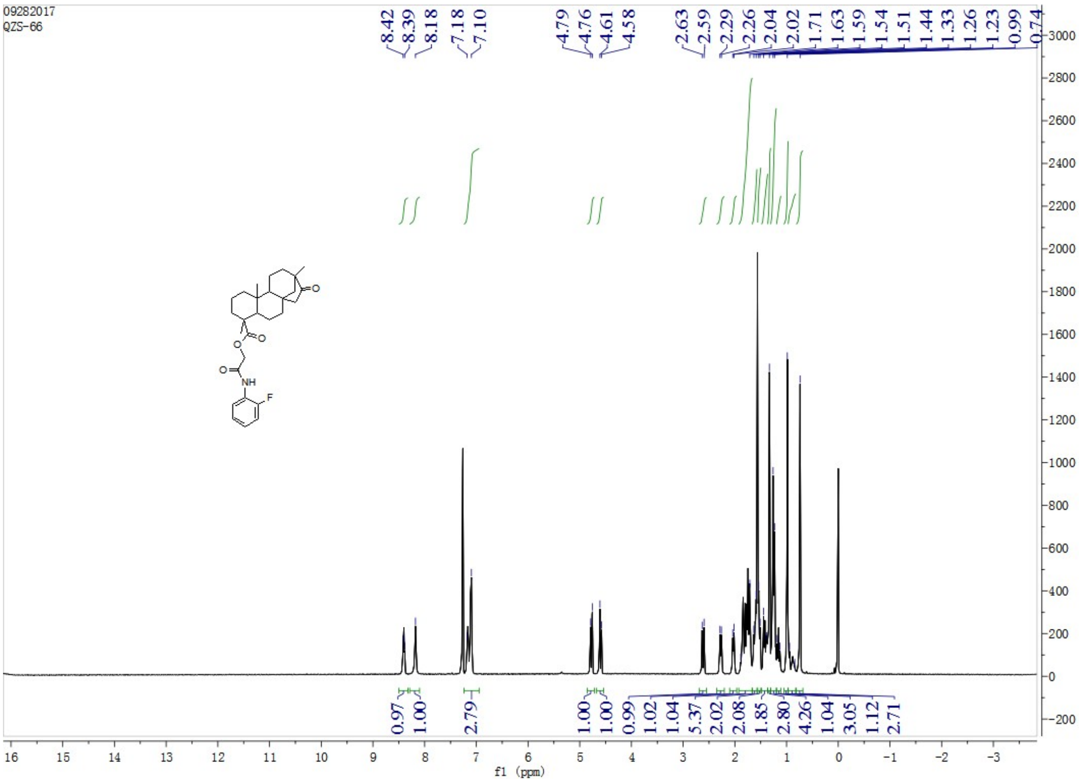

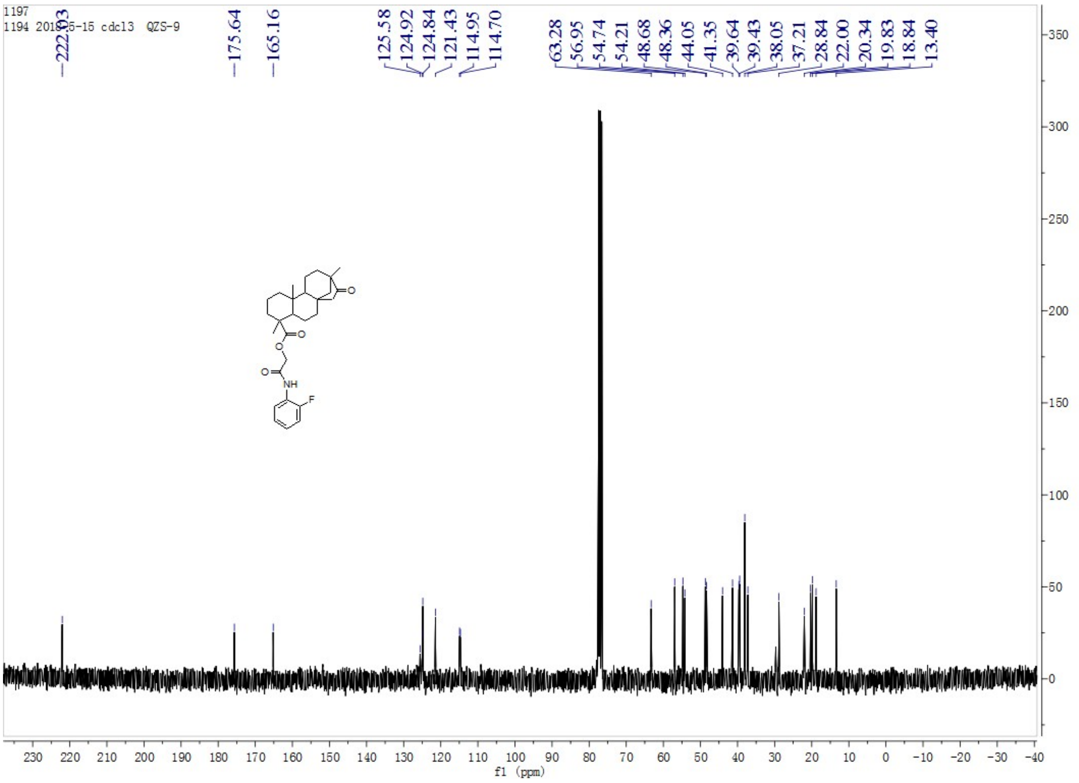

^{1}H NMR and ^{13}C NMR spectra of compound **4e**

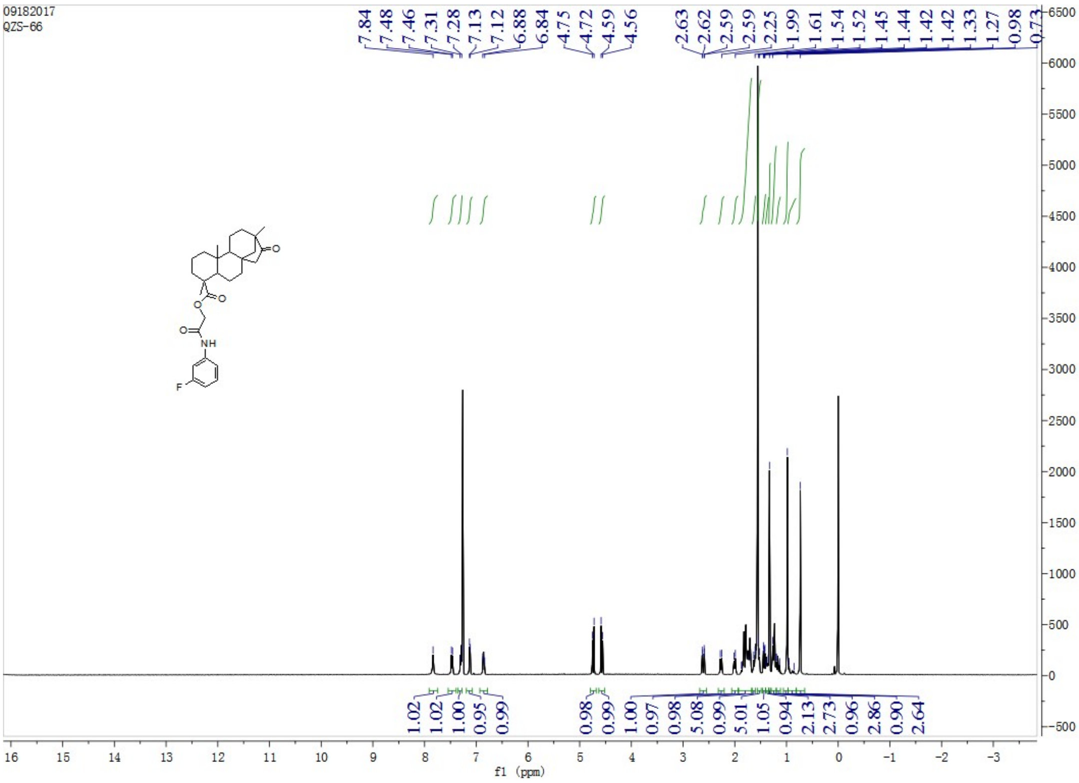

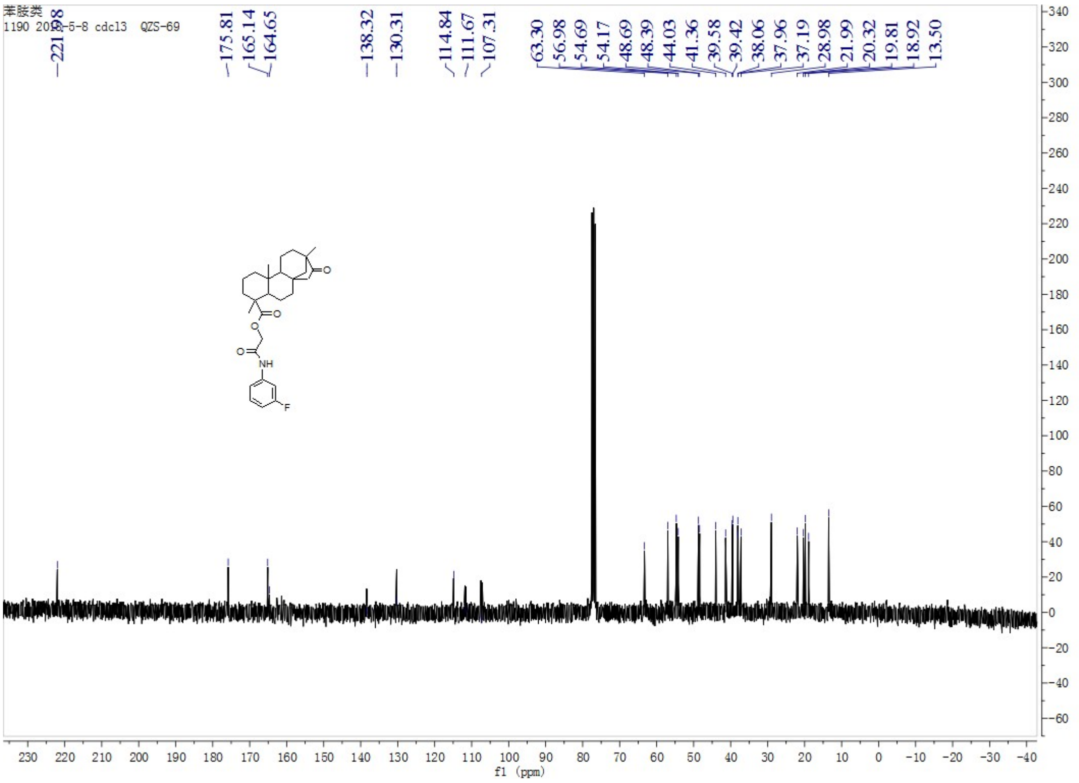

^{1}H NMR and ^{13}C NMR spectra of compound **4f**

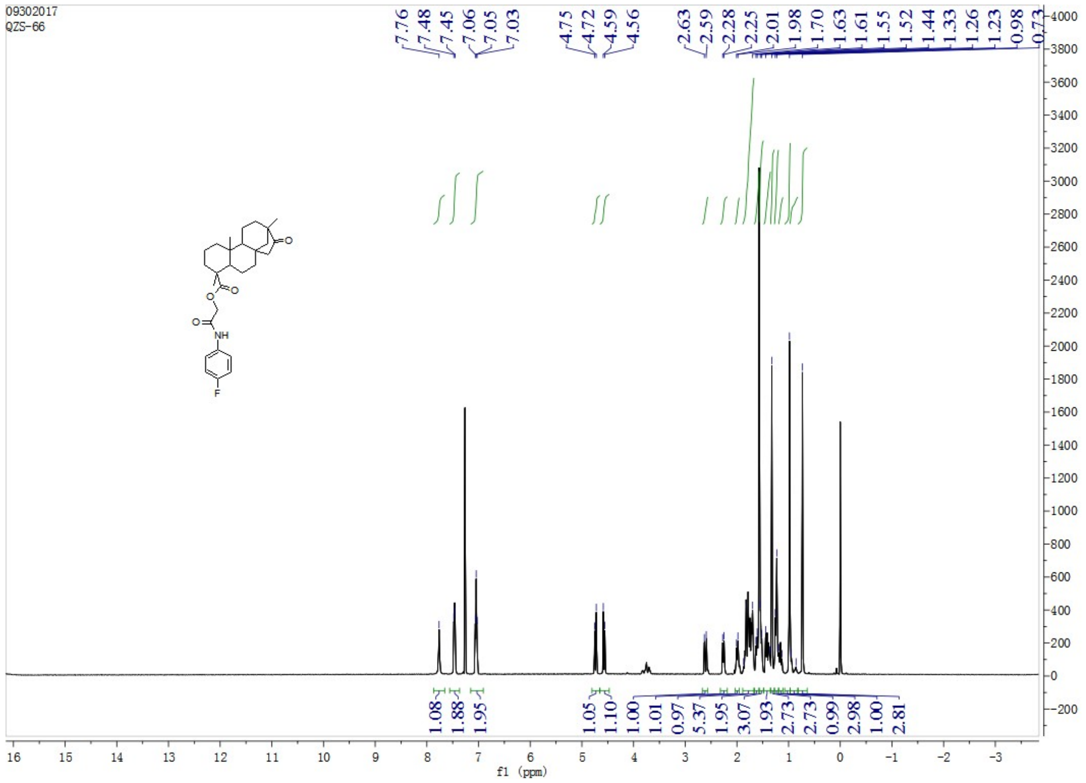

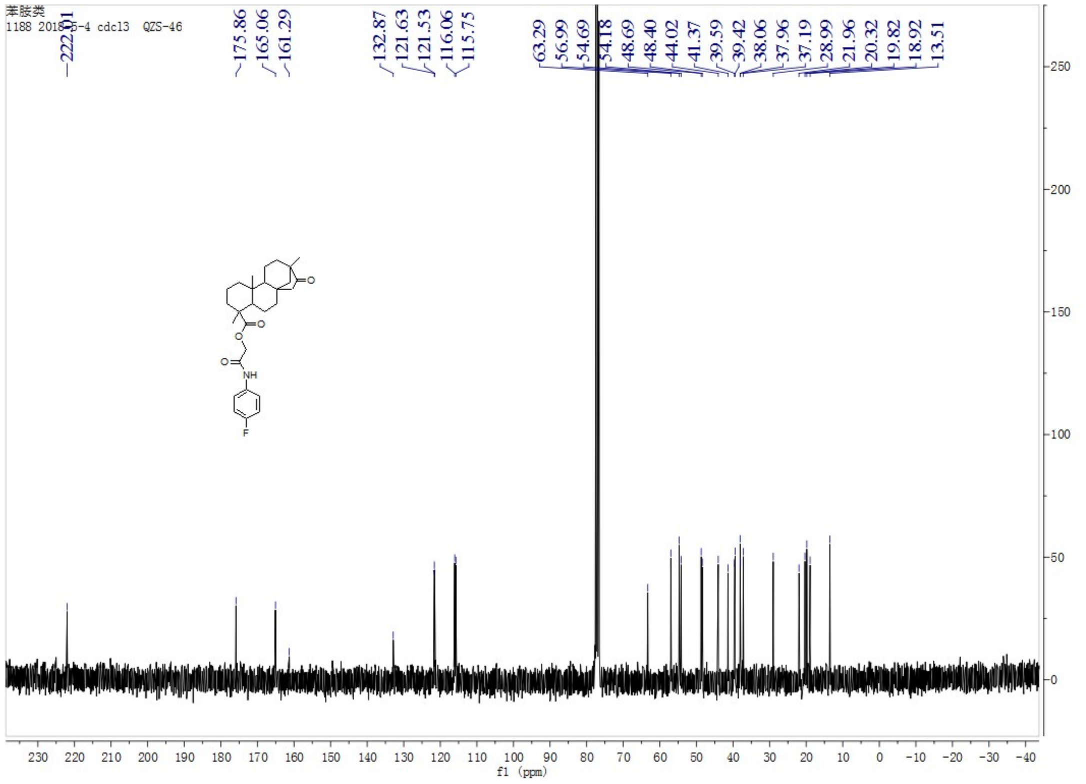

^{1}H NMR and ^{13}C NMR spectra of compound **4g**

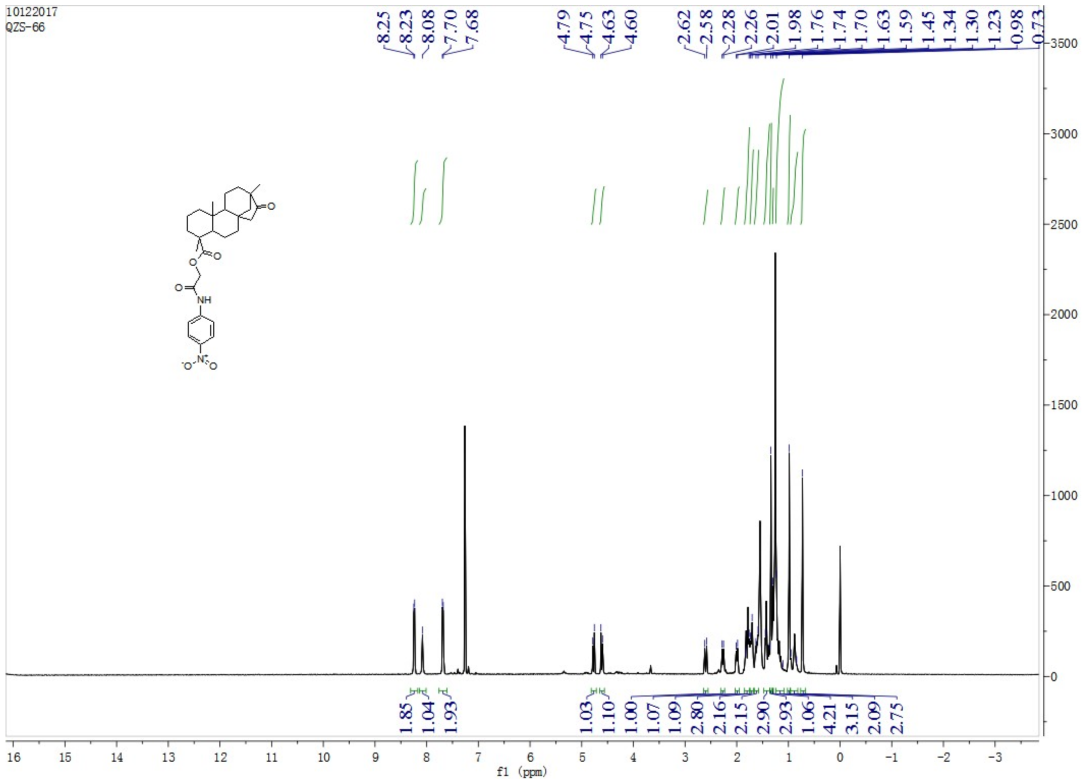

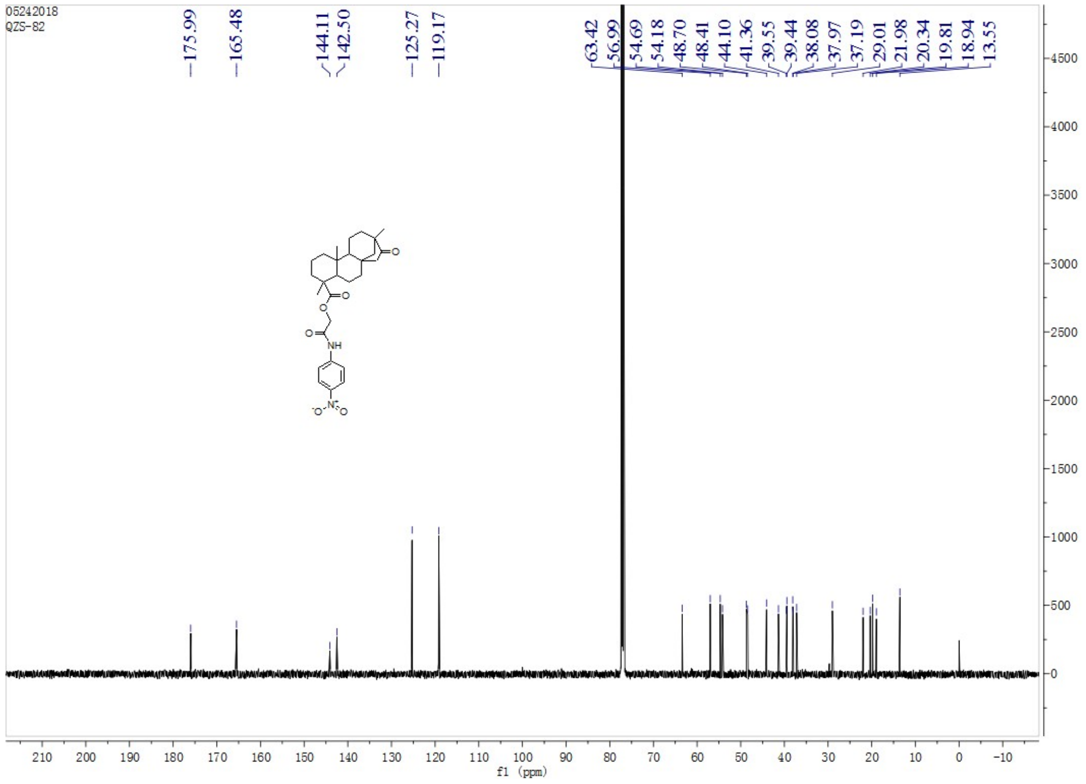

^{1}H NMR and ^{13}C NMR spectra of compound **4h**

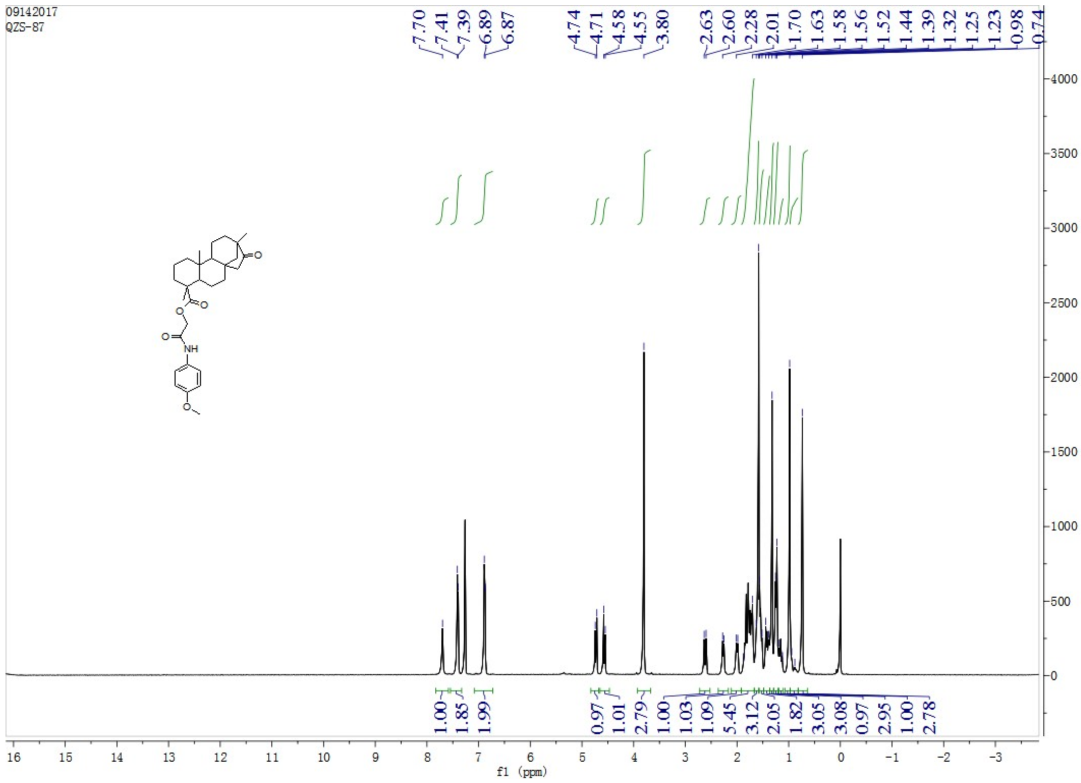

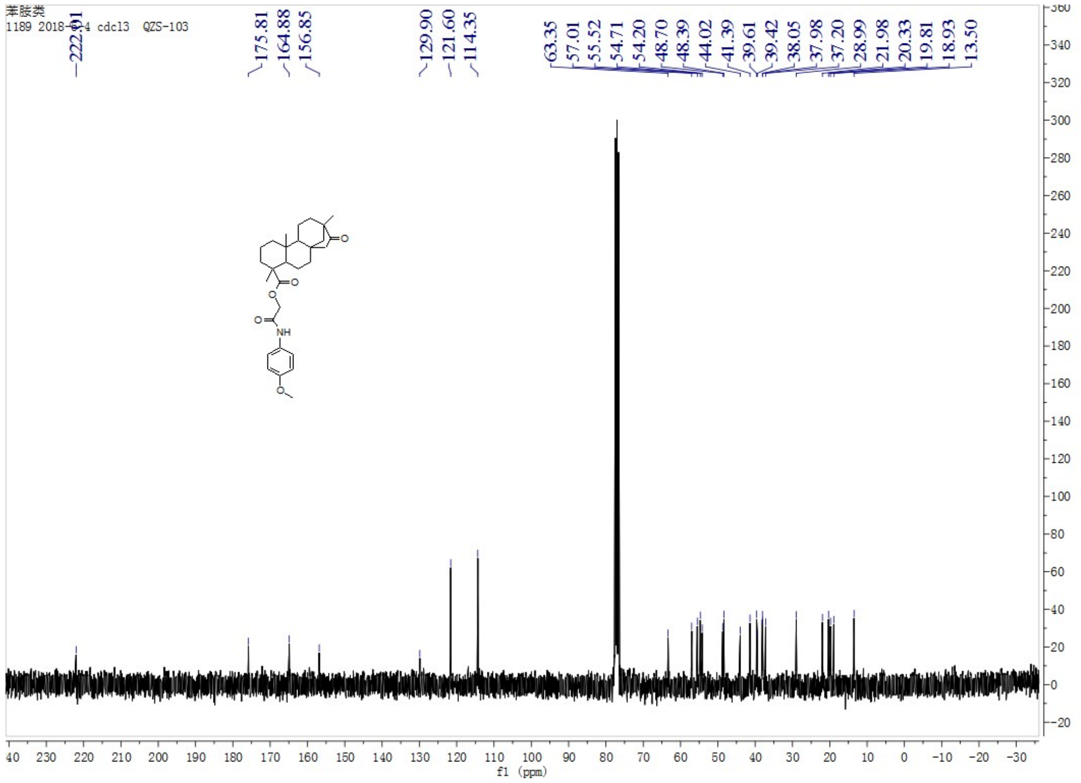

^{1}H NMR and ^{13}C NMR spectra of compound **4i**

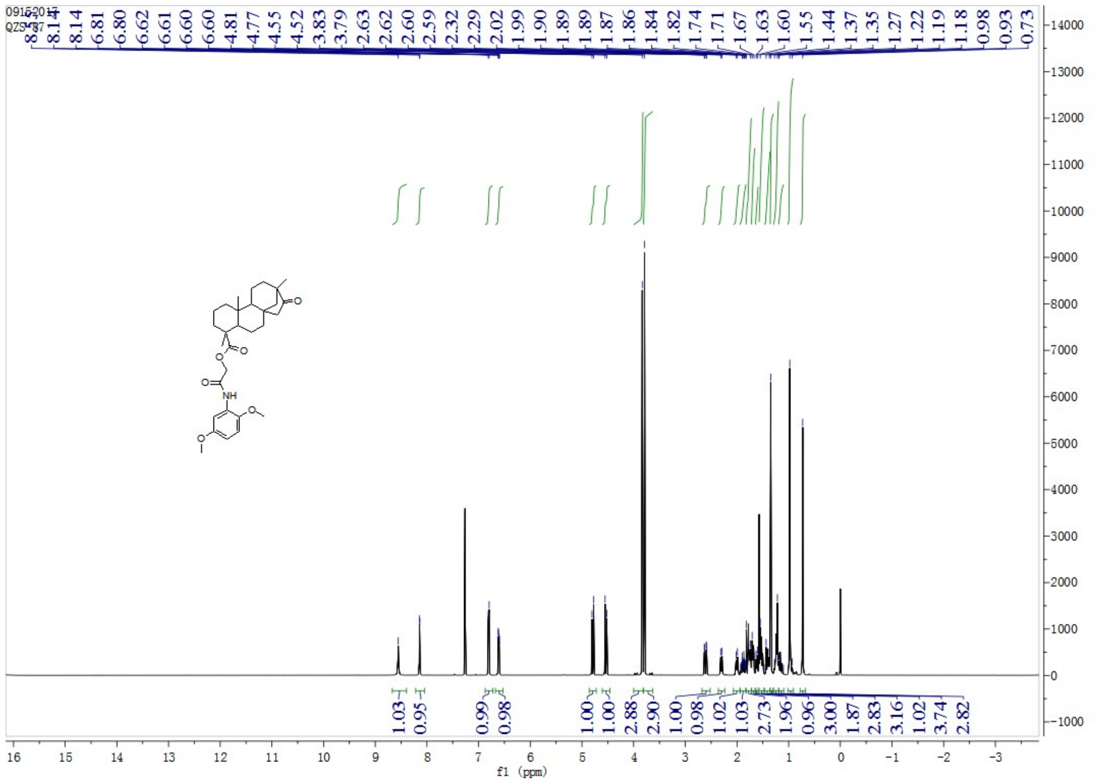

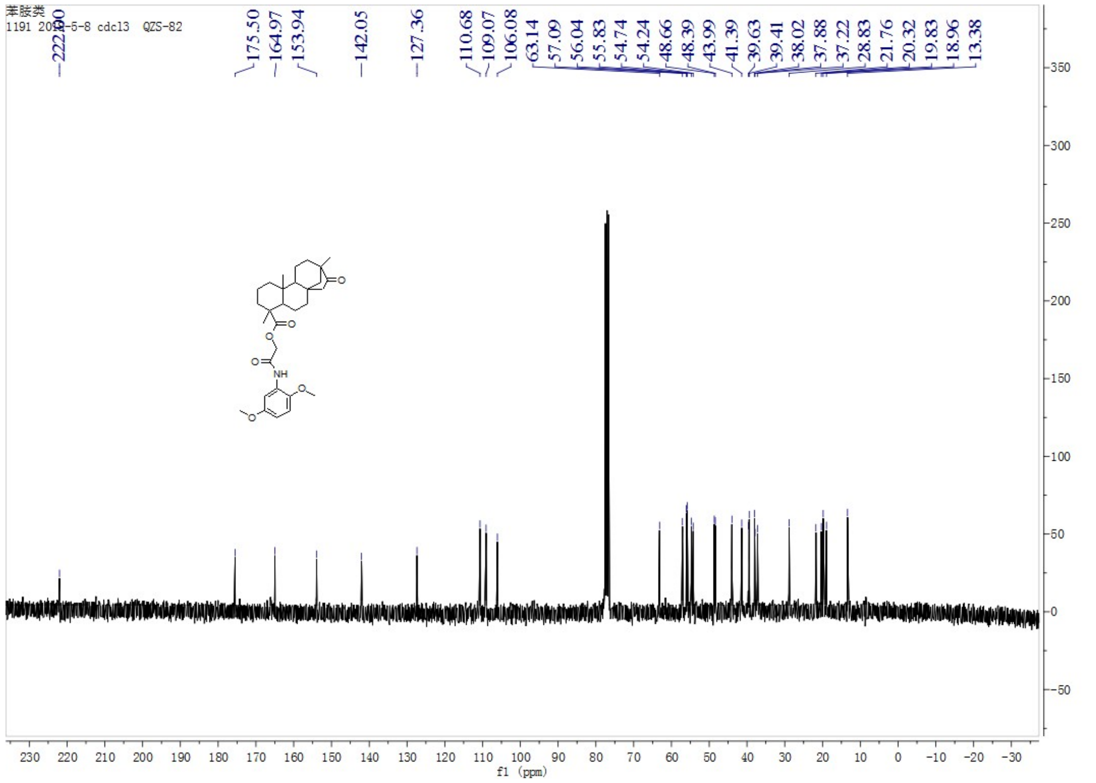

^{1}H NMR and ^{13}C NMR spectra of compound **4j**

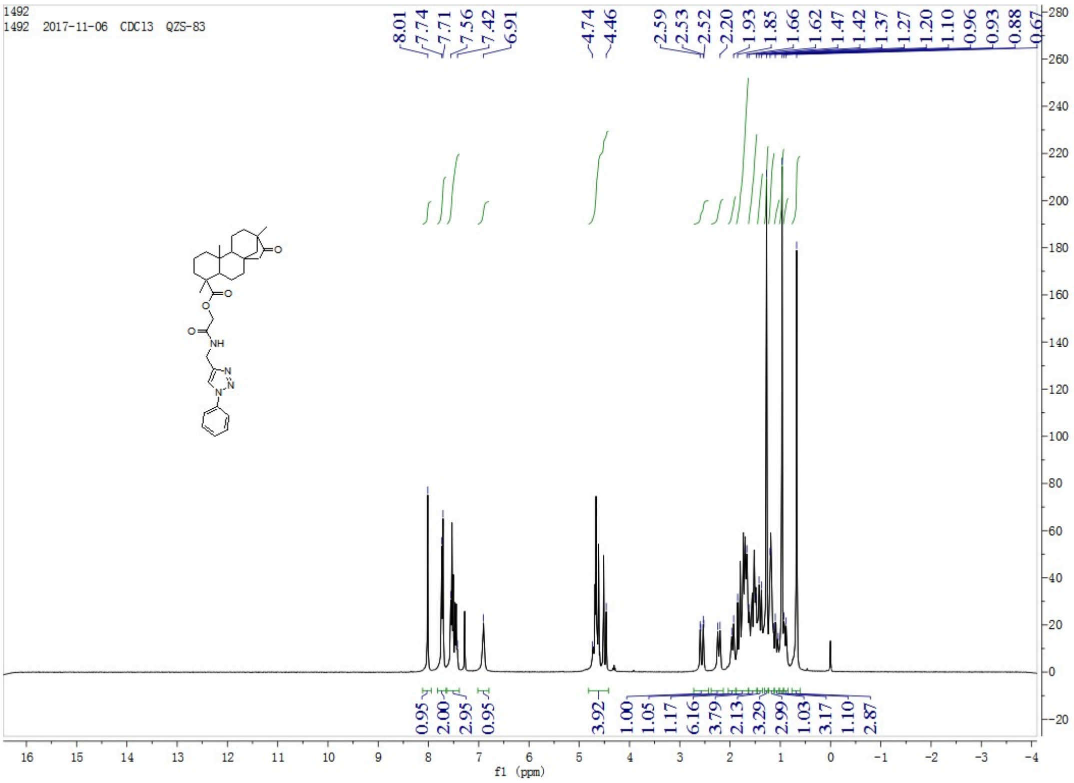

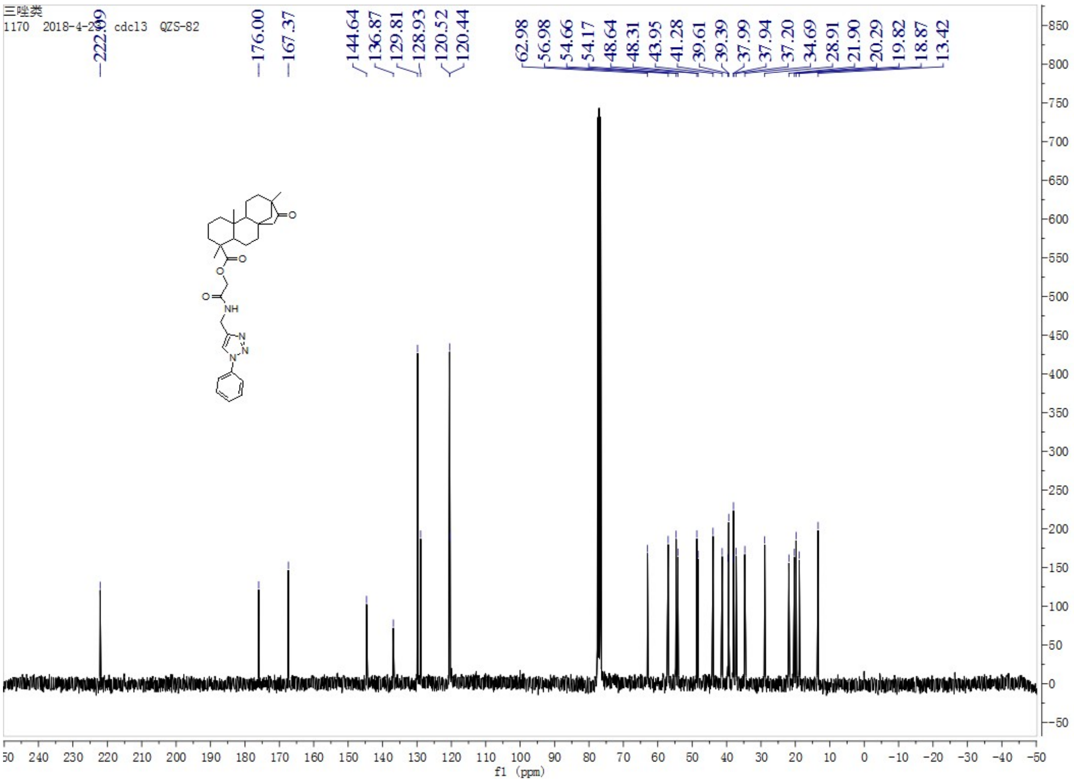

^{1}H NMR and ^{13}C NMR spectra of compound **5a**

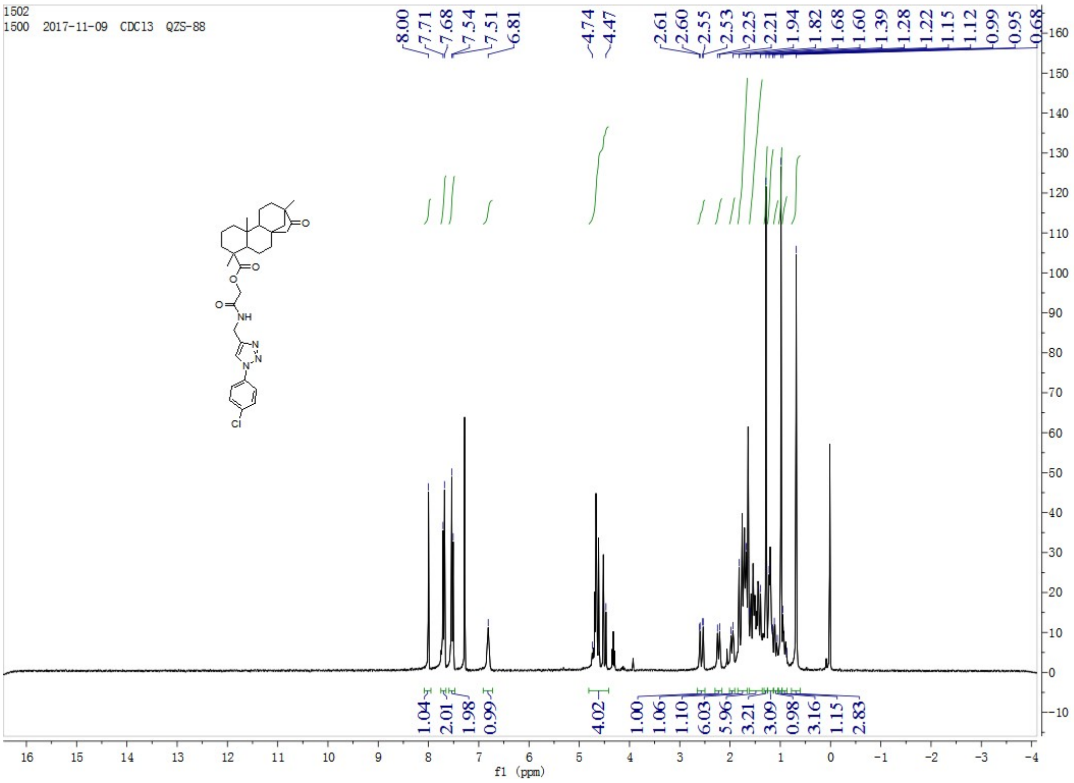

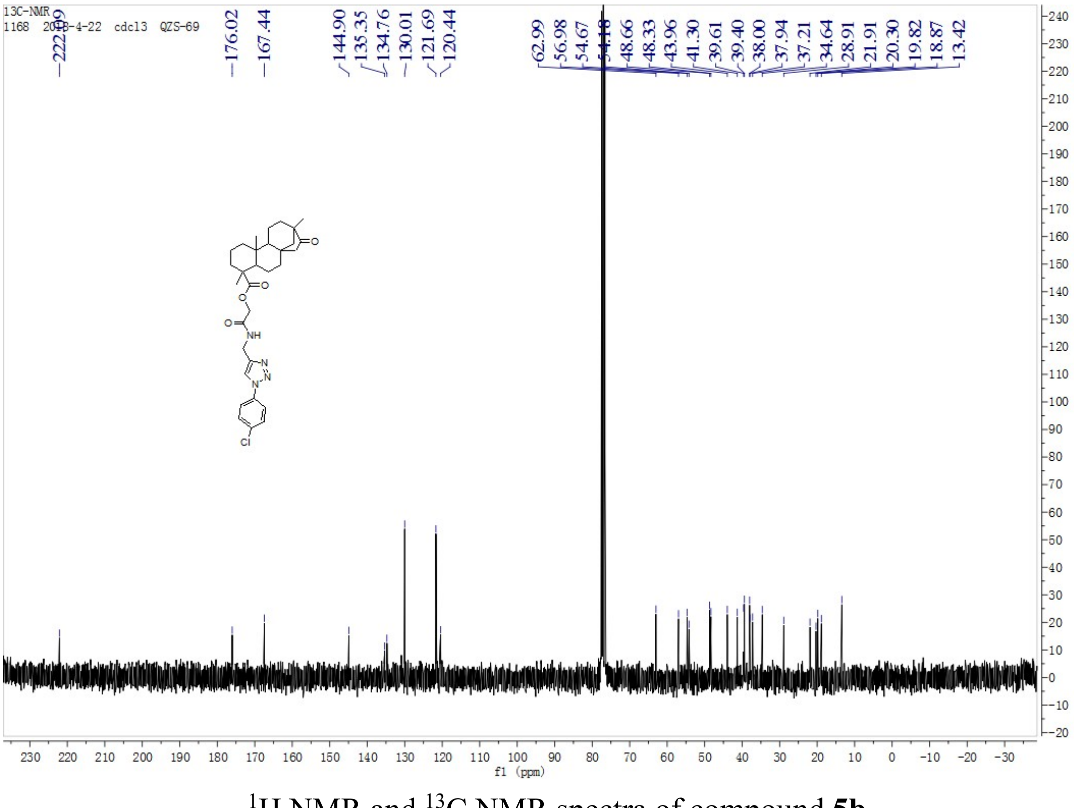

^{1}H NMR and ^{13}C NMR spectra of compound **5b**

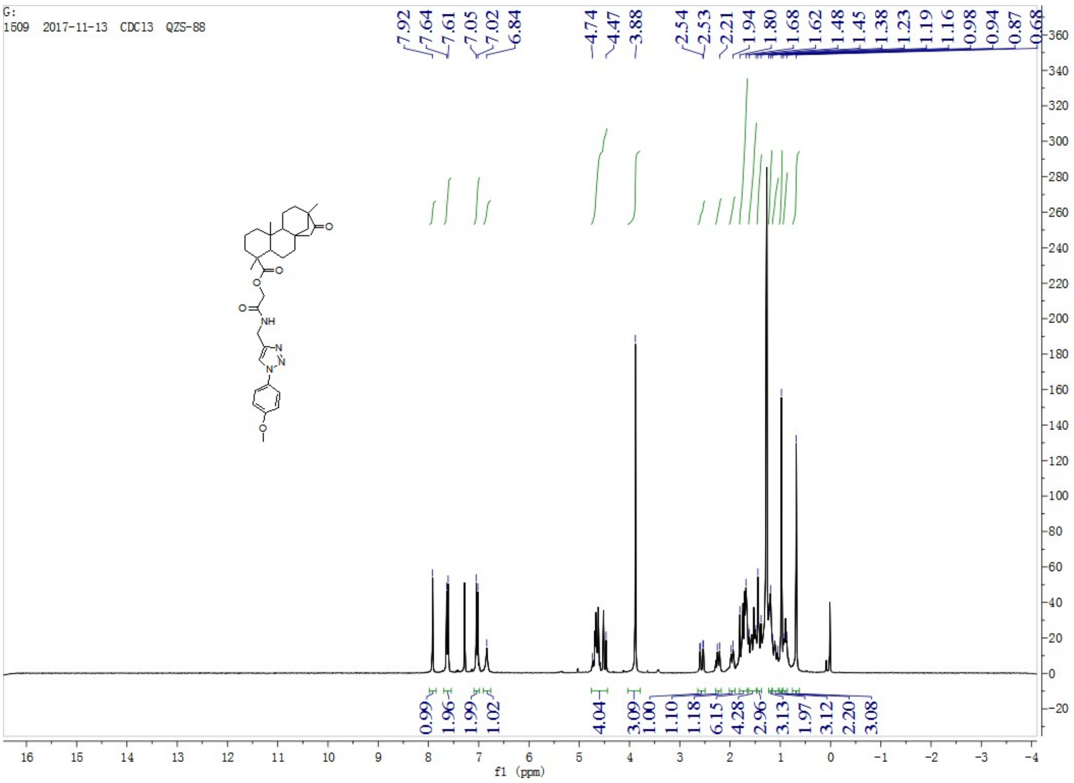

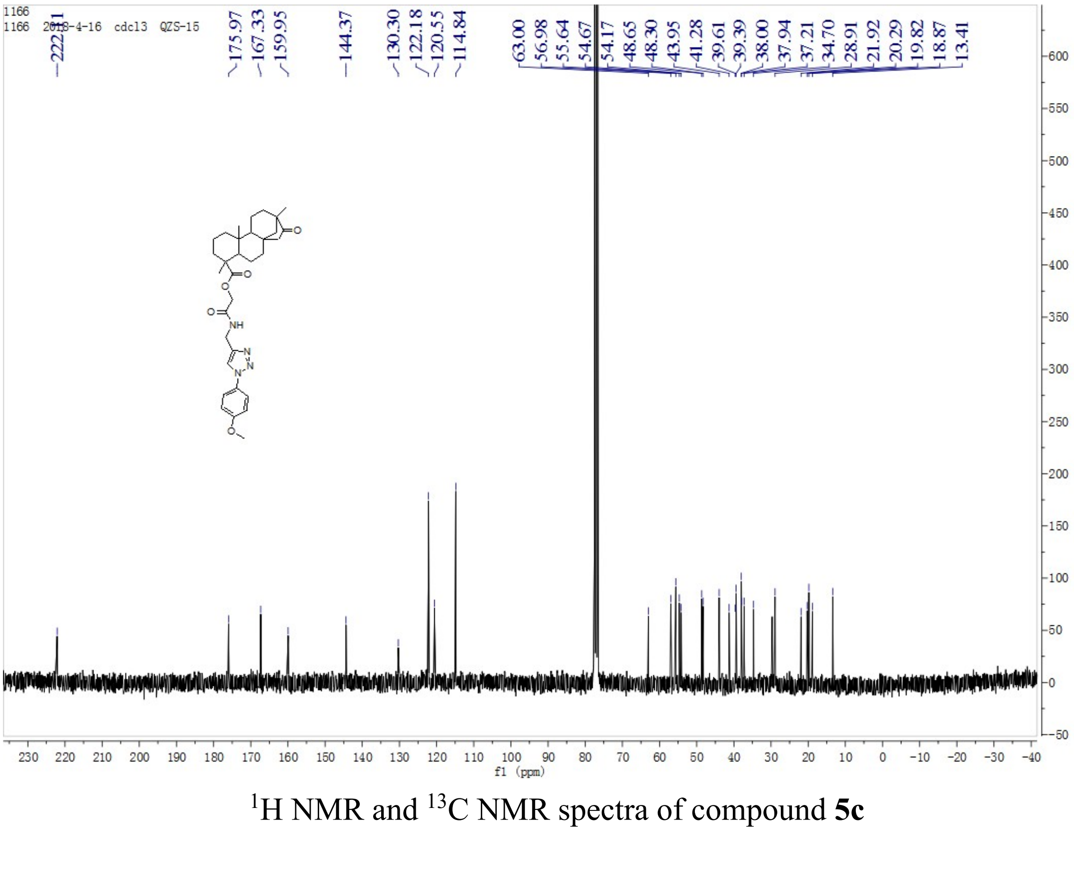

^{1}H NMR and ^{13}C NMR spectra of compound **5c**

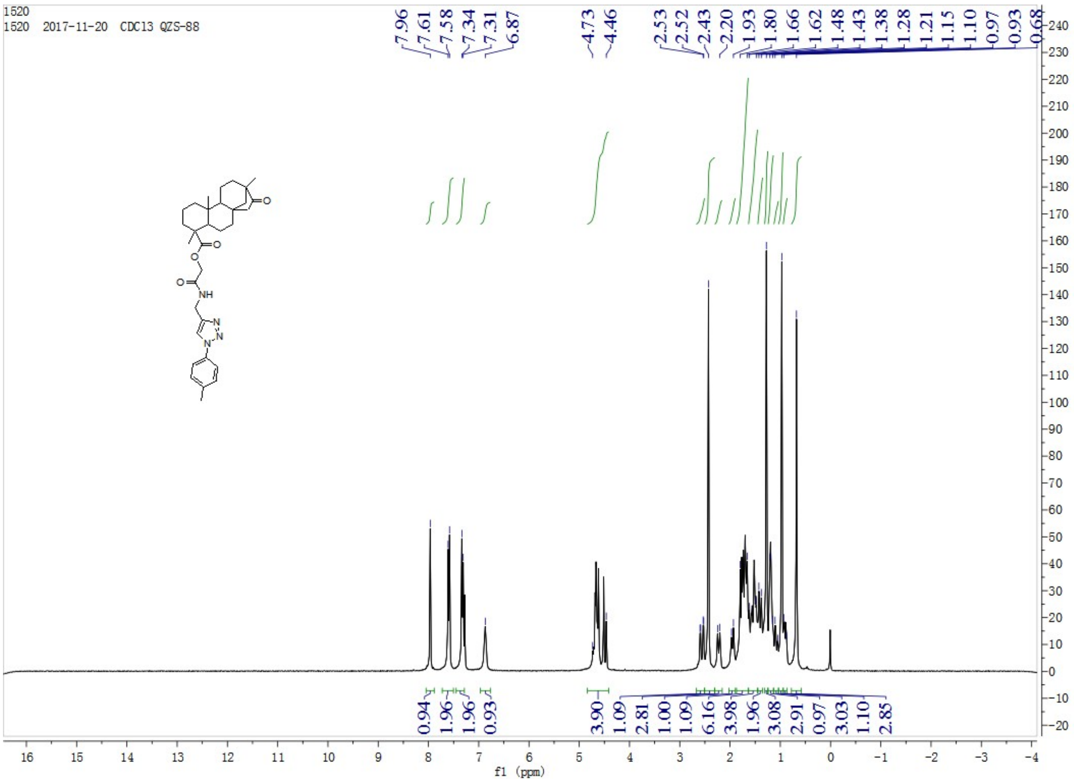

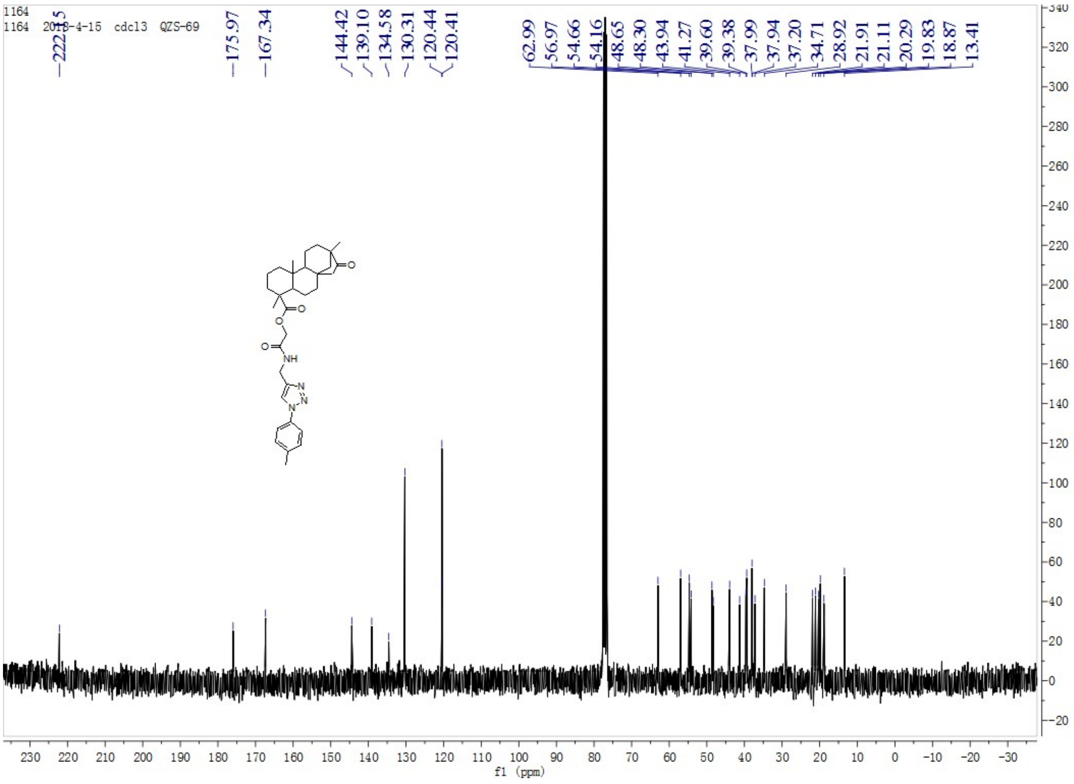

^{1}H NMR and ^{13}C NMR spectra of compound **5d**

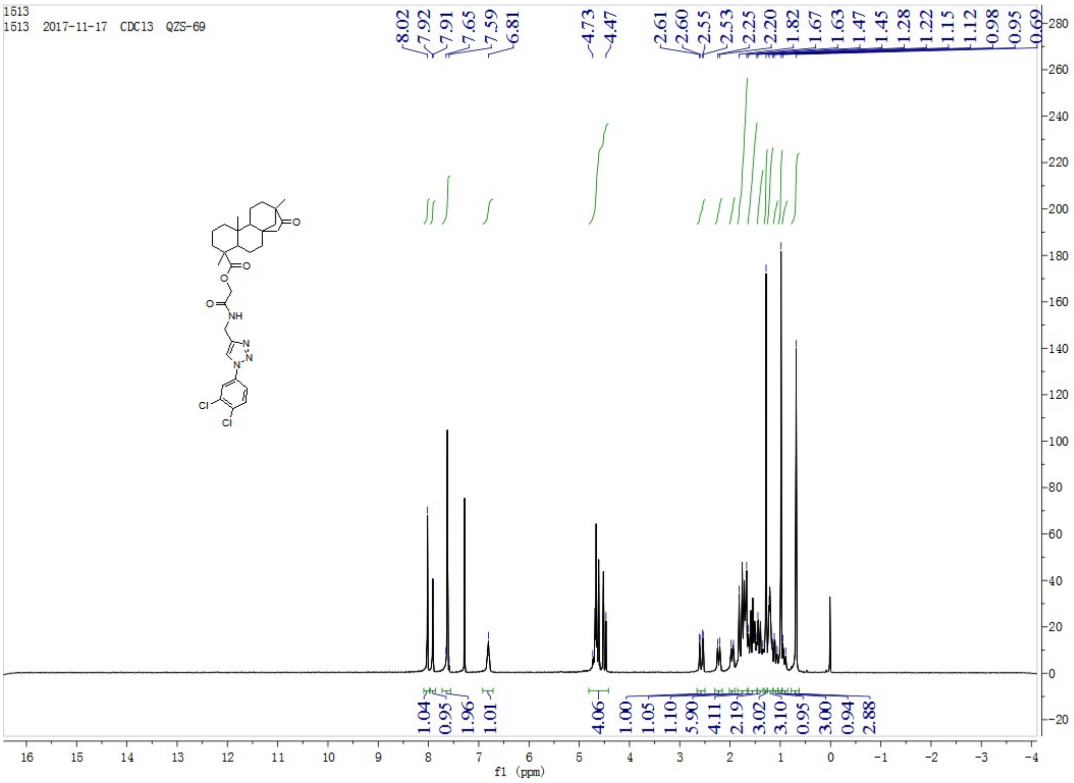

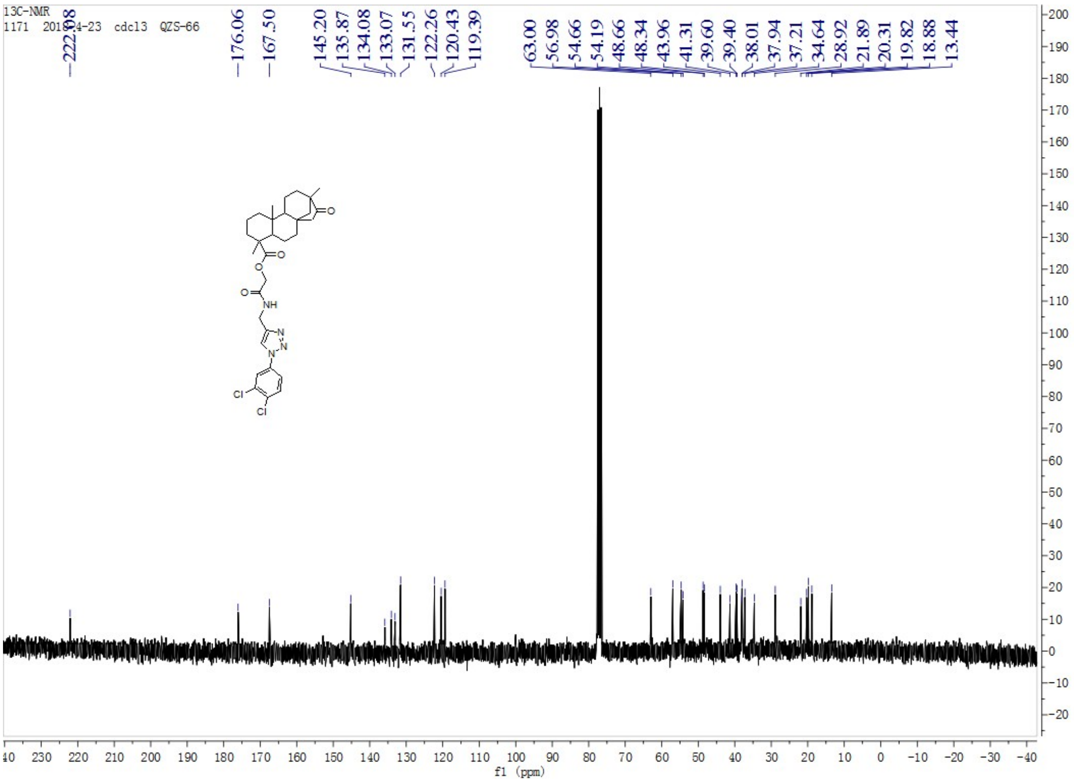

^{1}H NMR and ^{13}C NMR spectra of compound **5e**

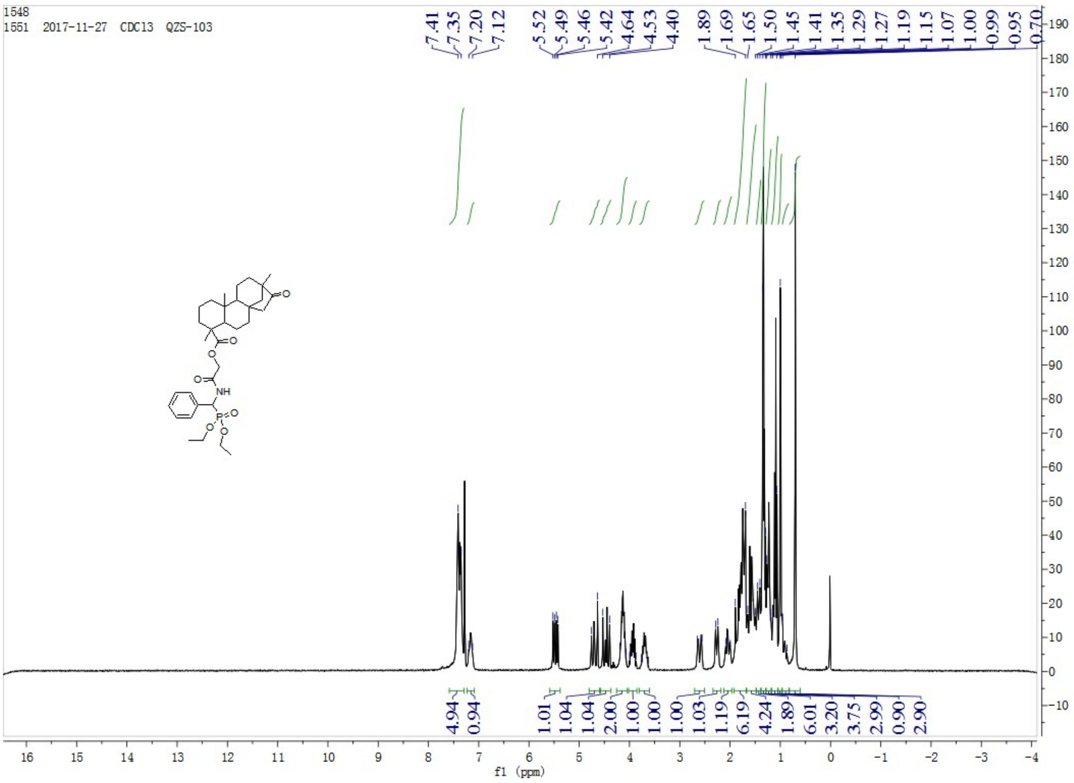

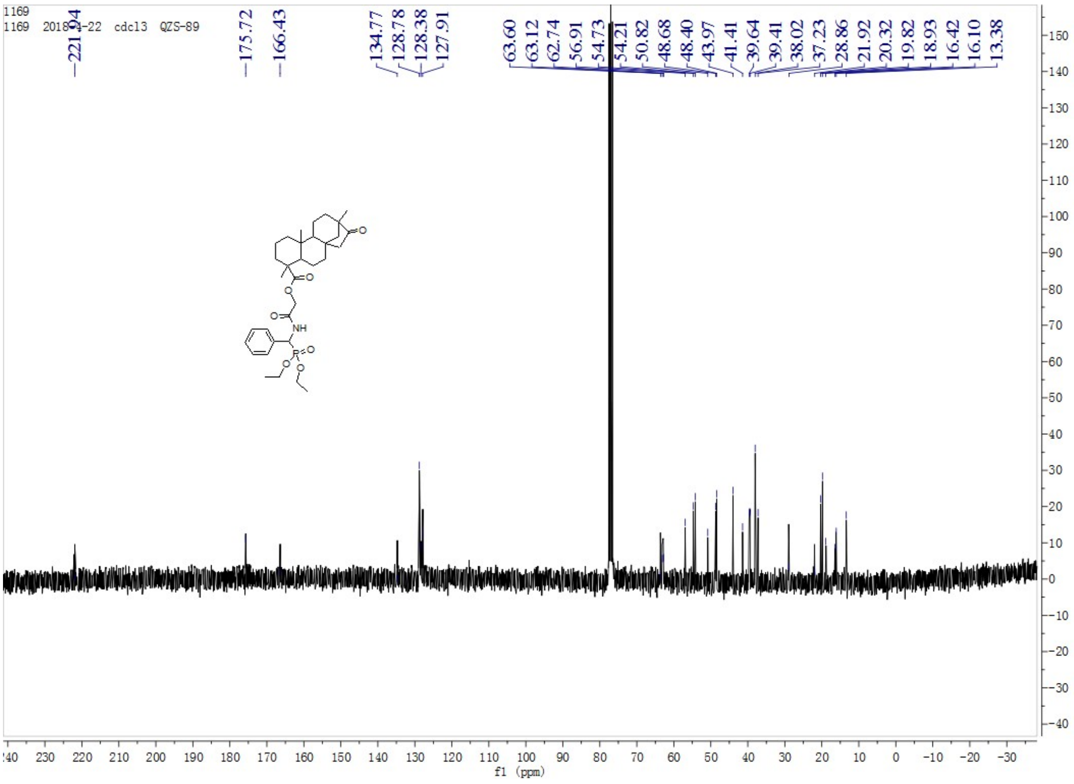

^{1}H NMR and ^{13}C NMR spectra of compound **6a**

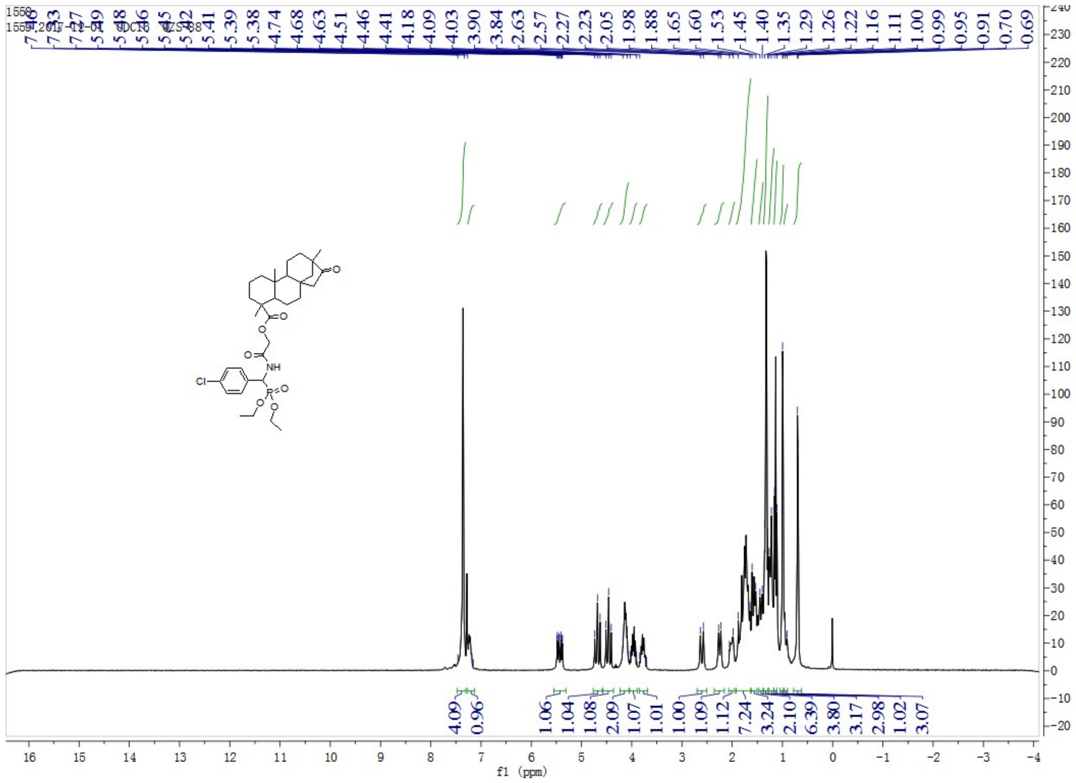

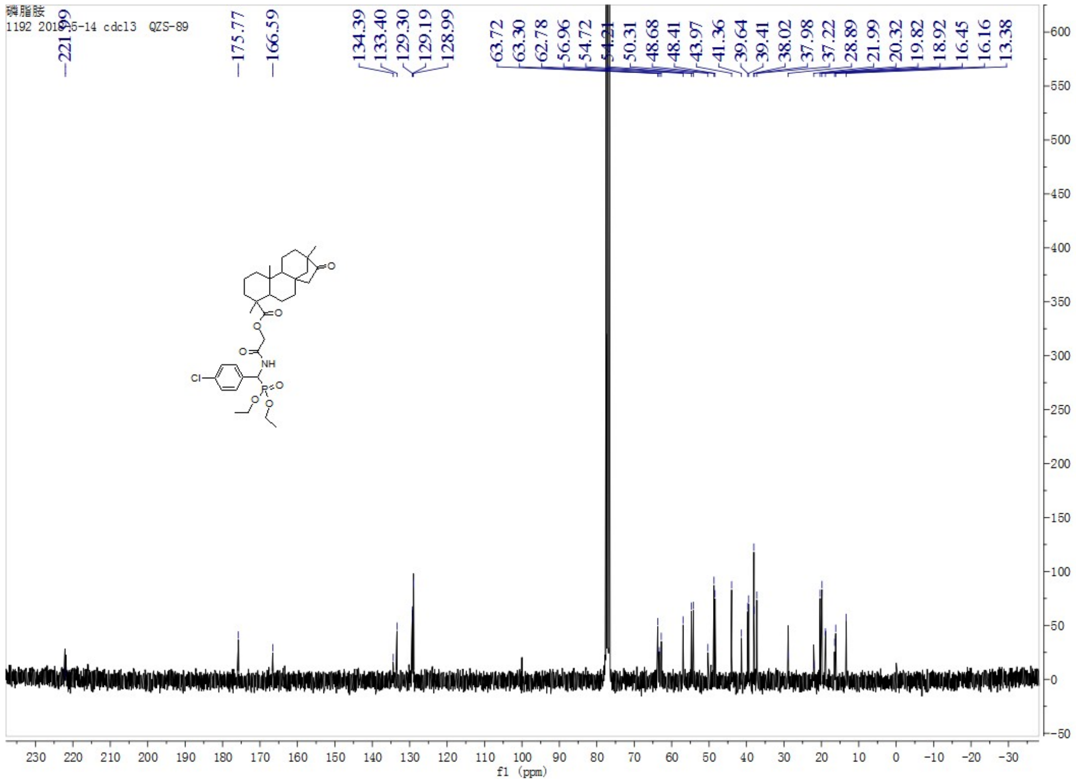

^{1}H NMR and ^{13}C NMR spectra of compound **6b**

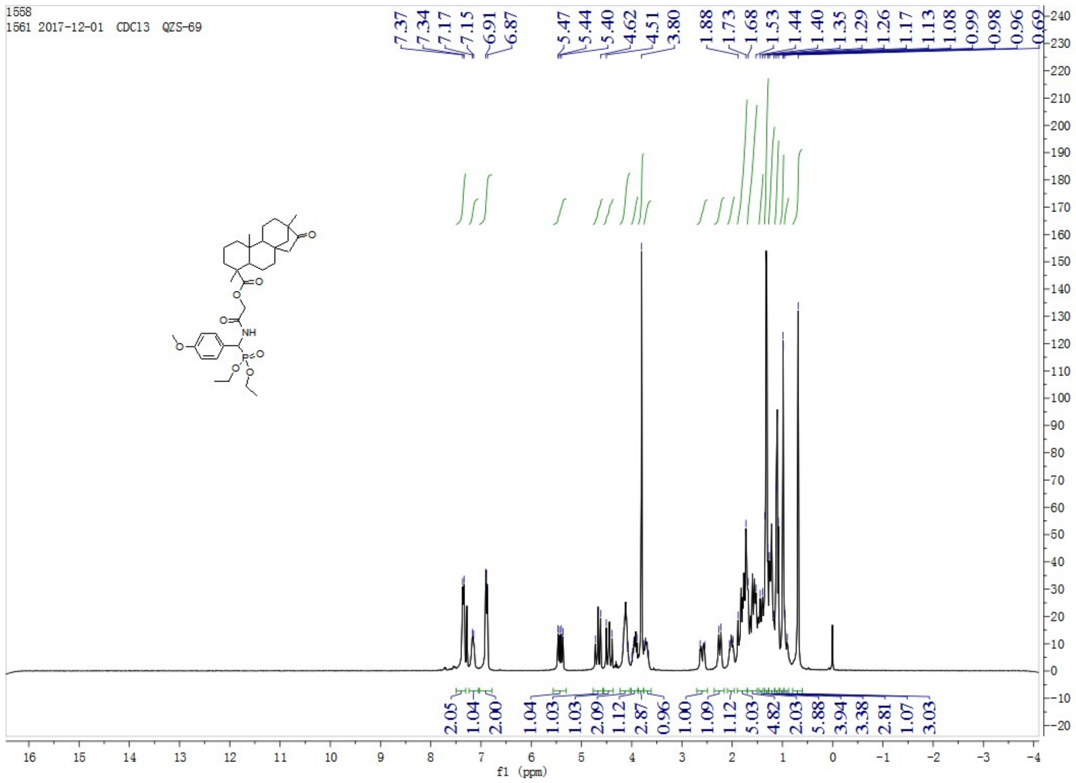

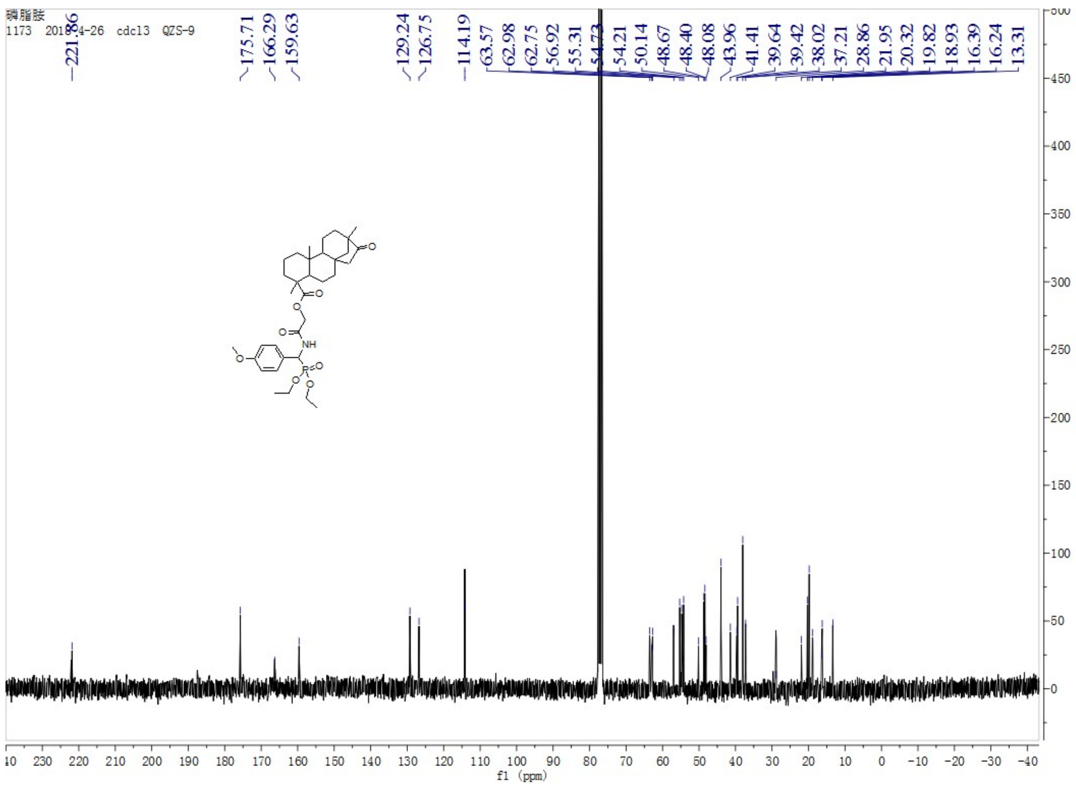

^{1}H NMR and ^{13}C NMR spectra of compound **6c**

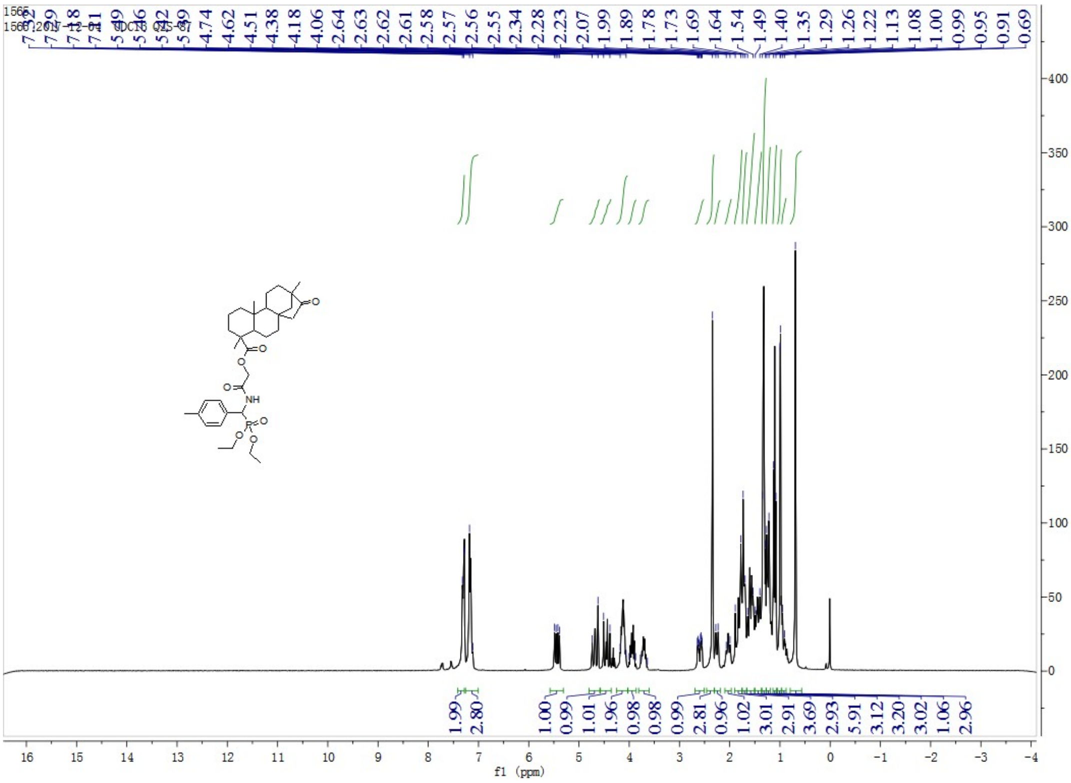

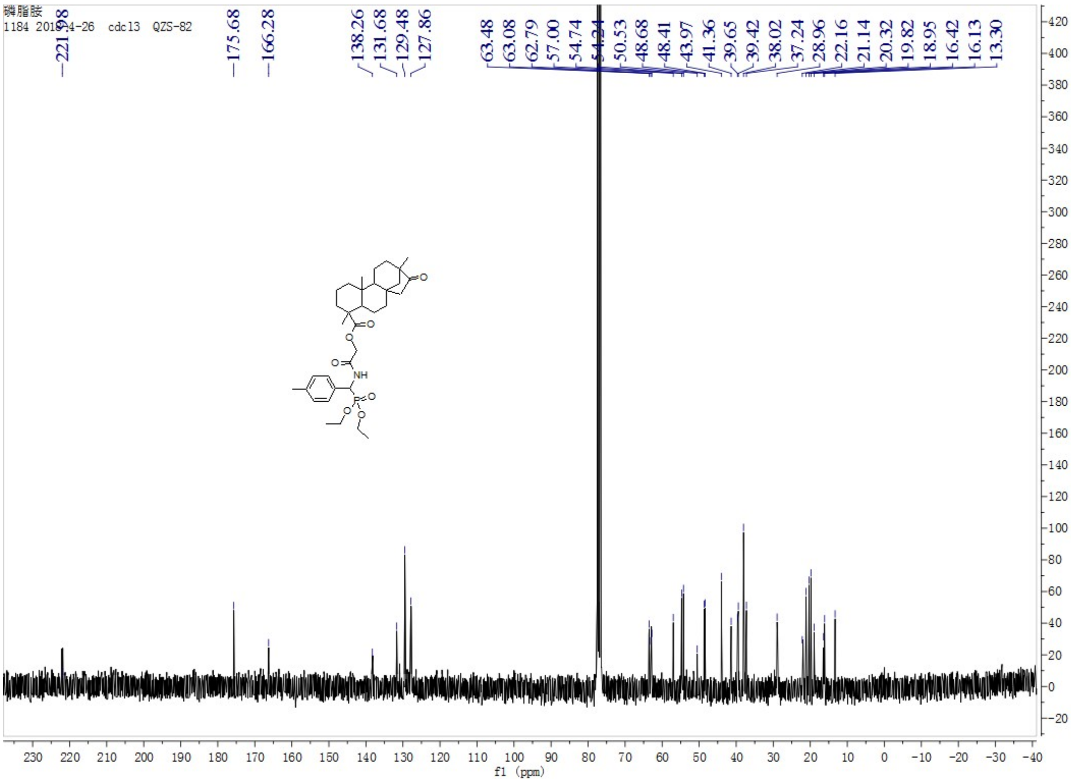

^{1}H NMR and ^{13}C NMR spectra of compound **6d**

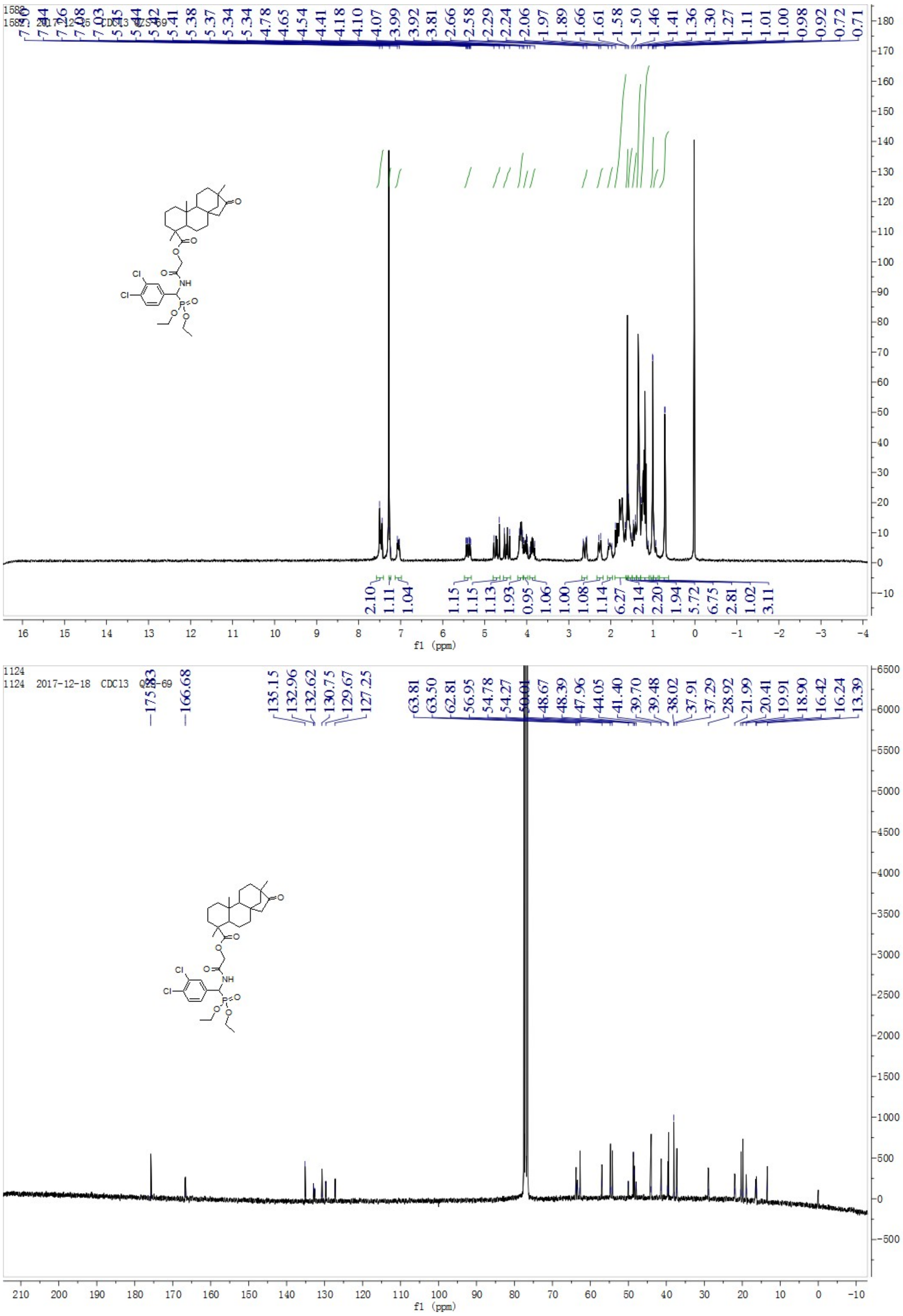

^{1}H NMR and ^{13}C NMR spectra of compound **6e**

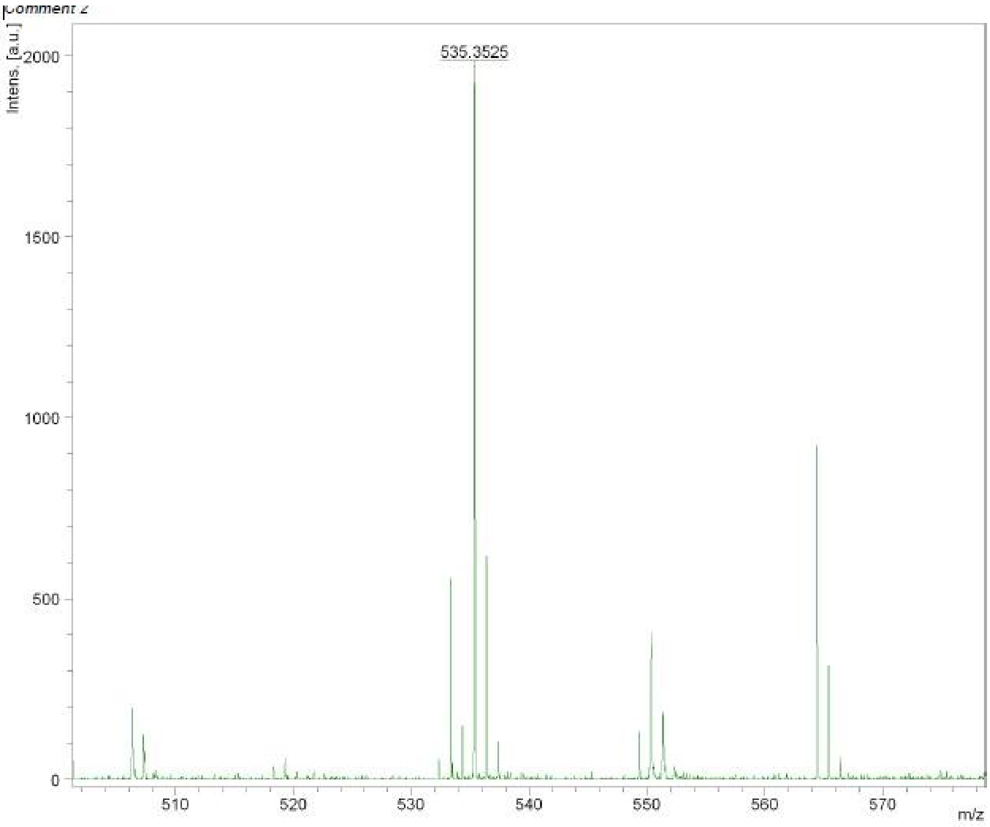

Acquisition Parameter

Date of acquisition	2018-06-23T13:00:40.158+08:00
Acquisition method name	D:\Methods\flexControlMethods\gc-RP_100-1500_Da.par
Aquisition operation mode	Reflector

HRMS spectra of compound **2d**

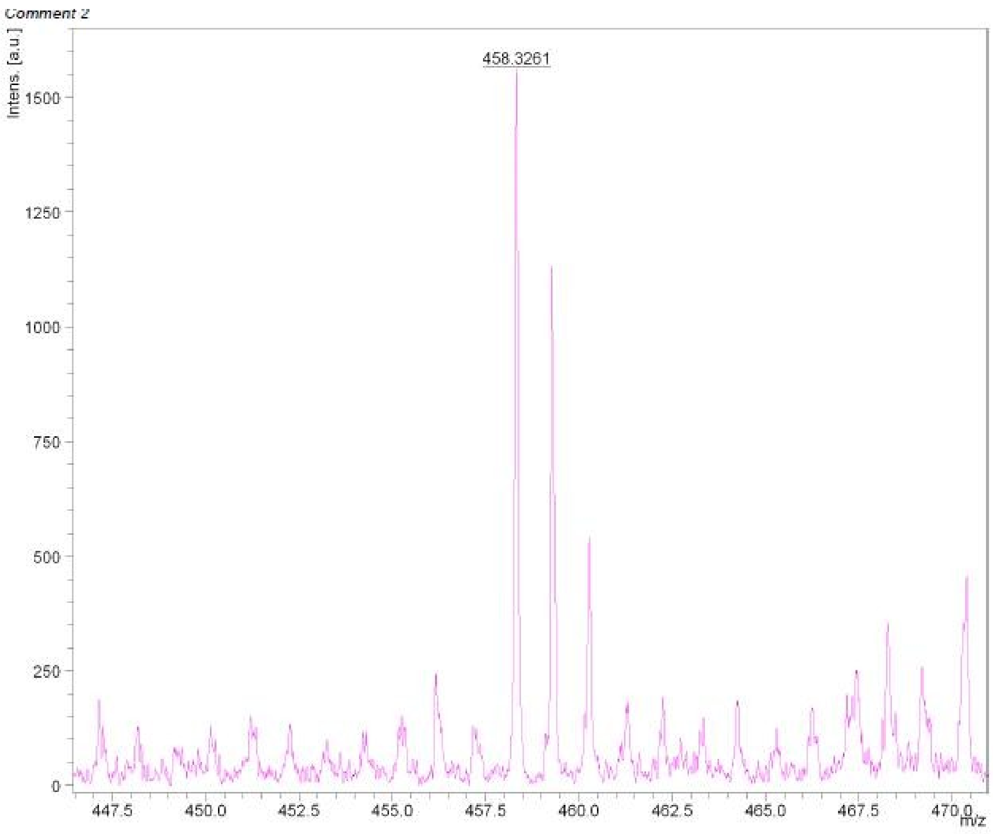

Acquisition Parameter

Date of acquisition	2018-06-23T13:01:59.814+08:00
Acquisition method name	D:\Methods\flexControlMethods\gc-RP_100-1500_Da.par
Aquisition operation mode	Reflector

HRMS spectra of compound **2e**

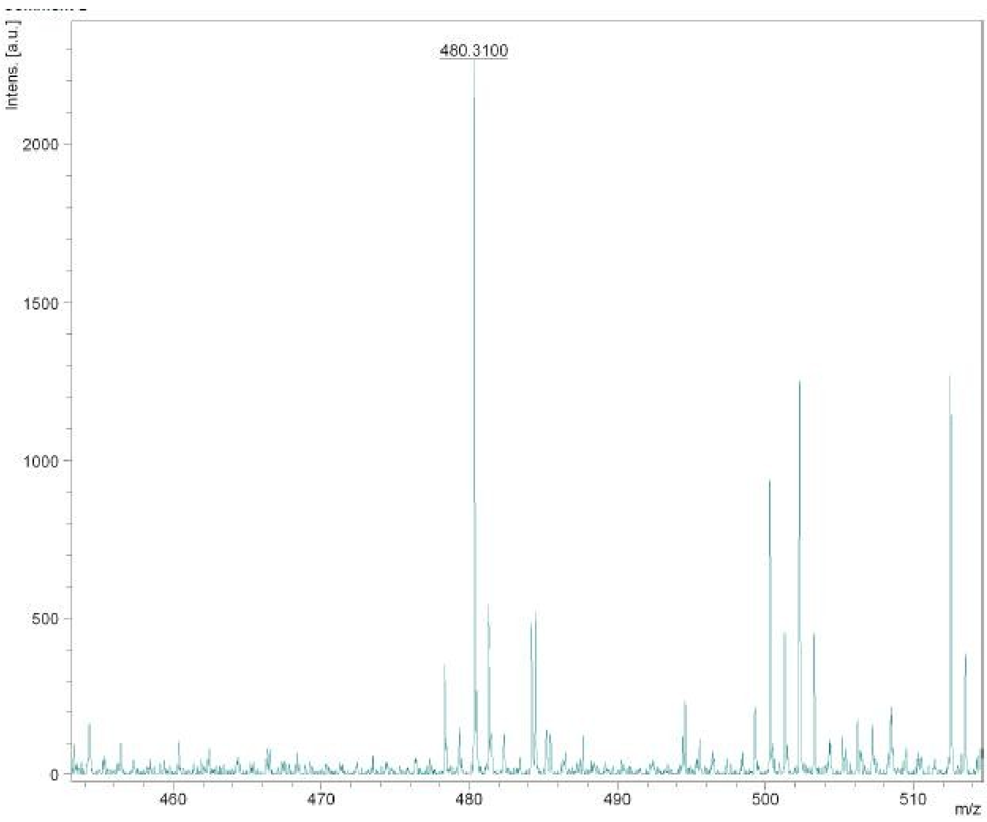

Acquisition Parameter

Date of acquisition	2018-06-23T12:15:14.908+08:00
Acquisition method name	D:\Methods\flexControlMethods\gc-RP_100-1500_Da.par
Aquisition operation mode	Reflector

HRMS spectra of compound **3b**

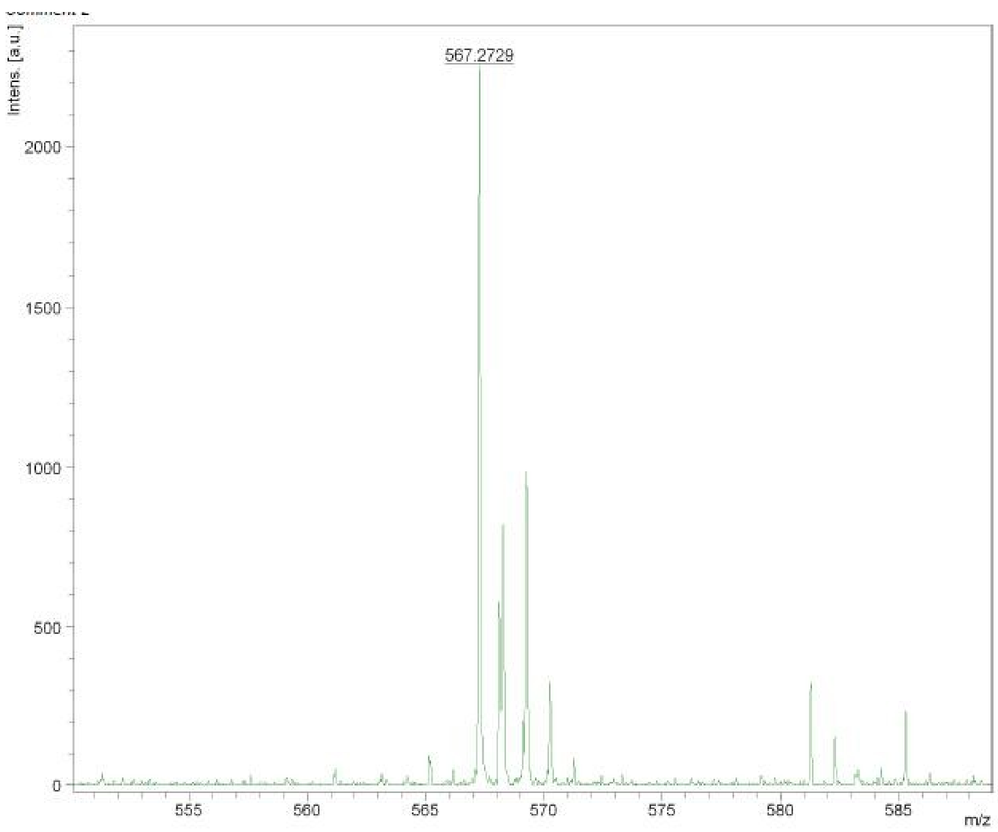

Acquisition Parameter

Date of acquisition	2018-06-20T20:10:16.411+08:00
Acquisition method name	D:\Methods\flexControlMethods\gc-RP_100-1500_Da.par
Aquisition operation mode	Reflector

HRMS spectra of compound **5b**

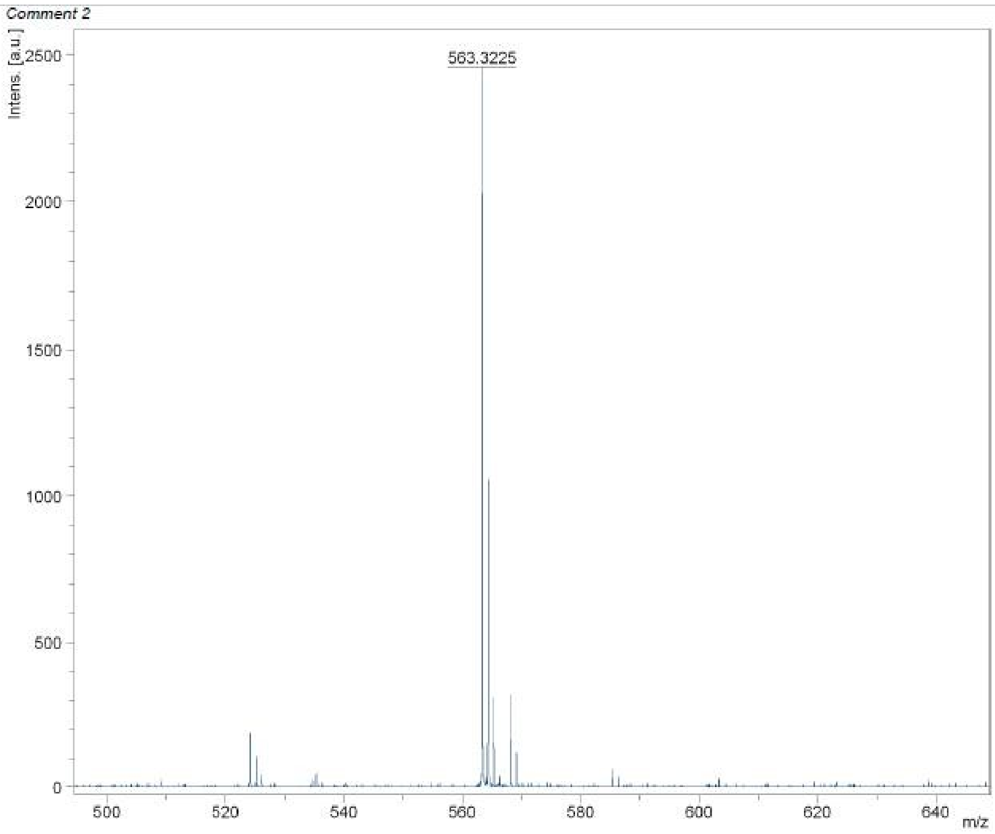

Acquisition Parameter

Date of acquisition	2018-06-20T20:10:40.427+08:00
Acquisition method name	D:\Methods\flexControlMethods\gc-RP_100-1500_Da.par
[illegible]	[illegible]

HRMS spectra of compound **5c**

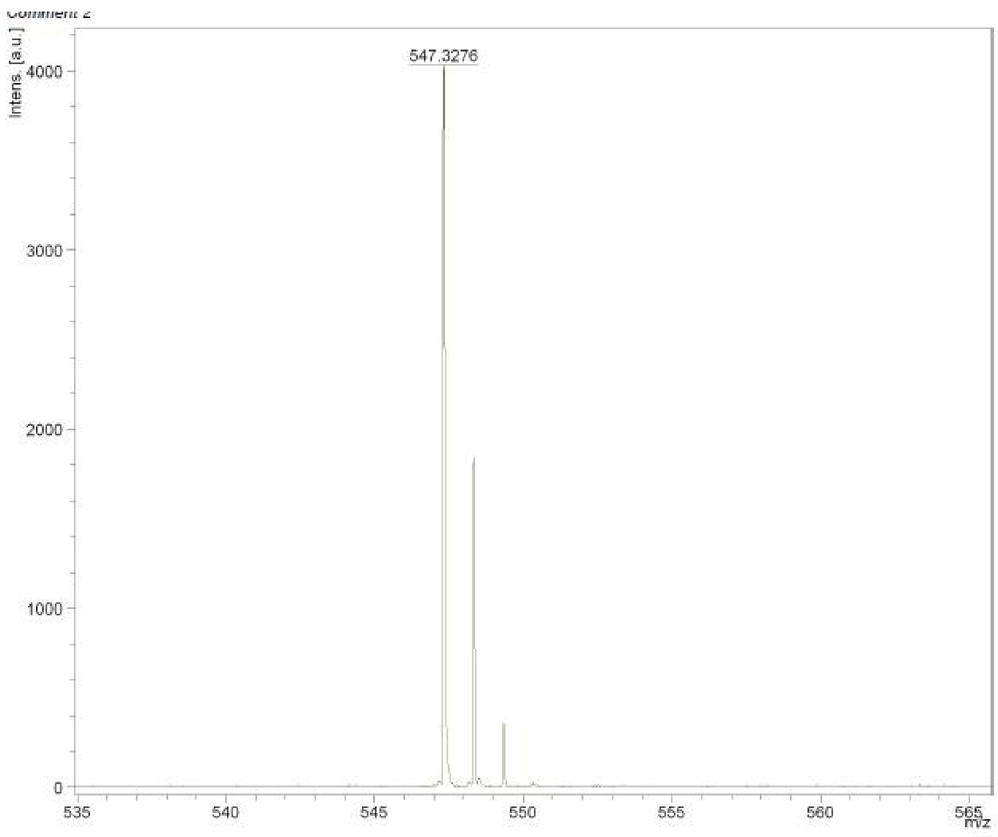

Acquisition Parameter

Date of acquisition	2018-06-20T20:10:50.974+08:00
Acquisition method name	D:\Methods\flexControlMethods\gc-RP_100-1500_Da.par
Aquisition operation mode	Reflector

HRMS spectra of compound **5d**

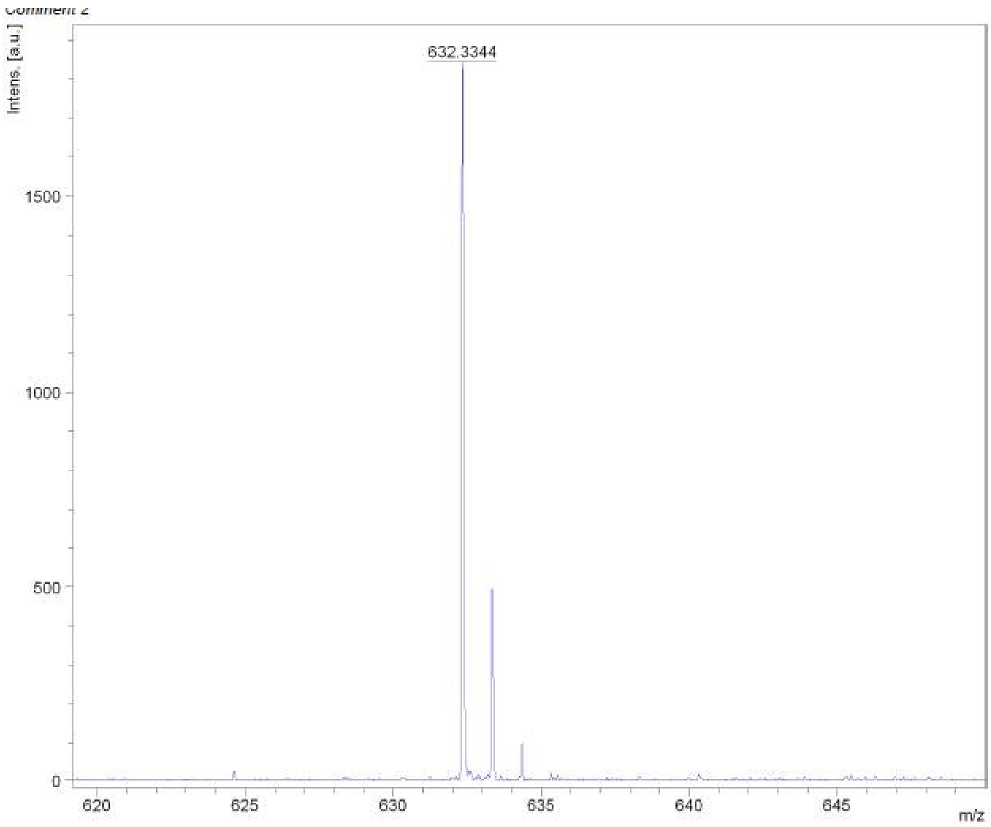

Acquisition Parameter

Date of acquisition	2018-06-23T13:03:24.424+08:00
Acquisition method name	D:\Methods\flexControlMethods\gc-RP_100-1500_Da.par
Aquisition operation mode	Reflector

HRMS spectra of compound **6c**

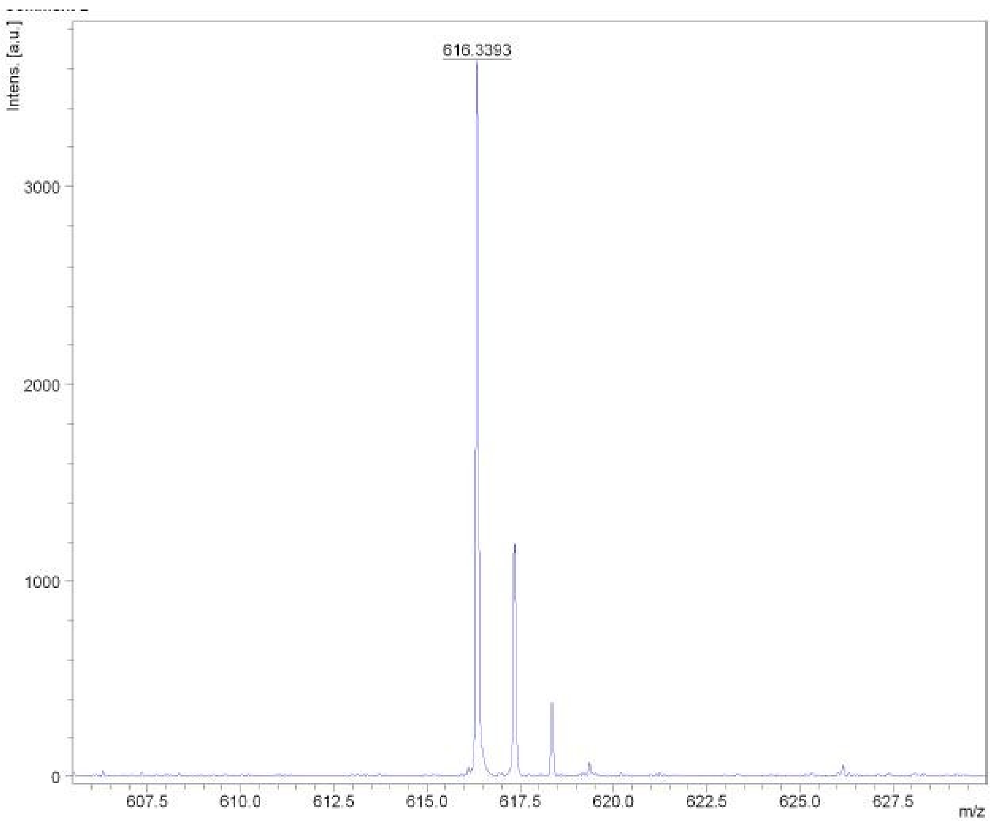

Acquisition Parameter

Date of acquisition	2018-06-20T20:12:12.911+08:00
Acquisition method name	D:\Methods\flexControlMethods\gc-RP_100-1500_Da.par
Aquisition operation mode	Reflector

HRMS spectra of compound **6d**

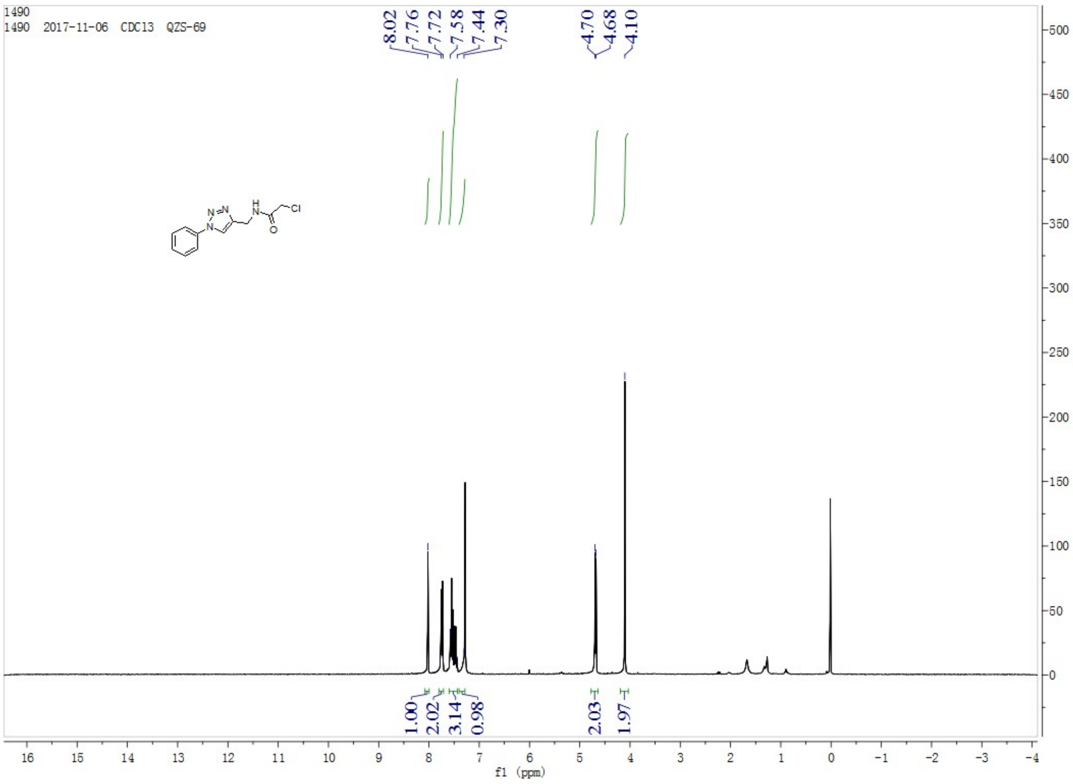

^{1}H-NMR spectra of intermediate **Va**

^{1}H-NMR ($CDCl_3$, 300MHz, ppm): δ 8.02 (s, 1H, triazole-H), 7.76-7.72 (m, 2H, Ar-H), 7.58-7.44 (m, 3H, Ar-H), 7.30 (brs, 1H, -NH-CO-), 4.69 (d, *J*= 6 Hz, 2H, -C$\underline{H}_2$-NH-), 4.10 (s, 2H, -C$\underline{H}_2$-Cl).
